# A Recombination Hotspot in a Schizophrenia-Associated Region of *GABRB2*

**DOI:** 10.1371/journal.pone.0009547

**Published:** 2010-03-08

**Authors:** Siu-Kin Ng, Wing-Sze Lo, Frank W. Pun, Cunyou Zhao, Zhiliang Yu, Jianhuan Chen, Ka-Lok Tong, Zhiwen Xu, Shui-Ying Tsang, Qiang Yang, Weichuan Yu, Vishwajit Nimgaonkar, Gerald Stöber, Mutsuo Harano, Hong Xue

**Affiliations:** 1 Department of Biochemistry, Hong Kong University of Science and Technology, Hong Kong Special Administrative Region, People's Republic of China; 2 Applied Genomics Center, Fok Ying Tung Graduate School, Hong Kong University of Science and Technology, Hong Kong Special Administrative Region, People's Republic of China; 3 HKH Bioinformatics Center, Hong Kong University of Science and Technology, Hong Kong Special Administrative Region, People's Republic of China; 4 Department of Computer Science and Engineering, Hong Kong University of Science and Technology, Hong Kong Special Administrative Region, People's Republic of China; 5 Department of Electronic and Computer Engineering, Hong Kong University of Science and Technology, Hong Kong Special Administrative Region, People's Republic of China; 6 Departments of Psychiatry and Human Genetics and Graduate School of Public Health, University of Pittsburgh School of Medicine, Pittsburgh, Pennsylvania, United States of America; 7 Department of Psychiatry and Psychotherapy, University of Würzburg, Würzburg, Germany; 8 Department of Neuropsychiatry, Kurume University School of Medicine, Fukuka, Japan; University of Poitiers, France

## Abstract

**Background:**

Schizophrenia is a major disorder with complex genetic mechanisms. Earlier, population genetic studies revealed the occurrence of strong positive selection in the *GABRB2* gene encoding the β_2_ subunit of GABA_A_ receptors, within a segment of 3,551 bp harboring twenty-nine single nucleotide polymorphisms (SNPs) and containing schizophrenia-associated SNPs and haplotypes.

**Methodology/Principal Findings:**

In the present study, the possible occurrence of recombination in this ‘S1–S29’ segment was assessed. The occurrence of hotspot recombination was indicated by high resolution recombination rate estimation, haplotype diversity, abundance of rare haplotypes, recurrent mutations and torsos in haplotype networks, and experimental haplotyping of somatic and sperm DNA. The sub-segment distribution of relative recombination strength, measured by the ratio of haplotype diversity (H_d_) over mutation rate (θ), was indicative of a human specific Alu-Yi6 insertion serving as a central recombining sequence facilitating homologous recombination. Local anomalous DNA conformation attributable to the Alu-Yi6 element, as suggested by enhanced DNase I sensitivity and obstruction to DNA sequencing, could be a contributing factor of the increased sequence diversity. Linkage disequilibrium (LD) analysis yielded prominent low LD points that supported ongoing recombination. LD contrast revealed significant dissimilarity between control and schizophrenic cohorts. Among the large array of inferred haplotypes, H26 and H73 were identified to be protective, and H19 and H81 risk-conferring, toward the development of schizophrenia.

**Conclusions/Significance:**

The co-occurrence of hotspot recombination and positive selection in the S1–S29 segment of *GABRB2* has provided a plausible contribution to the molecular genetics mechanisms for schizophrenia. The present findings therefore suggest that genome regions characterized by the co-occurrence of positive selection and hotspot recombination, two interacting factors both affecting genetic diversity, merit close scrutiny with respect to the etiology of common complex disorders.

## Introduction

Schizophrenia is one of the most disabling mental disorders, occurring worldwide at about 1% incidence [1], [2], [3]. The long arm of human Chromosome 5 was known for some time to be genetically linked to schizophrenia [4]. More recently, schizophrenia-association was established for *GABRB2* located at chromosome 5q34, and its genotypes have been correlated to splicing variations of β_2_-subunit isoforms that give rise to different electrophysiological consequences in type A γ-amino-butyric acid (GABA_A_) receptor function [5], [6], [7]. This *GABRB2*-schizophrenia association has since been validated by independent studies on multiple ethnic groups [5], [8], [9], [10].

**Figure 1:**
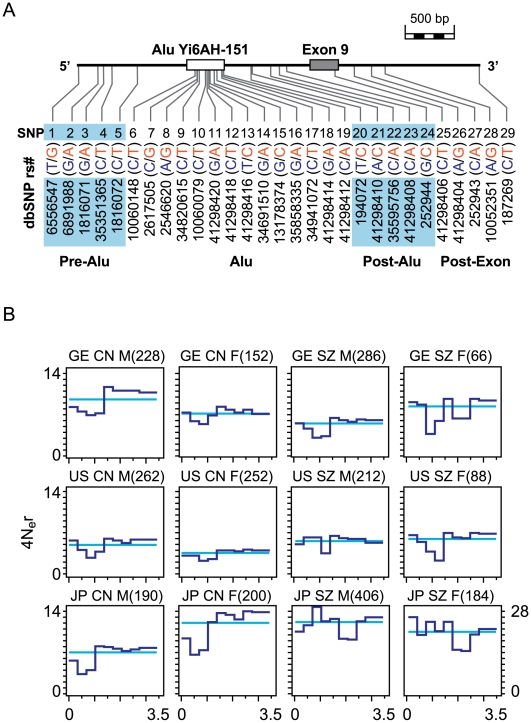
Distribution of SNPs and recombination rate in the S1-S29 segment of *GABRB2*. (A) Locations and allelic forms (blue for ‘ancestral’ and red for ‘derived’ as described in [11]) of SNPs in the S1–S29 segment (chromosome 5q34, 160,689,203-160,692,753 bp). (B) Population recombination rates estimated by Hotspotter. The profile of the estimated population recombination rate is shown as ρ_hat_ (“rhohat” output from Hotspotter shown in dark blue), or the estimated average population recombination rate ρ_bar_ (“rhobar” output from Hotspotter shown in light blue). The JP SZ-F cohort gave a ρ_bar_ value of 21.3, corresponding to a recombination rate of 15.0 cM/Mb. The number of chromosomes analyzed in the control (CN) or schizophrenic (SZ) male (M) or female (F) German Caucasian (GE), American Caucasian (US) or Japanese (JP) cohort is indicated by the number inside parentheses following cohort identification.

The schizophrenia-associated S1–S29 segment (3,551 bp) of *GABRB2* (Figure 1A) contains a human specific Alu-Yi6 element, and SNPs that were found to be subject to positive selection [11]. Subsequently the suggestion was also made that positive selections might have acted as well on other schizophrenia-associated genes besides *GABRB2* [12]. In view of the known Alu-enhancement of genetic polymorphisms [13], [14], the role of recombinations in shaping this S1–S29 segment has been analyzed in the present study.

In the present study, population genetics analyses based on re-sequencing of S1–S29 segment yielded ultra-high resolution sequence diversity parameters and recombination rate estimates for schizophrenia and control cohorts from different ethnic groups. In haplotype analysis, special attention was directed to the identification of schizophrenia risk-conferring and protective haplotypes. Experimental haplotyping and sperm-typing were performed to provide direct evidence of recombination activity. The structural basis of high-level recombination in the region was examined in terms of local DNA conformation and insertion of an AluY element, and the significance of the observed co-existence of positive selection and hotspot recombination was explored.

## Results

**Figure 2:**
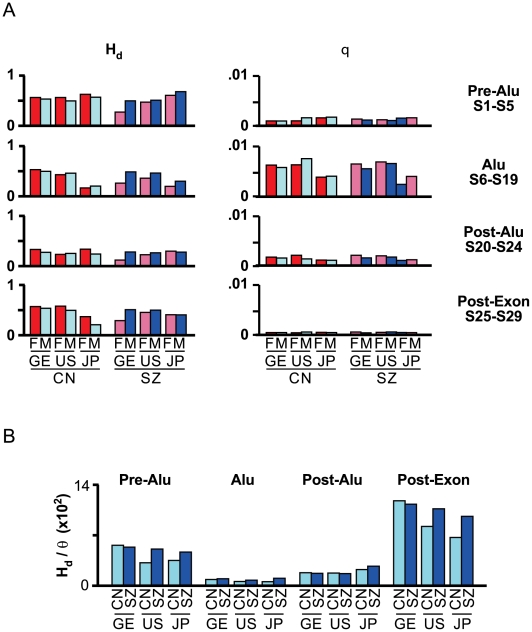
Sequence diversity in different sub-segments of S1–S29. (A) Sequence diversity profiles. Haplotype Diversity (H_d_) and the mutation rate, Watterson's Theta (θ) were calculated for control males and females in GE, US and JP populations, and in the latter three for schizophrenic males and females (Table S1). (B) Recombination strength measured by H_d_/θ for four SNP-containing sub-segments in S1–S29 sequence distinguished based on their positions relative to Alu Yi6AH-151 and Exon 9: Pre-Alu (S1–S5), Alu (S6–S19), Post-Alu (S20–S24) and Post-Exon (exonic synonymous S25 plus post-exonic S26–S29).

Direct sequencing of the S1–S29 segment in GE, US and JP cohorts, and data analysis using Hotspotter [15] yielded a recombination rate for the JP SZ-F cohort falling within the recombination hotspot range of 10–120 fold the genome average recombination rate of 0.89 cM/Mb [16], [17]; the rates for other GE, US and JP cohorts also approached this range (Figure 1B). This S1-S29 segment in fact belonged to one of the three major recombination clusters in the *GABRB2* gene displaying enhanced recombination in the four HapMap populations (Figure S1). AF samples, more limited in sample size, are presented in Figure S2. Haplotype diversity (H_d_) [18] and mutation rate (θ) [19] were high and non-uniform across sub-segments of S1–S29 differentiated according to their locations in relation to Alu-Yi6AH-151 and Exon 9 (Figure 2A). Although θwas lower in Pre-Alu and Post-Exon compared to the Alu region, H_d_ in Pre-Alu and Post-Exon exceeded that in the Alu region, strongly suggesting that recombination played an important role in the generation of high haplotype diversity. When the H_d/_θ ratio was calculated to provide a first-approximation measure of recombination strength (Figure 2B), the Post-Exon (S26–S29 plus the S25 synonymous SNP) displayed the highest H_d_/θscores, followed in descending order by Pre-Alu (S1–S5), Post-Alu (S20–S24) and Alu (S6–S19) for both the control (CN) and schizophrenic (SZ) cohorts of all three populations. Since the minimum efficient processing segment is about 200 bp in mitotically dividing cells [20], the higher H_d_/θ scores of Post-Exon located at about 1,000 bp from the Alu, and Pre-Alu at about 450 bp from the Alu, relative to Post-Alu at just over 200 bp from the Alu, and even more so the Alu sub-segment itself are consistent with Alu-Yi6AH-151 serving as a recombination initiation site. This identification of the human specific Alu-Yi6 as a recombining centre is consistent with its location in the midst of the S1-S29 SNP cluster, which spans about 3,551 bp, close to the usual 1–2 kb lengths of recombination hotspots [21].

**Figure 3:**
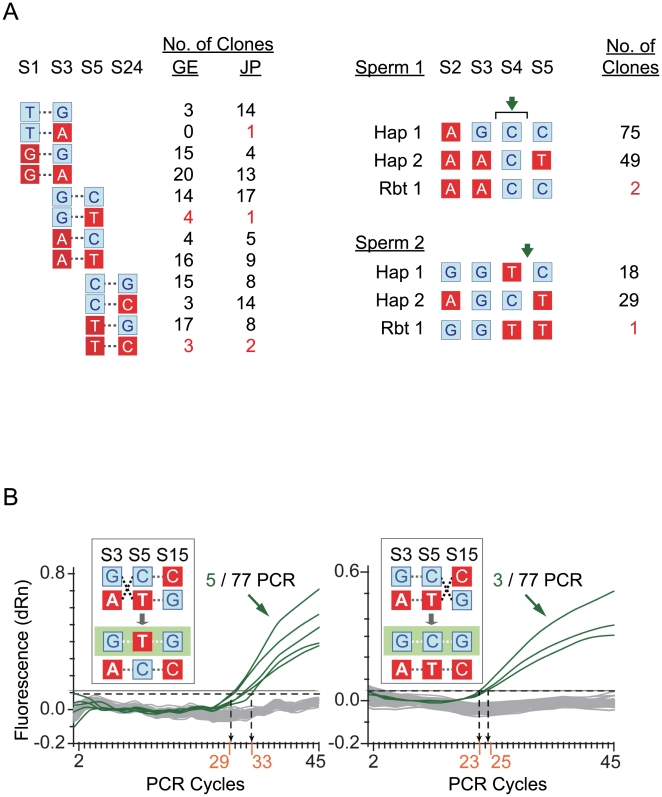
Experimental detection of recombination events in S1–S29. (A) Haplotyping of somatic and sperm DNA. Left: Haplotyping of somatic DNA from 16 JP controls and 19 GE controls (Methods) selected according to the criterion that each sample was heterozygous for at least two of SNPs S1, S3, S5 and S24. DNA sequence containing these four SNPs was in each instance amplified from first PCR product and sequenced (Methods), and crossover points between the S1–S3, S3–S5 or S5–S24 SNP sites were identified based on the four-gamete rule [40]. The maximum distances of these crossover points from AluYi6-AH151 were 815-bp, 763-bp and 540-bp, respectively, in keeping with this young human-specific Alu being a recombination center [11]. Right: Haplotyping of sperm DNA from two Chinese *Han* individuals without any history of psychiatric disorder. PCR products covering the S2–S5 segment inclusive were each cloned into a pMD18-T vector, and inserts from individual colonies were haplotyped by DNA sequencing covering the S2–S5 sites. For both Sperm 1 and Sperm 2, two high frequency haplotypes (Hap 1 and Hap 2) were encountered among the colonies which were taken to represent the non-recombinant haplotypes, and one low frequency haplotype (Rbt 1) was encountered which was taken to represent a recombinant haplotype. On this basis, the presence of two Rbt 1 clones in Sperm 1 and one Rbt 1 clone in Sperm 2 confirmed the occurrence of meiotic recombinations in both sperm samples. (B) Haplotyping by allele-specific real-time PCR was performed on Sperm 1 DNA to detect crossovers between its G-C-C and A-T-G somatic S3-S5-S15 haplotypes. Detection was based on the accumulation (green tracings) of the green-shaded recombinant haplotype signaled by increase in fluorescence through the incorporation of the 6FAM-labelled PCR primer B218-ASPA-T1 (Table S8), yielding 5 detections of the G-T-G haplotype arising from recombination between S3–S5 (left) and 3 detections of the G-C-G haplotype arising from recombination between S5–S15 (right) out of 77 runs in each case with approximately 100 amplifiable genomes, which corresponded to recombination rates of 129 cM/Mb and 94 cM/Mb respectively (Table S4). The PCR cycle thresholds (Ct) for positive signal are numbered in orange. As in the case of Figure 1A, ancestral alleles in this figure shown in blue, and derived alleles in red.

Experimental haplotyping by cloning and sequencing of somatic DNA employing S1, S3, S5 and S24 as markers revealed 11/210 recombinant clones, even when only the lowest-frequency doublets in lines 2, 6 and 12 in Figure 3A were counted as recombinants. Haplotyping by cloning and sequencing of sperm DNA from two donors likewise showed recombination occurring within each of the donors: Sperm-1 DNA yielded 2/126 S3–S5 recombinants, and Sperm-2 DNA 1/48 S3–S5 recombinants (Figure 3B). Furthermore, when the S3–S5–S15 haplotypes in Sperm 1 DNA were analyzed by allele-specific real-time PCR, the recombination observed between S3–S5 and between S5–S15 yielded hotspot level recombination rates of 129 cM/Mb and 94 cM/Mb respectively. Therefore molecular genetic evidence for the occurrence of recombination events within the S1–S29 segment were readily obtainable from both somatic and sperm DNA, confirming the recombination hotspot nature of this region as suggested by Figures 1B and 2B.

**Figure 4:**
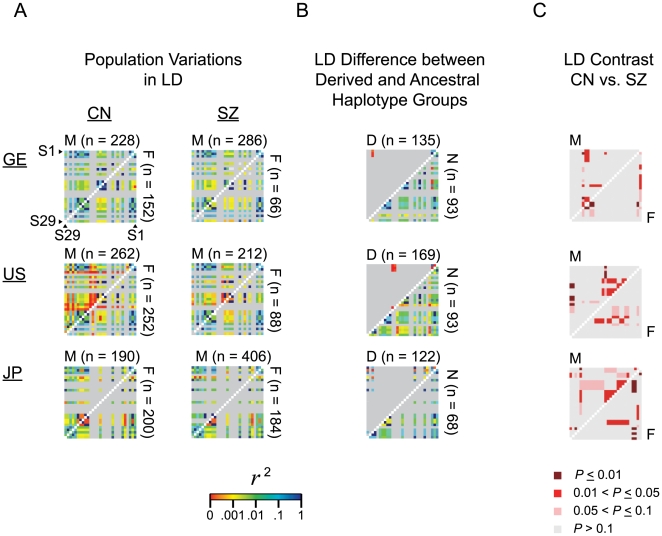
Linkage disequilibrium (LD) analysis. (A) Detection of recombination in LD-plots. Haplotype forms were inferred from genotype data of the samples using PHASE version 2.1. One thousand resamplings were performed for the haplotype set of each cohort. Different haplotype forms are shown in Table S2. Coefficients of LD (*r* ^2^) estimated by DnaSP [43] for each SNP pair (Table S5) are displayed in thermal scale (shown at bottom of figure) for available male (M, upper triangles) and female (F, lower triangles) CN and SZ samples in GE, US and JP populations. (B) LD-plots for derived (D) and ancestral (N) haplotypes for GE, US and JP Males in same thermal scale, with D- and N-haplotypes being distinguished based on the allelic form of S5 contained in the haplotype (Table S6). (C) LDcontrast [44] between CN and SZ derived from the corresponding CN and SZ plots for GE, US and JP groups from part (A). The *P* values pertaining to the CN versus SZ comparisons (Table S7) are plotted for M (upper triangles) and F (lower triangles) cohorts in color-coded ranges as shown below the plots: significant CN-SZ difference is indicated by a red (for 0.01<*P*≤0.05) or dark red (for *P*≤0.01) square for each CN-SZ comparison.

When linkage disequilibrium (LD) was analyzed for different human population cohorts, the prominence of low LD points (red colored on thermal scale) in the GE, US and JP cohorts in Figure 4A pointed to ongoing recombination events in these populations. When haplotypes containing the ancestral (N) allele of SNP S5 were compared to haplotypes containing the derived (D) allele in Figure 4B, the D-haplotypes exhibited a limited number of break points a fair number of which were low-LD, compared to the many mostly higher-LD points (green-blue colored on thermal scale) exhibited by the N-haplotypes. This is in accord with the intense positive selection of the D-alleles of S5 along with S1, S3, and S29 [11]. Figure 4A also displayed recognizable dissimilarities between males and females. The LD contrasts shown in Figure 4C revealed a number of significant differences between the CN and SZ groups at the *P*≤0.01 level. Previously, LD mapping has also located promising SZ-CN differences in the genes for neuregulin-1, dysbindin, and proline dehydrogenase [22], [23], [24], [25], [26].

**Figure 5:**
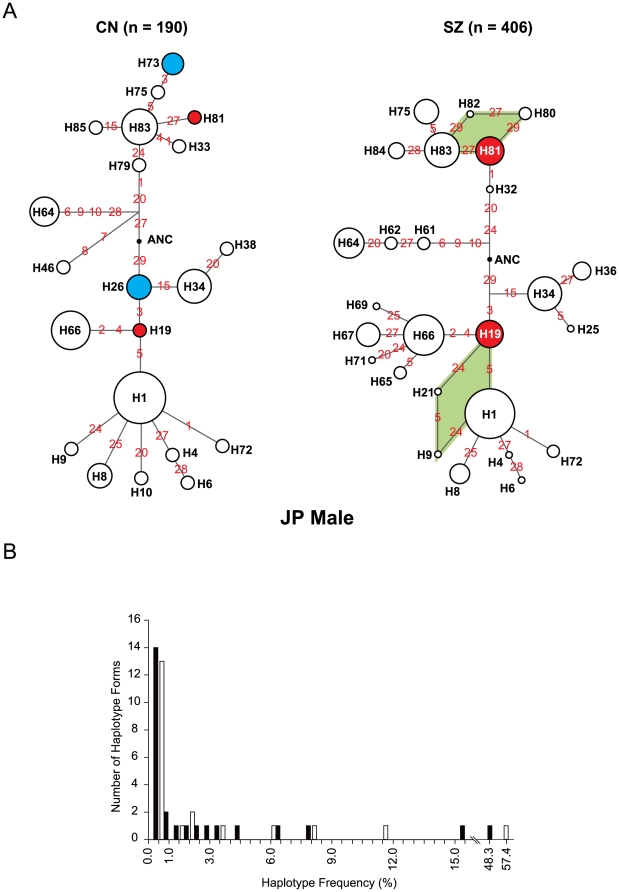
Diversity of haplotypes. (A) Haplotype networks. The networks of mutational steps linking the haplotype forms encountered in the control (CN) or schizophrenia (SZ) group of JP males were generated using the NETWORK program [45]. SNPs are represented by their respective numbers in red without the prefix “S” (“1” for S1, etc) along the inferred mutational paths joining the nodes that are numbered in black and represent various haplotypes. The compositions of different haplotypes/nodes are given in Table S2, and the frequencies of different haplotypes/nodes including those in the JP Male CN and SZ networks are given in Table S3. The radius of a node in the figure followed the log of the normalized frequency of that node within its network. The hypothetical ancestral haplotype (ANC), comprising the ancestral alleles of all 29 SNPs, is shown by a black dot in the networks. Haplotype H1, which constituted the largest node in the networks of all the population cohorts, comprises the derived alleles of S1, S3, S5, S29, and the ancestral alleles of the other 25 SNPs. The nodes representing the protective haplotypes H26 and H73 are colored blue whereas the risk-conferring haplotypes H19 and H81 are colored red. The appearance of the same SNP at different locations of the network points to recurrent mutations at this SNP site likely as a consequence of recombination events, e.g. S27 appearing at three locations in the CN network and six locations in the SZ network. The green patches highlight torsos consisting of alternate recombination pathways. (B) Abundance of rare haplotype forms. The numbers of different haplotype forms in the JP male CN cohort occurring at various frequencies as given in Table S3 are represented by white bars, and those in the JP male SZ cohort by black bars. There were 13/190 rare haplotypes with a frequency of ≤1% in the CN cohort, and 14/406 in the SZ cohort.

The frequent occurrence of recombination in the S1–S29 segment was also suggested by network analysis of inferred haplotypes. In Figure 5A the nodes (numbered in black) represent different haplotypes found in the JP male CN and SZ cohorts, and each line linking two nodes bears the identity of the SNP(s) (shown in red) where a mutational change would be required to convert one haplotype to the other. The presence of torsos in the networks (green shaded), and the participation of SNPs such as S27 at multiple locations in both the CN and SZ networks, furnished evidence for the occurrence of recurrent mutational changes, which likely implicated recombination events more than point mutations. Through enhanced recombination, the haplotype networks displayed a high level of haplotype diversity (see also Tables S2 and S3). The relevance of haplotype diversity to schizophrenia is consistent with the presence of the green-shaded torsos around H80 and H21, and the branches radiating from H66, in the SZ but not in the CN network. The strong tendency, likely owing to hotspot recombination, to generate rare haplotype forms in the S1–S29 segment even in the presence of intense positive selection of some of the SNPs [11] was indicated by the presence of numerous low frequency (≤1%) haplotypes amounting to over 60% of the haplotype forms in either the CN or the SZ cohort of JP males (Figure 5B).

In JP males there were 4 instances of haplotype H26 in the CN cohort but none in the SZ cohort, yielding a significant difference with *P_U_* = 0.00335 based on the log-likelihood ratio test in UNPHASED, *P_C_* = 0.00242 based on Chi-square, or *P_P_* = 0.000123 based on *Poisson* distribution (see Tables S3B-E for methods of statistical calculations), between these two cohorts. Likewise, there were in GE males 5 instances of H26 in CN but only one in SZ (*P_U_* = 0.0532, *P_C_* = 0.0391, *P_P_* = 0.0174); and in JP males 3 instances of H73 in CN but none in SZ (*P_U_*  = 0.0111, *P_C_* = 0.0136, *P_P_ = *0.00248). These findings suggest that H26 and H73 are protective against the development of SZ. On the other hand, there was in JP males one instance of H19 in CN but 12 instances in SZ (*P_U_* = 0.0585, *P_C_* = 0.0381, *P_P_* = 0.0174); in Japanese males there was one instance of H81 in CN but 14 instances in SZ (*P_U_* = 0.0338, *P_C_* = 0.0208, *P_P_* = 0.00729); and in Japanese females there was no H81 in CN but 6 instances in SZ (*P_U_* = 0.0101, *P_C_* = 0.00708, *P_P_* = 0.000912). These findings suggest that H81 and possibly H19 are risk-conferring toward the development of SZ. Notably, in the JP male cohort, as many as 13 SZ subjects (one homozygous and 12 heterozygous) out of a total of 203 SZ subjects, or 6.4%, displayed the risk-conferring H81.

**Figure 6:**
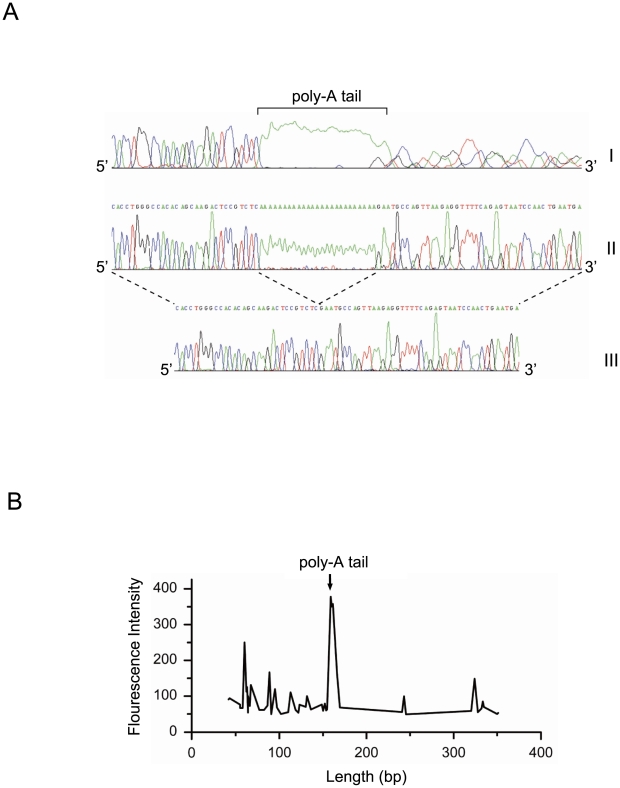
DNA conformational irregularities around AluYi6-AH151. (A) Panel I shows sequencing read of linear PCR product generated from S1–S29 genomic region serving as template. The sequence 3′ to the Alu poly-A tail could not be read properly. In Panel II, when the same sequence in Panel I was cloned and subsequently sequenced from the circular pMD18-T vector, its circular conformation allowed proper reading of the sequence 3′ to the Alu poly-A tail. In Panel III, the Alu poly-A tail had been deleted, and the region 3′ to Alu could be read even with linear PCR product serving as sequencing template. (B) DNase I sensitivity assay on fluorescent 6FAM-labelled PCR products containing Alu Yi6-AH151 (Methods), showing site of enhanced nuclease-sensitivity at the start of the Alu poly-A tail.

Anomalous conformational effects exerted by the Alu-Yi6 element on genomic structures were revealed by the blockade of sequencing by polymerase across the Alu-Yi6 element inside a free-ended template (Figure 6A), which was surmountable by placing the template into a circular vector or by excision of the poly-A tail from the Alu-Yi6. The pinpointing of the poly-A tail as a conformation-disrupting site by its excision was further confirmed by the finding that the poly-A tail was highly sensitive to DNase I sensitive digestion (Figure 6B). Such anomalous destabilization of genomic structures by Alu elements, especially those of the youngest Alu-Y family, is known to cause enrichment of SNPs in their vicinities [13], [14], and may contribute in the present instance to the high sequence diversity of the S1–S29 segment.

## Discussion

Based on the findings in the present study, the S1–S29 segment of *GABRB2* has been identified as a recombination hotspot despite the occurrence of positive selection [11]. Complete sequencing of the segment yielded ultra-high resolution recombination rate estimates, which were clearly elevated in the GE and US Caucasian as well as JP cohorts. In an attempt to obtain an estimated population recombination rate profile, Hotspotter [15] was employed, yielding rate peaks along the segment (Figure 1B).

In addition, the H_d_/θ ratio was used to determine recombination strength against mutational background in the different sub-segments of S1–S29 in the different population cohorts. This application is based on the fact that, except for the major portion of the Y chromosome and mitochondrial DNA, haplotype diversity H_d_ in the human genome is influenced by both recombination and mutation, whereas the population mutation parameter θ depends on the mutation-drift equilibrium in nucleotide diversity. The ratio H_d_/θ is therefore a useful parameter for tracking the net effect of recombination.

In accord with the observed elevated recombination rates, rare forms of haplotypes with <1% frequencies were plentiful among both patients and controls (Figure 5B), and low LD points (red dots on LD plots in Figure 4B) indicative of recent recombination crossover sites were observed especially among the derived haplotypes (Figure 4). Haplotype network analysis also showed torsos as well as recurrent mutations, both of which pointed to a high frequency of recombination (Figure 5A), and the occurrence of hotspot recombination was confirmed by molecular genetics experiments based on haplotyping and sperm-typing (Figure 3).

With respect to the structural basis for the recombination hotspot nature of the S1–S29 segment, sub-segment recombination strengths relative to mutation rate as measured by H_d_/θ (Figure 2A) suggested that Alu Yi6AH-151, a human-specific Alu insertion near the center of the segment (Figure 1A), could serve as a recombining sequence for homologous recombination. Since a minimal efficient processing segment for recombination crossover is about 200 bp in mitotically dividing cells [20], that recombination crossovers were observed in the sequences flanking Alu Yi6AH-151 rather than the Alu itself (Figure 3) was consistent with such a central role for the Alu. The poly-A tail of this Alu sequence was found to cause DNA conformational anomaly (Figure 6) that could contribute to local sequence diversity and genomic instability, resulting in for example an open chromatin state promoting double-strand break initiation for recombination [27]. Moreover, Alu elements are known to be enriched in GC-rich regions of the human genome, where recombination events are often found [28].

As previous modeling studies indicated, positive selection is more readily achieved in the presence of recombination by rendering the selected site independent of neighboring sites [29]. Thus, the possible occurrence of positive selection suggested earlier for the S1–S29 segment in *GABRB2* [11] could be facilitated by the recombination hotspot nature of the segment demonstrated in the present study. Such facilitation could be particularly significant in the case of rapid evolutionary changes such as the emergence of *Homo sapiens*. In turn, the positive selection, indicative of the functional importance of the segment, would bring about fast rising frequencies of the selected genotypes, which together with hotspot recombination generated novel common haplotypes. Among them, H1 (in Table S2, or designated H56 in [11]) eventually became a dominant haplotype in the evolving human lineage. Prominent diversity in haplotypes, including both rare (frequency <5%) and common forms of haplotypes, are evident from the frequency plot in Figure 5B and the catalog of haplotypes in Table S2. By bringing about the generation of novel haplotypes within a functionally important CNS gene, hotspot recombination could result in significant perturbations of CNS functions that could be relevant to the etiology of schizophrenia. In this light, the association of the S1–S29 segment with schizophrenia, and the identification of both protective (H26 and H73) and risk-conferring (H19 and H81) haplotypes in Figure 5A underline the potential significance of genomic regions simultaneously subjected to positive selection and hotspot recombination toward the development of common complex disorders such as schizophrenia.

Widespread positive selections acting on CNS gene loci that could be essential for the adaptive evolution of human cognitive functions [30] have been reported for a number of schizophrenia candidate genes [12]. Increased fine-scale recombinations have also been described for human conserved non-coding regions undergoing accelerated evolution [31], comparable to those in the S1–S29 segment of *GABRB2*. Low-frequency causative mutations are often balanced by purifying selection in Mendelian diseases. In contrast, at genetic loci associated with complex disease such as schizophrenia, hotspot recombination and positive selection might not merely nullify one another's effects even though they tend to exert opposing effects on genetic diversity. Instead, through their conjoint generation of novel haplotypes of fast increasing frequencies, together they could play an important evolutionary role in common complex disease etiology.

Accordingly, based on the known heritability of recombination hotspots, and the demonstrated schizophrenia relevance of both recombination (Figure 4C) and positive selection (Figure 5 in [11]) in *GABRB2*, recombination-selection co-occurrence could be a potential molecular genetics mechanism contributing to schizophrenia development. Since human recombination hotspots are largely unique to the human genome unshared by the chimpanzee genome [32], [33], the involvement of recombination-selection co-occurrence in schizophrenia etiology is consistent with the fact that schizophrenia is an exclusively human disease [34]. Furthermore, despite the procreative disadvantage of schizophrenia patients, hotspot recombination could maintain a high rate of appearance of haplotypes with negative effects. Thus recombination-selection co-occurrence would help to explain not only the association between *GABRB2* S1-S29 segment with schizophrenia, but also the persistence of a high disease rate of 1% in humans despite the survival disadvantage of the disease, which is commonly recognized as the “central paradox” of schizophrenia [34].

## Materials and Methods

### Ethics Statement

Written informed consent was obtained from each participant. Approvals for the study were obtained from the ethnical committees of Kurume University for the JP samples, of University of Würzburg for the GE samples and of the University of Pittsburgh Institutional Review Board for the US samples.

### Study Cohorts

A total of 1,353 individuals (female 471; male 792), including 732 unrelated non-schizophrenia controls (339 females, 393 males) and 621 schizophrenic patients (169 females; 452 males) from the four different ethnic populations African (AF), German Caucasians (GE), American Caucasians (US) and Japanese (JP), were studied. They included individuals whose genotypes were analyzed in Lo *et al*. [11]

For the AF population, DNA samples of 30 parent-offspring trios from Yoruba in Ibadan, Nigeria (7 female and 23 male offsprings) were obtained from Coriell Cell Repositories (Camden, NJ; Panel ID: HAPMAPPT03). All members of each trio were genotyped. The accuracy in phase estimation of parental haplotypes was confirmed by genotype data from the offspring. Only data of unrelated parents (29 mothers; 30 fathers) were used in the statistical analysis.

For the JP and GE samples, details of sample sources and diagnostic procedures were described in Lo *et al*. [5] In brief, the JP samples consisted of 195 unrelated control subjects (100 females; 95 males) and 295 unrelated schizophrenia patients (92 females; 203 males). The GE samples consisted of 190 unrelated control subjects (76 females; 114 males), and 176 unrelated schizophrenics of the systematic subtype (33 females; 143 males), the most severe form of schizophrenia based on Leonhard's classification of endogenous psychoses [35]. All schizophrenia patients were in-patients and fulfilled the diagnostic criteria for schizophrenia according to the 4th edition of Diagnostic and Statistical Manual of Mental Disorders [36]. The US samples consisted of 257 unrelated controls (126 females; 131 males) and 150 schizophrenia patients (44 females; 106 males). Samples were collected as described [37]. Briefly, the patients were evaluated using the Diagnostic Interview for Genetic Studies semi-structured diagnostic interview scale [38]. This information was combined with medical records and available information from relatives.

### Polymerase Chain Reaction and Ultra-High Resolution Genotyping by Sequencing

The 7.4-Kb genomic region (2,148 bp upstream of Exon 9 to 519 bp downstream of Exon 9) spanning the two schizophrenia-associated SNPs rs6556547 (S1) and rs187269 (S29) in *GABRB2* was generated by PCR and served as first PCR template for amplification of two overlapping nested-PCR products, Fragment A and Fragment [5], [6], [11]. The fragments extended from 160,692,860 bp to 160,688,429 bp of chromosome 5. The detail PCR procedures were described in Lo *et al*. [11]

Resequencings for SNP genotyping were performed from either end of template employing the sequencing primers in Table S8. Each sequencing reaction contained 3 µl of sequencing buffer, 0.5 µl of BigDye Terminator version 3.1 (Applied Biosystems Inc., Foster City, California), ∼100 ng purified nested-PCR products and 1 mM sequencing primer. Each cycle of sequencing reaction consisted of initial denaturation at 96°C for 1 min, followed by 25 cycles each of 10 s at 96°C, 5 s at 50°C, and 4 min at 60°C. Ethanol precipitation was used to clean-up the post-sequencing products as in the case of the PCR products. Each air-dried sequencing sample was dissolved in 10 µl Hi-Deionized Formamide (Applied Biosystems Inc., Foster City, California), denatured at 95°C for 1 min and immediately held at 4°C prior to sequencing with a Model 3100 Genetic Analyzer (Applied Biosystems Inc., Foster City, California).

Sequence chromatogram alignment-based SNP discovery and genotype calling were carried out using the software package PolyPhred version 4.2 [39]. All genotyping results were manually confirmed by at least two researchers independently. All analyzed SNPs were located within the high-quality region (Quality Value ≥20), and occasional low-quality passes were re-sequenced. All observed haplotype sequences were deposited in NCBI Genbank and assigned with accession numbers (Table S2).

### Detection of Recombinations in Sperm DNA by Cloning

To detect meiotic crossing-over events, sperm-typing of two sperm samples were conducted. Sperm samples from two informed donors (Sperm 1 and Sperm 2), as well as blood sample from the donor of Sperm 1 (yielding somatic Genome 1) were collected and extracted using Winard DNA purification system (Promega). Sperm 1 showed heterozygous genotypes at rs1816071 (S3), rs1816072 (S5) and rs13178374 (S15), while Sperm 2 showed heterozygous genotypes at rs6891988 (S2), rs35351365 (S4) and rs1816072 (S5). All procedures other than the cell lysis step for the sperm samples followed the instructions of the purification system manual. Nuclei were washed with 1xPBS and pelleted by centrifugation at 13,000 g for 5 min. The pellets obtained were lysed at 55°C for 3 hours in 300 µl dithiothreitol (DTT)/proteinase K buffer containing 10 mM Tris-Cl (USB, Cleveland, Ohio), 10 mM NaCl (USB), 20 mM ethylene-diamine-tetraacetic acid (EDTA) (Invitrogen Corporation, Grand Island, NY), 1% SDS, 0.04% proteinase K and 1% DTT.

Amplification of a 760-bp DNA fragment that included SNPs S1 to S5 was performed in a 20 µl PCR mixture containing 100 ng sperm or genomic DNA, 75 nM of each of primers B2I8-214F and B2I8-214R (Table S8), 50 nM of each dNTP, 2.5 mM MgCl_2_ and 1 U Taq DNA polymerase (Amersham). Stringent PCR conditions using increased annealing temperatures and a minimum number of reaction cycles were employed to prevent PCR jumping. Reaction cycling consisted of initial denaturation at 94°C for 3 min, followed by 25 cycles of 30 sec at 95°C, 30 sec at 65°C, 50 sec at 72°C, and a final extension step at 72°C for 50 sec. PCR products were resolved on 1.2% agarose gels, and stained with 0.5 µg/ml ethidium bromide to confirm the presence of PCR products with the expected sizes and absence of non-specific products. PCR products were recovered using Gel DNA Purification Kit (Omega Bio-Tek, Doraville, GA).

Purified PCR products were cloned into pMD18-T vector (TaKaRa Co., Japan) by incubation of a 10 µl ligation reaction mixture containing 5.0 µl of purified PCR product, 5.0 µl ligation mix and 0.3 µl pMD18-T vector at 16°C overnight. Ligation reaction products were subsequently transformed into competent *Escherichia coli*: the 10 µl ligation reaction products were incubated with 100 µl competent cells on ice for 30 min, followed by heating at 42°C for 90 s and immediate cooling on ice for 2 min. A volume of 900 µl 1X Luria Broth (LB) (USB, Cleveland, Ohio) was added to the 100 µl transformed cells and incubated at 37°C for 1 hour. The cells were then spread onto LB agar plates containing 100 µg/ml ampicillin and incubated at 37°C overnight. Each colony on the plate was streaked on a new ampicillin-containing LB-plate. One single colony from each streaked plate was sub-cultured in 5 ml LB containing 100 µg/ml ampicillin. Plasmids were extracted from the cultured cells using an EZNA Plasmid Miniprep Kit (Omega Bio-Tek Co., Canada). The allelic forms of SNPs in each clone were determined by resequencing as described in the previous section.

The feasibility of the cloning procedures for typing sperm haplotypes was evaluated using the DNA of somatic Genome 1. As to be expected for somatic DNA, analysis of Genome 1 DNA yielded no detectable recombinants, confirming that the cloning method for recombinant detection was reliable. Applying this method to each of the sperm samples, the two major haplotype forms observed were taken to represent the two non-recombinant somatic forms. The two minor haplotype forms were taken to represent recombinant forms generated by meiotic cross-over between the two somatic forms. In each instance, the genetic distance between the two crossed-over SNPs was calculated based on the frequency of the more abundant of the two minor forms. Comparison between the genetic distance and the physical base-pair distance gave the rate of recombination.

### Haplotyping of Somatic DNA

To detect historical recombination events in the somatic DNA, haplotyping was performed on genomic DNA from the JP and GE control groups. Sixteen JP controls and nineteen GE controls were selected based on the criterion that each selected sample was heterozygous with respect to at least two of S1, S3, S5 and S24. The selected genomic DNA samples were PCR amplified to yield Fragment A, and cloned as described for the sperm samples. Eight single colonies of each sample were picked and re-cultured on a new LB plate; three of these single colony clones were sequenced to determine the allelic forms of S1, S3, S5 and S24, and the other five served as back-ups. Sequencing of the three clones revealed at least one or both of the two somatic haplotype forms. If only one of the two haplotype forms was revealed, the remaining haplotype form was deduced from the revealed haplotype form and the allelic genotypes of the four SNPs determined for the DNA sample. Besides experimental haplotyping based on cloning, haplotypes were inferred from the SNP genotypes of individual subjects using PHASE version 2.1 program as described [40].

### Detection of Recombination in Sperm DNA by Allele-Specific Real-Time PCR

To estimate the number of recombination events between S3, S5 and S15, nested allele-specific real-time PCR was performed with Sperm DNA 1 as described [41]. Purified DNAs were quantified using SYBR GreenER qPCR SuperMix Universal (Invitrogen) with the primers B2I8-ASPA-F1 and B2I8-ASPA-R1 (Table S6), detected using the MX3000P Real-time PCR system (Stratagene), and diluted to contain approximately 100 amplifiable genomes per µl.

The somatic S3-S5-S15 haplotypes of the Sperm 1 donor were determined to be G-C-C and A-T-G. Accordingly, allele-specific PCR (AS-PCR) was carried out to capture G-N-G recombinant combinations of S3–S4–S5 containing allele G at both S3 and S15. A total of 77 AS-PCR runs were performed. Each AS-PCR run was carried out with approximately 100 amplifiable sperm DNA genomes, 0.15 µM of allele-specific primers AS1816071GF1 and AS13178374GR1 (as shown in Table S8, both of comprising four phosphorothioate bonds at the 3′ end), 25 nM of each dNTP, 1xPCR buffer and 1 U of Taq polymerase (Amersham) in 20 µl. The AS-PCR procedure consisted of an initial polymerase activation at 94°C for 5 min, 25 cycles each of 30 sec at 95°C, 30 sec at 58°C and 5 min, and 40 sec at 72°C, and a final extension step at 72°C for 30 sec. For the nested allele-specific real-time PCR (AS-RT-PCR), a 0.5 µl aliquot of each of the first-stage AS-PCR products was added to the second-stage AS-RT-PCR.

To determine the frequencies of the G-T-G and G-C-G containing AS-PCR products by AS-RT-PCR, the reaction mixture contained 0.4 µM of the allele-specific forward primer AS1816072TF1 or AS1816072CF1, respectively, and the reverse primer B2I8-ASPA-R1, and 1 µM of Taqman MBG probe the fluorescent 6FAM-labelled B2I8-ASPA-T1 (Table S8). Each mixture also contained 0.5 µl of a first-stage AS-PCR product, 501nM of each dNTP, 1x PCR buffer (1.5 mM of MgCl_2_, 50 mM of KCl and 10 mM of Tris-Cl), 0.02 µM of reference dye ROX (6-carboxy-X-rhodamine) (Invitrogen) and 1 U of *Taq* DNA polymerase (Amersham Bioscience, Uppsala, Sweden) in a final volume of 10 µl. Amplification was carried out using the MX3000P Real-Time PCR System (Stratagene) with an initial denaturation at 95°C for 5 min, followed by 45 cycles each of 30 sec at 95°C, 45 sec at 60°C, 30 sec at 72°C, plus a dissociation step at 95°C for 11min followed by 30 sec of 55°C.

### DNase I Sensitivity Assay

In this assay, PCR fragment was generated with a mixture containing 50 ng of genomic DNA, 75 nM of each primer, 50 nM of each dNTP, 1X PCR buffer (1.5 mM of MgCl_2_, 50 mM of KCl and 10 mM of Tris-Cl), and 1 U of *Taq* DNA polymerase (Amersham Bioscience, Uppsala, Sweden) in a final volume of 20 µl. The forward primer was fluorescence-labeled 5′-6FAM-GATTCCTGCTTCTCTGTT-3′, and the reverse primer was B2I9-R4N in Table S8. The PCR reaction consisted of initial denaturation at 94°C for 2 min, followed by 30 cycles each of 30 sec at 95°C, 30 sec at 58°C, 90 sec at 72°C, plus a final extension step at 72°C for 51min. The PCR product was purified by ethanol precipitation and quantified at OD_260_. DNase I digestion was carried out by incubating ∼300 ng of PCR product, 10 mM MgCl_2_, 5 mM CaCl_2_, and 2.6 U DNase I at 23°C. Reaction was terminated at 30 sec, 1 min or 5 min by addition of 100 µl Stop Buffer (20 mM pH 8.0 EDTA, 1% SDS, 0.2 M NaCl). The digested PCR product was purified by ethanol precipitation, dissolved in 9.5 µl Hi-Deionized Formamide and 0.5 µl ROX-labelled DNA standard, denatured at 95°C for 1 min and immediately held at 4°C. The denatured PCR product was read with a Model 3100 Genetic Analyzer using the Gene Scan program (Applied Biosystems Inc., Foster City, California).

### Estimation of Population Recombination Rate

Population recombination rate (ρ) was estimated using the “hotspot” program in the coalescence-based software package Hotspotter version 1.2.1 [15], [42] with the inferred haplotype data generated by PHASE version 2.1 as input. The estimated population recombination rate, “rhohat”, output from the program for each bin of 350 bp, was plotted out giving rise to a profile of the S1–S29 segment. The “rhobar” output from Hotspotter was used to indicate average population recombination rate of the whole segment (3.5 kb) for each cohort. The population recombination rate (ρ) could be converted to recombination rate (r) by ρ = 4N_e_r where effective population size N_e_ = 10,000 [17].

### Statistical Differences in Linkage Equilibrium Patterns

Values of the LD coefficient *r* ^2^ were computed using DnaSP [43]. LDcontrast [44] was employed to compare the LD patterns of two groups. For each comparison, the SNPs within all possible regions flanked by any two SNPs were exhaustively evaluated using the “corr” option of LDcontrast. For each region, 10,000 simulations were performed to give the *P*-value.

### Construction of Haplotype Network

The hypothetical human ancestral sequence (ANC) was derived based on the comparison between human and chimpanzee genome sequences as described by Lo *et al*. [11] The genetic relationships between the inferred haplotypes were generated by NETWORK version 4.5 [45], using the phylogenetic median-joining network algorithm.

### Sequence Diversity Analysis

Based on the inferred haplotype data, DnaSP was employed to estimate haplotype diversity *H_d_* [18], Watterson's *θ* [19] and nucleotide diversity *π* [46].

## Supporting Information

Figure S1 Recombinations in HapMap populations. (A) Recombination hotspots in GABRB2. Recombination rates were determined for GABRB2 with genotype data from the HapMap based on the UCSC genome browser (http://genome.ucsc.edu/). Sequences of mRNAs of GABRB2, NM_000813 for the short isoform and NM_021911 for the long isoform, were aligned to human chromosome 5. Chromosome position covered by the gene is shown by the horizontal line, with arrows indicating gene orientation, extending from exons E1 to E11. Locations of E1 to E11 are indicated by the vertical black lines. The region indicated to be recombination hotspot regions on the browser are shown by green boxes; the recombination rates calculated from HapMap data from the browser are shown by black columns; and the 3,551-bp segment is shown by light red column. (B) Recombination profile of the full-length GABRB2 gene. Genotype data on SNPs in the GABRB2 analyzed in Phase-II of the HapMap project for four ethnic groups (YRI, CEU, JPT and CHB) were obtained from the HapMap database, which covered, among the SNPs in the S1-S29 segment, S1, S2, S3, S5, S7, S20, S28 and S29. Haplotype phases were inferred from genotype data by the PHASE program version 2.1, and recombination rates calculated across the 254-Kb GABRB2 using LDhat. The region of focus in this study and the peak rate within this region are highlighted in red for each population.
(1.44 MB DOC)

Figure S2 Population genetic parameters estimated for male (M) and female (F) AF non-schizophrenia cohorts. (A) Population recombination rates estimated by Hotspotter. The profile of the population recombination rate (rhohat) is shown in dark blue, and the estimated average population recombination rate (rhobar) in light blue. The number of chromosomes analyzed is indicated by the number inside parentheses following cohort identification. (B) Haplotype Diversity (Hd) and the mutation rate expressed by Watterson's Theta. Recombination strength measured by Hd/Theta for four SNP-containing sub-segments in S1–S29 sequence distinguished according to their positions relative to Alu Yi6AH-151 and Exon 9: Pre-Alu (S1–S5), Alu (S6–S19), Post-Alu (S20–S24) and Post-Exon (exonic synonymous S25 plus post-exonic S26–S29). (C) LD plots. Haplotype forms inferred from genotype data of the AF samples using PHASE version 2.1. One thousand resampling datasets were generated for the haplotypes of each of the M and F cohorts. Different haplotype forms are shown in Table S2. Coefficients of LD (r∧2) estimated by DnaSP for each SNP pair (Table S5) are displayed in thermal scale (shown at bottom of figure) for M (upper triangle) and F (lower triangle). For the M cohort, LD plots of the derived (D) and the ancestral (N) haplotype groups were also displayed in the same thermal scale, with D- and N-haplotypes being distinguished based on the allelic form of S5 contained in the haplotype (Table S6).
(2.77 MB DOC)

Table S1 Sequence diversity for the 3,551-bp GABRB2 segment.
(0.10 MB DOC)

Table S2 Haplotype-form compositions. Derived allelic state of each SNP is shown in red, and ancestral state is shown in light blue.
(0.41 MB DOC)

Table S3 (0.30 MB DOC)

Table S4 Detection of recombination between two target SNPs (SNP X and SNP Y) by cloning and allele-specific real-time PCR.
(0.06 MB DOC)

Table S5 Pairwise SNP linkage disequilibrium (LD) r∧2 values.
(0.72 MB DOC)

Table S6 Pairwise SNP linkage disequilibrium (LD) r∧2 values for N and D groups.
(0.64 MB DOC)

Table S7 P-value for gender differences and disease association in terms of LD and haplotype frequencies.
(1.08 MB DOC)

Table S8 Primers employed in the present study.
(0.06 MB DOC)

## References

[1] Gottesman II (1989). Vital statistics, demography, and schizophrenia: editor's introduction.. Schizophr Bull.

[2] Lupski JR (2008). Schizophrenia: Incriminating genomic evidence.. Nature.

[3] Sawa A, Snyder SH (2002). Schizophrenia: diverse approaches to a complex disease.. Science.

[4] Sherrington R, Brynjolfsson J, Petursson H, Potter M, Dudleston K (1988). Localization of a susceptibility locus for schizophrenia on chromosome 5.. Nature.

[5] Lo WS, Harano M, Gawlik M, Yu Z, Chen J (2007). GABRB2 association with schizophrenia: commonalities and differences between ethnic groups and clinical subtypes.. Biol Psychiatry.

[6] Lo WS, Lau CF, Xuan Z, Chan CF, Feng GY (2004). Association of SNPs and haplotypes in GABAA receptor beta2 gene with schizophrenia.. Mol Psychiatry.

[7] Zhao C, Xu Z, Chen J, Yu Z, Tong KL (2006). Two isoforms of GABA(A) receptor beta2 subunit with different electrophysiological properties: Differential expression and genotypical correlations in schizophrenia.. Mol Psychiatry.

[8] Liu J, Shi Y, Tang W, Guo T, Li D (2005). Positive association of the human GABA-A-receptor beta 2 subunit gene haplotype with schizophrenia in the Chinese Han population.. Biochem Biophys Res Commun.

[9] Petryshen TL, Middleton FA, Tahl AR, Rockwell GN, Purcell S (2005). Genetic investigation of chromosome 5q GABAA receptor subunit genes in schizophrenia.. Mol Psychiatry.

[10] Yu Z, Chen J, Shi H, Stoeber G, Tsang SY (2006). Analysis of GABRB2 association with schizophrenia in German population with DNA sequencing and one-label extension method for SNP genotyping.. Clin Biochem.

[11] Lo WS, Xu Z, Yu Z, Pun FW, Ng SK (2007). Positive selection within the Schizophrenia-associated GABA(A) receptor beta2 gene.. PLoS ONE.

[12] Crespi B, Summers K, Dorus S (2007). Adaptive evolution of genes underlying schizophrenia.. Proc Biol Sci.

[13] Batzer MA, Deininger PL (2002). Alu repeats and human genomic diversity.. Nat Rev Genet.

[14] Ng SK, Xue H (2006). Alu-associated enhancement of single nucleotide polymorphisms in the human genome.. Gene.

[15] Li N, Stephens M (2003). Modeling linkage disequilibrium and identifying recombination hotspots using single-nucleotide polymorphism data.. Genetics.

[16] de Massy B (2003). Distribution of meiotic recombination sites.. Trends Genet.

[17] McVean GA, Myers SR, Hunt S, Deloukas P, Bentley DR (2004). The fine-scale structure of recombination rate variation in the human genome.. Science.

[18] Nei M, Tajima F (1983). Maximum likelihood estimation of the number of nucleotide substitutions from restriction sites data.. Genetics.

[19] Watterson GA (1975). On the number of segregating sites in genetical models without recombination.. Theor Popul Biol.

[20] Jinks-Robertson S, Michelitch M, Ramcharan S (1993). Substrate length requirements for efficient mitotic recombination in Saccharomyces cerevisiae.. Mol Cell Biol.

[21] Jeffreys AJ, Neumann R, Panayi M, Myers S, Donnelly P (2005). Human recombination hot spots hidden in regions of strong marker association.. Nat Genet.

[22] Schizophrenia Research Forum.

[23] McGuffin P, Tandon K, Corsico A (2003). Linkage and association studies of schizophrenia.. Curr Psychiatry Rep.

[24] Ng MY, Levinson DF, Faraone SV, Suarez BK, DeLisi LE (2009). Meta-analysis of 32 genome-wide linkage studies of schizophrenia.. Mol Psychiatry.

[25] Purcell SM, Wray NR, Stone JL, Visscher PM, O'Donovan MC (2009). Common polygenic variation contributes to risk of schizophrenia and bipolar disorder.. Nature.

[26] Stefansson H, Ophoff RA, Steinberg S, Andreassen OA, Cichon S (2009). Common variants conferring risk of schizophrenia.. Nature.

[27] Berchowitz LE, Hanlon SE, Lieb JD, Copenhaver GP (2009). A positive but complex association between meiotic double-strand break hotspots and open chromatin in Saccharomyces cerevisiae.. Genome Res.

[28] Myers S, Spencer CC, Auton A, Bottolo L, Freeman C (2006). The distribution and causes of meiotic recombination in the human genome.. Biochem Soc Trans.

[29] Otto SP, Barton NH (1997). The evolution of recombination: removing the limits to natural selection.. Genetics.

[30] Freudenberg J, Fu YH, Ptacek LJ (2007). Enrichment of HapMap recombination hotspot predictions around human nervous system genes: evidence for positive selection?. Eur J Hum Genet.

[31] Freudenberg J, Fu YH, Ptacek LJ (2007). Human recombination rates are increased around accelerated conserved regions–evidence for continued selection?. Bioinformatics.

[32] Ptak SE, Roeder AD, Stephens M, Gilad Y, Paabo S (2004). Absence of the TAP2 human recombination hotspot in chimpanzees.. PLoS Biol.

[33] Winckler W, Myers SR, Richter DJ, Onofrio RC, McDonald GJ (2005). Comparison of fine-scale recombination rates in humans and chimpanzees.. Science.

[34] Crow TJ (2000). Schizophrenia as the price that homo sapiens pays for language: a resolution of the central paradox in the origin of the species.. Brain Res Brain Res Rev.

[35] Leonhard K (1999). Classification of Endogenous Psychoses and their Differentiated Etiology: Springer, New York.

[36] (1994). American Psychiatric Association Diagnostic and Statistical Manual of Mental Disorder: American Psychiatric Press, Washington, DC.

[37] Chowdari KV, Mirnics K, Semwal P, Wood J, Lawrence E (2002). Association and linkage analyses of RGS4 polymorphisms in schizophrenia.. Hum Mol Genet.

[38] Nurnberger JI, Blehar MC, Kaufmann CA, York-Cooler C, Simpson SG (1994). Diagnostic interview for genetic studies..

[39] Nickerson DA, Tobe VO, Taylor SL (1997). PolyPhred: automating the detection and genotyping of single nucleotide substitutions using fluorescence-based resequencing.. Nucleic Acids Res.

[40] Stephens M, Smith NJ, Donnelly P (2001). A new statistical method for haplotype reconstruction from population data.. Am J Hum Genet.

[41] Tiemann-Boege I, Calabrese P, Cochran DM, Sokol R, Arnheim N (2006). High-resolution recombination patterns in a region of human chromosome 21 measured by sperm typing.. PLoS Genet.

[42] Zhang Y, Niu T, Lin S, Zhao H (2009). Haplotype Structure. Handbook on Analyzing Human Genetic Data.

[43] Rozas J, Sanchez-DelBarrio JC, Messeguer X, Rozas R (2003). DnaSP, DNA polymorphism analyses by the coalescent and other methods.. Bioinformatics.

[44] Zaykin DV, Meng Z, Ehm MG (2006). Contrasting linkage-disequilibrium patterns between cases and controls as a novel association-mapping method.. Am J Hum Genet.

[45] Bandelt HJ, Forster P, Rohl A (1999). Median-joining networks for inferring intraspecific phylogenies.. Mol Biol Evol.

[46] Nei M, Li WH (1979). Mathematical model for studying genetic variation in terms of restriction endonucleases.. Proc Natl Acad Sci U S A.

