# Supplementary material for: A Recombination Hotspot in a Schizophrenia-Associated Region of GABRB2
Source: PLoS One. 2010 Mar 8;5(3):e9547. doi: 10.1371/journal.pone.0009547 (PMC2833194; doi:10.1371/journal.pone.0009547)
Supplement: Figure S1 — Recombinations in HapMap populations. (A) Recombination hotspots in GABRB2. Recombination rates were determined for GABRB2 with genotype data from the HapMap based on the UCSC genome browser (http://genome.ucsc.edu/). Sequences of mRNAs of GABRB2, NM_000813 for the short isoform and NM_021911 for the long isoform, were aligned to human chromosome 5. Chromosome position covered by the gene is shown by the horizontal line, with arrows indicating gene orientation, extending from exons E1 to E11. Locations of E1 to E11 are indicated by the vertical black lines. The region indicated to be recombination hotspot regions on the browser are shown by green boxes; the recombination rates calculated from HapMap data from the browser are shown by black columns; and the 3,551-bp segment is shown by light red column. (B) Recombination profile of the full-length GABRB2 gene. Genotype data on SNPs in the GABRB2 analyzed in Phase-II of the HapMap project for four ethnic groups (YRI, CEU, JPT and CHB) were obtained from the HapMap database, which covered, among the SNPs in the S1-S29 segment, S1, S2, S3, S5, S7, S20, S28 and S29. Haplotype phases were inferred from genotype data by the PHASE program version 2.1, and recombination rates calculated across the 254-Kb GABRB2 using LDhat. The region of focus in this study and the peak rate within this region are highlighted in red for each population. (1.44 MB DOC) [file pone.0009547.s001.doc]

**Figure S1. Recombinations in HapMap populations.** (A) Recombination hotspots in *GABRB2*. Recombination rates were determined for *GABRB2* with genotype data from the HapMap based on the UCSC genome browser (<http://genome.ucsc.edu/>). Sequences of mRNAs of *GABRB2*, NM_000813 for the short isoform and NM_021911 for the long isoform, were aligned to human chromosome 5. Chromosome position covered by the gene is shown by the horizontal line, with arrows indicating gene orientation, extending from exons E1 to E11. Locations of E1 to E11 are indicated by the vertical black lines. The region indicated to be recombination hotspot regions on the browser are shown by green boxes; the recombination rates calculated from HapMap data from the browser are shown by black columns; and the 3,551-bp segment is shown by light red column. (B) Recombination profile of the full-length *GABRB2* gene. Genotype data on SNPs in the *GABRB2* analyzed in Phase-II of the HapMap project for four ethnic groups (YRI, CEU, JPT and CHB) were obtained from the HapMap database, which covered, among the SNPs in the S1-S29 segment, S1, S2, S3, S5, S7, S20, S28 and S29. Haplotype phases were inferred from genotype data by the PHASE program version 2.1, and recombination rates calculated across the 254-Kb *GABRB2* using LDhat. The region of focus in this study and the peak rate within this region are highlighted in red for each population.


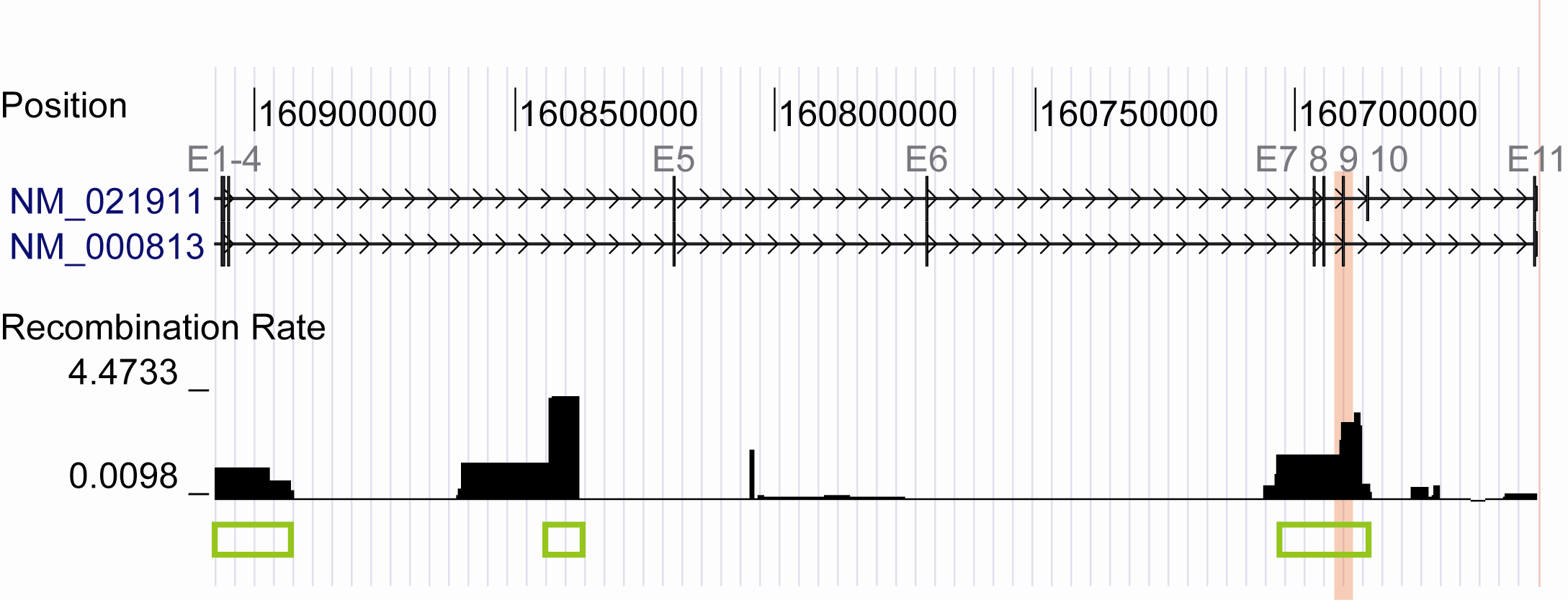


**Figure S1A**


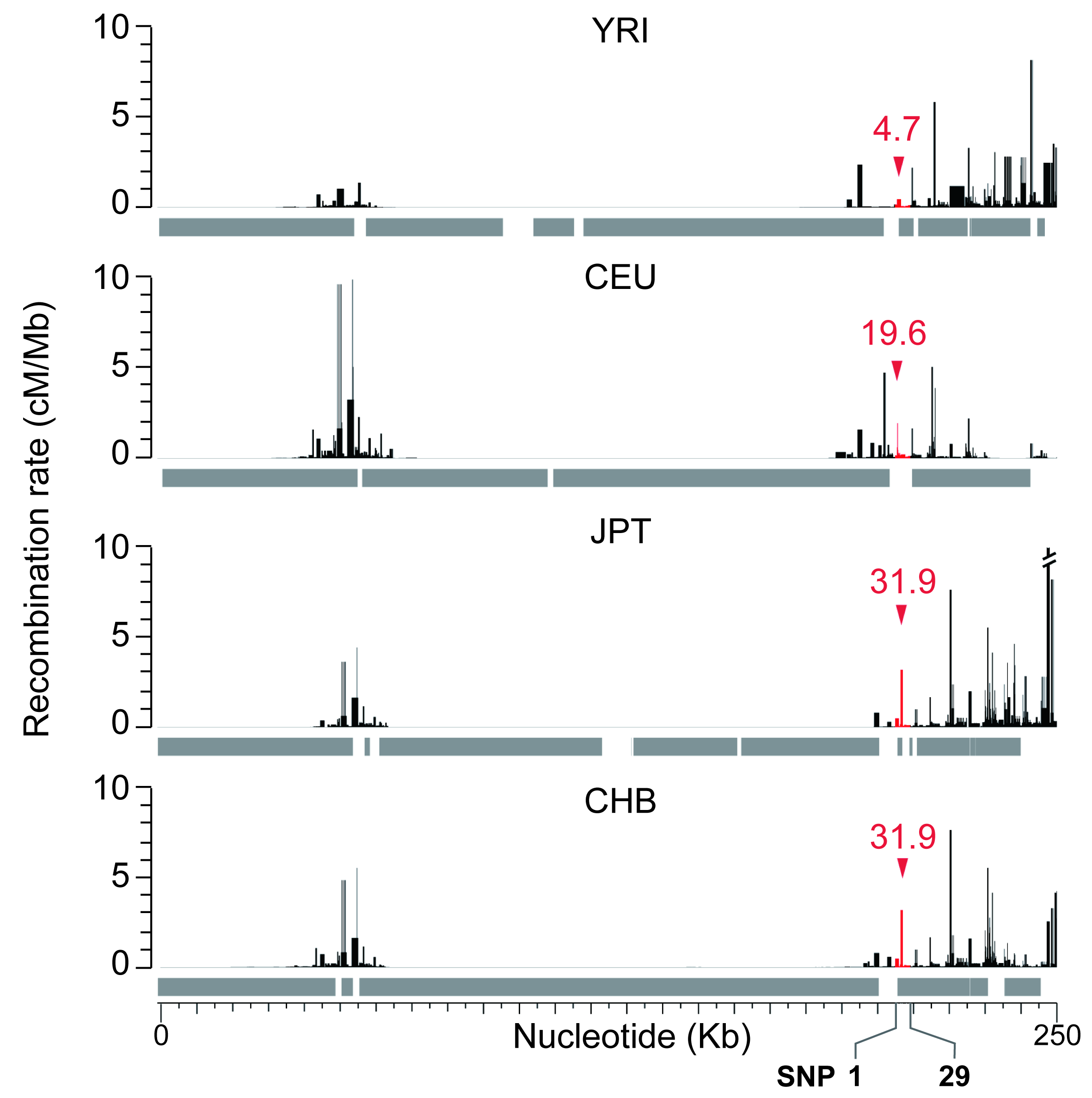


**Figure S1B**
