# Supplementary material for: A Recombination Hotspot in a Schizophrenia-Associated Region of GABRB2
Source: PLoS One. 2010 Mar 8;5(3):e9547. doi: 10.1371/journal.pone.0009547 (PMC2833194; doi:10.1371/journal.pone.0009547)
Supplement: Figure S2 — Population genetic parameters estimated for male (M) and female (F) AF non-schizophrenia cohorts. (A) Population recombination rates estimated by Hotspotter. The profile of the population recombination rate (rhohat) is shown in dark blue, and the estimated average population recombination rate (rhobar) in light blue. The number of chromosomes analyzed is indicated by the number inside parentheses following cohort identification. (B) Haplotype Diversity (Hd) and the mutation rate expressed by Watterson's Theta. Recombination strength measured by Hd/Theta for four SNP-containing sub-segments in S1–S29 sequence distinguished according to their positions relative to Alu Yi6AH-151 and Exon 9: Pre-Alu (S1–S5), Alu (S6–S19), Post-Alu (S20–S24) and Post-Exon (exonic synonymous S25 plus post-exonic S26–S29). (C) LD plots. Haplotype forms inferred from genotype data of the AF samples using PHASE version 2.1. One thousand resampling datasets were generated for the haplotypes of each of the M and F cohorts. Different haplotype forms are shown in Table S2. Coefficients of LD (r∧2) estimated by DnaSP for each SNP pair (Table S5) are displayed in thermal scale (shown at bottom of figure) for M (upper triangle) and F (lower triangle). For the M cohort, LD plots of the derived (D) and the ancestral (N) haplotype groups were also displayed in the same thermal scale, with D- and N-haplotypes being distinguished based on the allelic form of S5 contained in the haplotype (Table S6). (2.77 MB DOC) [file pone.0009547.s002.doc]

**Figure S2. Population genetic parameters estimated for male (M) and female (F) AF non-schizophrenia cohorts.**

(**A**) Population recombination rates estimated by Hotspotter.

The profile of the population recombination rate (“rhohat”) is shown in dark blue, and the estimated average population recombination rate (“rhobar”) in light blue. The number of chromosomes analyzed is indicated by the number inside parentheses following cohort identification.

(**B**) Haplotype Diversity (H_d_­) and the mutation rate expressed by Watterson’s Theta (θ).

Recombination strength measured by H_d_/θ for four SNP-containing sub-segments in S1-S29 sequence distinguished according to their positions relative to Alu Yi6AH-151 and Exon 9: Pre-Alu (S1-S5), Alu (S6-S19), Post-Alu (S20-S24) and Post-Exon (exonic synonymous S25 plus post-exonic S26-S29).

(**C**) LD plots.

Haplotype forms inferred from genotype data of the AF samples using PHASE version 2.1. One thousand resampling datasets were generated for the haplotypes of each of the M and F cohorts. Different haplotype forms are shown in Supplementary Table S2. Coefficients of LD (*r*^2^) estimated by DnaSP for each SNP pair (Table S5) are displayed in thermal scale (shown at bottom of figure) for M (upper triangle) and F (lower triangle). For the M cohort, LD plots of the derived (D) and the ancestral (N) haplotype groups were also displayed in the same thermal scale, with D- and N-haplotypes being distinguished based on the allelic form of S5 contained in the haplotype (Table S6).

**
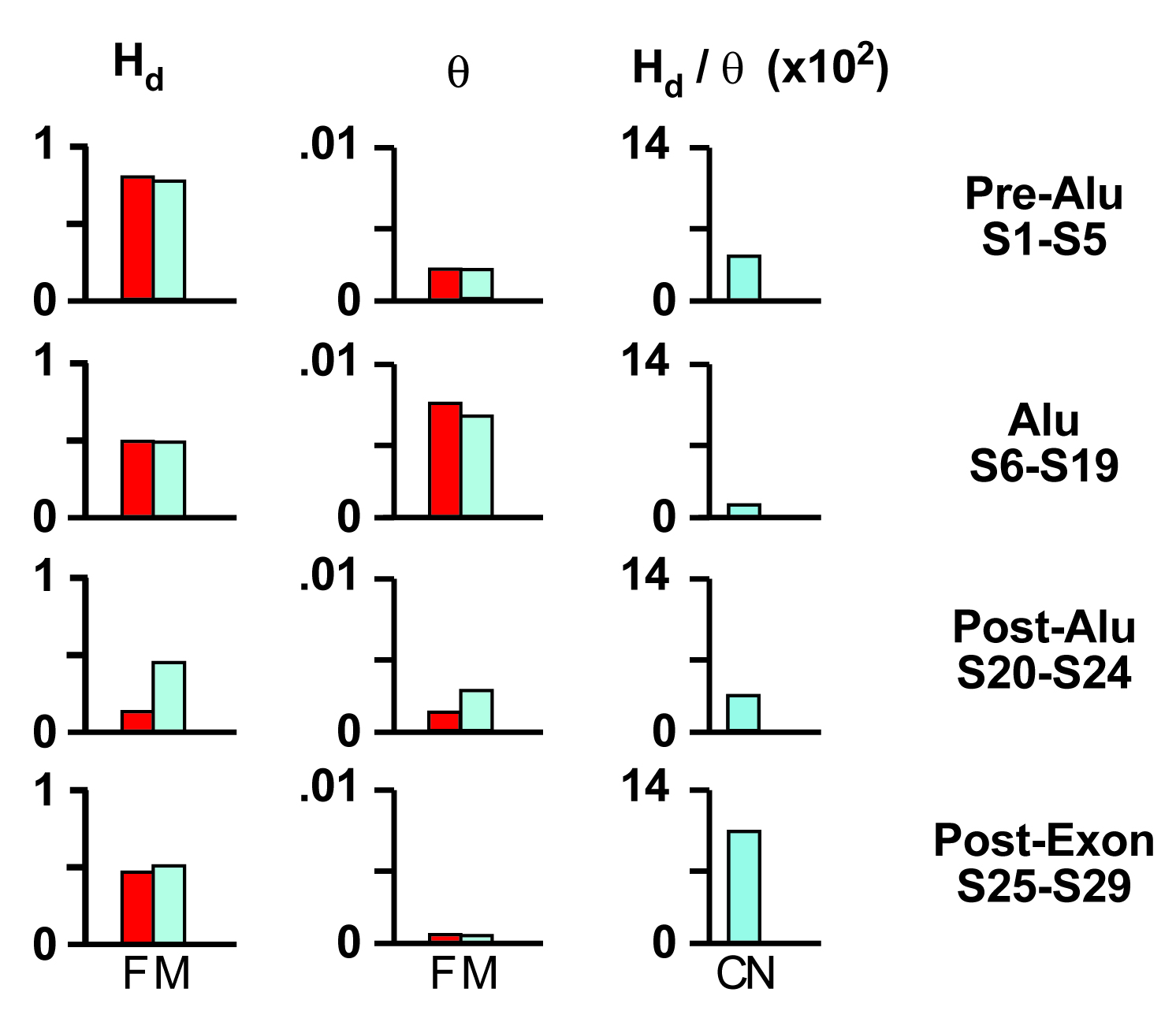
**

**Figure S2A**

**
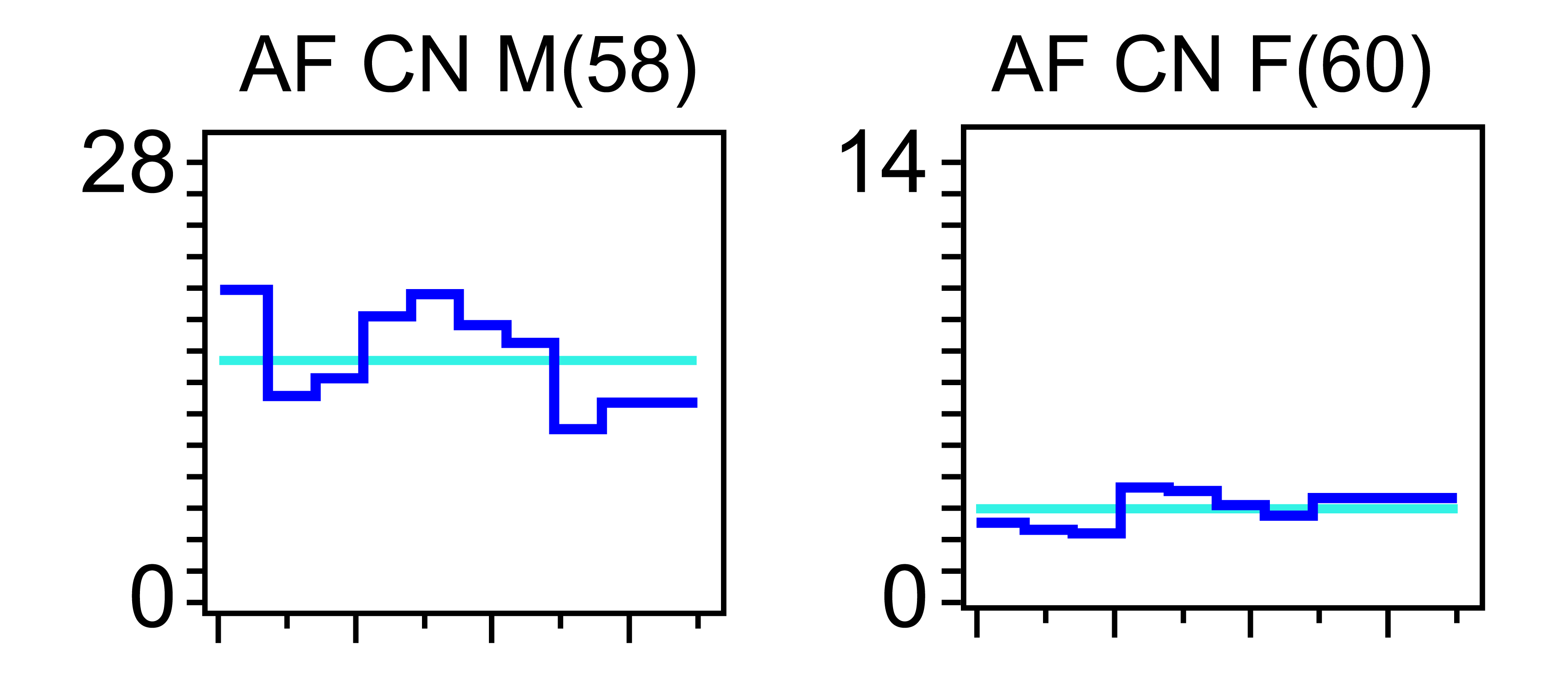
**

**Figure S2B**

**
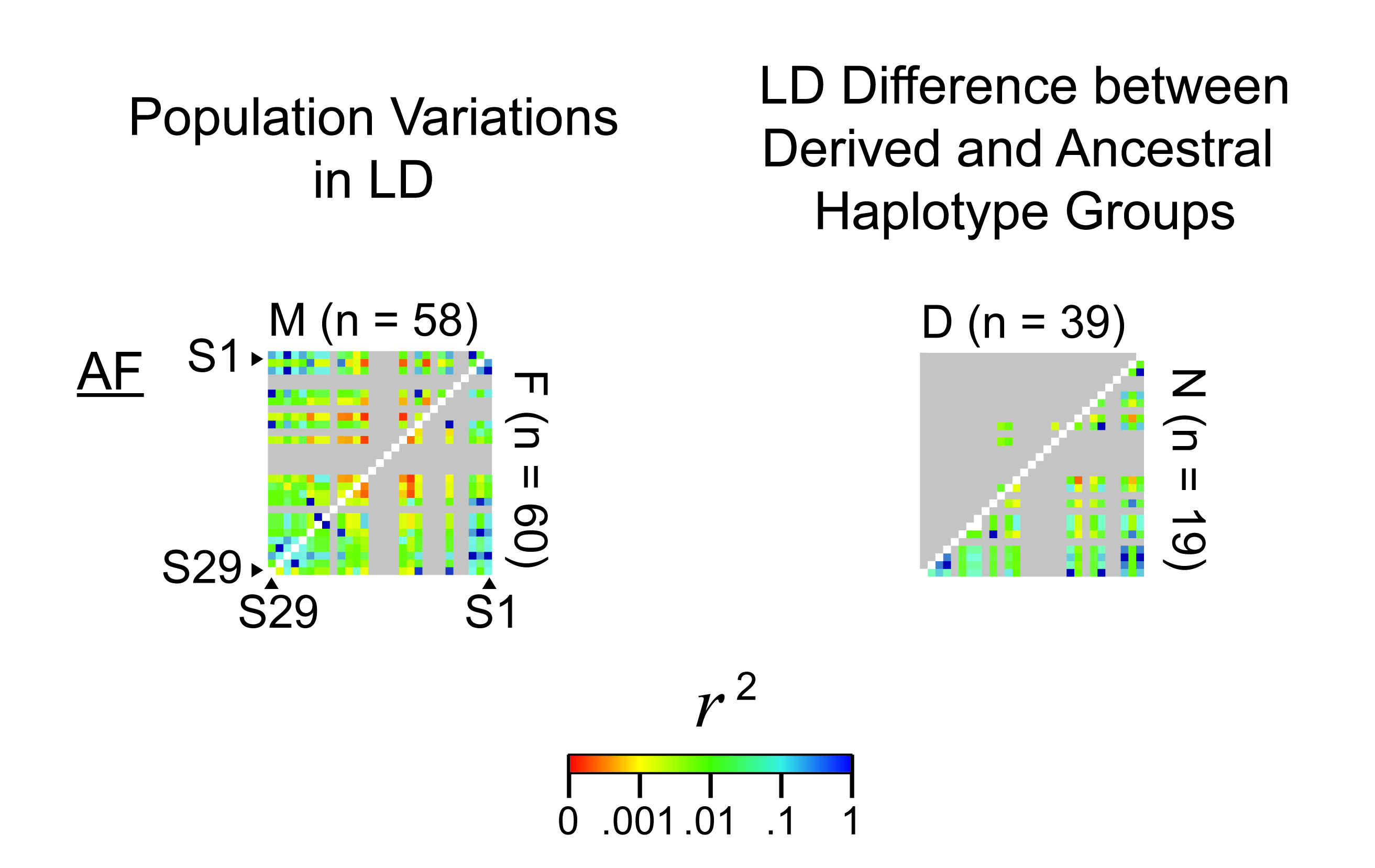
**

**Figure S2C**
