# Supplementary material for: A Recombination Hotspot in a Schizophrenia-Associated Region of GABRB2
Source: PLoS One. 2010 Mar 8;5(3):e9547. doi: 10.1371/journal.pone.0009547 (PMC2833194; doi:10.1371/journal.pone.0009547)
Supplement: Table S1 — Sequence diversity for the 3,551-bp GABRB2 segment. (0.10 MB DOC) [file pone.0009547.s003.doc]

**Table S1** Sequence diversity for the 3,551-bp *GABRB2* segment

| Parameters | AF | | GE | | | | US | | | | JP | | | | | Avg^a^ | |  |
| --- | --- | --- | --- | --- | --- | --- | --- | --- | --- | --- | --- | --- | --- | --- | --- | --- | --- | --- |
|  |  |  | CN | | SZ | | CN | | SZ | | CN | | SZ | | |  |  |  |
|  | F | M | F | M | F | M | F | M | F | M | F | M | | F | M | |  | |
| Number of chromosomes, *N* | 58 | 60 | 152 | 228 | 66 | 286 | 252 | 262 | 88 | 212 | 200 | 190 | | 184 | 406 | |  | |
| Number of variable sites, *S* |  |  |  |  |  |  |  |  |  |  |  |  | |  |  | |  | |
| Whole region (3,551 bp) | 19 | 20 | 18 | 18 | 17 | 18 | 20 | 24 | 18 | 19 | 17 | 17 | | 17 | 15 | | 19 | |
| Pre-Alu (S1-S5, 520 bp) | 5 | 5 | 3 | 3 | 3 | 3 | 3 | 5 | 3 | 3 | 5 | 5 | | 5 | 5 | | 4 | |
| Alu (S6-S19, 261 bp) | 9 | 8 | 9 | 9 | 8 | 9 | 10 | 12 | 9 | 10 | 6 | 6 | | 6 | 4 | | 8 | |
| Post-Alu (S20-S24, 319 bp) | 2 | 4 | 3 | 3 | 3 | 3 | 4 | 3 | 3 | 3 | 2 | 2 | | 2 | 2 | | 3 | |
| Post-exon (S26-S29, 1,114 bp) | 3 | 3 | 3 | 3 | 3 | 3 | 3 | 4 | 3 | 3 | 3 | 3 | | 3 | 3 | | 3 | |
| Number of Haplotypes, *h* |  |  |  |  |  |  |  |  |  |  |  |  | |  |  | |  | |
| Whole region | 14 | 17 | 14 | 20 | 6 | 19 | 14 | 18 | 10 | 16 | 20 | 21 | | 26 | 26 | | 17 | |
| Pre-Alu | 6 | 6 | 4 | 5 | 3 | 3 | 3 | 5 | 4 | 4 | 9 | 7 | | 8 | 9 | | 6 | |
| Alu | 9 | 7 | 5 | 8 | 4 | 11 | 8 | 8 | 5 | 6 | 4 | 4 | | 4 | 3 | | 7 | |
| Post-Alu | 2 | 5 | 3 | 3 | 3 | 3 | 5 | 3 | 3 | 3 | 4 | 4 | | 3 | 3 | | 4 | |
| Post-exon | 3 | 3 | 6 | 5 | 3 | 5 | 4 | 6 | 3 | 5 | 4 | 6 | | 6 | 6 | | 5 | |
| Haplotype diversity, *H*_d_ |  |  |  |  |  |  |  |  |  |  |  |  | |  |  | |  | |
| Whole region | 0.86 | 0.89 | 0.68 | 0.66 | 0.30 | 0.60 | 0.53 | 0.59 | 0.52 | 0.58 | 0.67 | 0.65 | | 0.69 | 0.73 | | 0.69 | |
| Pre-Alu | 0.80 | 0.78 | 0.57 | 0.54 | 0.27 | 0.50 | 0.47 | 0.50 | 0.48 | 0.50 | 0.63 | 0.58 | | 0.62 | 0.68 | | 0.61 | |
| Alu | 0.50 | 0.49 | 0.53 | 0.50 | 0.25 | 0.50 | 0.43 | 0.46 | 0.37 | 0.47 | 0.17 | 0.21 | | 0.20 | 0.23 | | 0.41 | |
| Post-Alu | 0.13 | 0.46 | 0.33 | 0.29 | 0.12 | 0.27 | 0.24 | 0.26 | 0.22 | 0.26 | 0.35 | 0.25 | | 0.29 | 0.27 | | 0.46 | |
| Post-exon | 0.47 | 0.51 | 0.56 | 0.54 | 0.29 | 0.49 | 0.45 | 0.48 | 0.44 | 0.48 | 0.37 | 0.31 | | 0.40 | 0.40 | | 0.46 | |
| Watterson's theta,θ per site (%)^b^ |  |  |  |  |  |  |  |  |  |  |  |  | |  |  | |  | |
| Whole region | 0.12 | 0.12 | 0.09 | 0.08 | 0.10 | 0.08 | 0.09 | 0.11 | 0.10 | 0.09 | 0.08 | 0.08 | | 0.08 | 0.06 | | 0.10 | |
| Pre-Alu | 0.21 | 0.21 | 0.10 | 0.10 | 0.12 | 0.09 | 0.10 | 0.16 | 0.11 | 0.10 | 0.16 | 0.17 | | 0.17 | 0.15 | | 0.15 | |
| Alu | 0.74 | 0.66 | 0.62 | 0.57 | 0.64 | 0.55 | 0.63 | 0.75 | 0.68 | 0.65 | 0.39 | 0.40 | | 0.40 | 0.23 | | 0.59 | |
| Post-Alu | 0.13 | 0.27 | 0.17 | 0.16 | 0.20 | 0.15 | 0.21 | 0.15 | 0.19 | 0.16 | 0.11 | 0.11 | | 0.11 | 0.10 | | 0.16 | |
| Post-exon | 0.06 | 0.06 | 0.05 | 0.05 | 0.06 | 0.04 | 0.04 | 0.06 | 0.05 | 0.05 | 0.05 | 0.05 | | 0.05 | 0.04 | | 0.05 | |
| Nuclotide diversity,π per site (%) |  |  |  |  |  |  |  |  |  |  |  |  | |  |  | |  | |
| Whole region | 0.12 | 0.13 | 0.12 | 0.11 | 0.06 | 0.10 | 0.10 | 0.10 | 0.09 | 0.10 | 0.10 | 0.08 | | 0.09 | 0.10 | | 0.11 | |
| Pre-Alu | 0.33 | 0.38 | 0.22 | 0.21 | 0.11 | 0.20 | 0.19 | 0.21 | 0.19 | 0.19 | 0.32 | 0.28 | | 0.31 | 0.34 | | 0.27 | |
| Alu | 0.38 | 0.32 | 0.53 | 0.50 | 0.23 | 0.45 | 0.41 | 0.43 | 0.33 | 0.42 | 0.10 | 0.14 | | 0.12 | 0.17 | | 0.35 | |
| Post-Alu | 0.08 | 0.26 | 0.20 | 0.17 | 0.07 | 0.16 | 0.14 | 0.15 | 0.14 | 0.16 | 0.21 | 0.14 | | 0.18 | 0.17 | | 0.17 | |
| Post-exon | 0.10 | 0.09 | 0.10 | 0.10 | 0.06 | 0.09 | 0.09 | 0.09 | 0.08 | 0.09 | 0.07 | 0.06 | | 0.06 | 0.07 | | 0.09 | |

**^a^ Average values were calculated based on the control samples.**

**^b^ Average sequence diversity θ and π are both approximately 0.1% for the human genome.**
