# Supplementary material for: A Recombination Hotspot in a Schizophrenia-Associated Region of GABRB2
Source: PLoS One. 2010 Mar 8;5(3):e9547. doi: 10.1371/journal.pone.0009547 (PMC2833194; doi:10.1371/journal.pone.0009547)
Supplement: Table S2 — Haplotype-form compositions. Derived allelic state of each SNP is shown in red, and ancestral state is shown in light blue. (0.41 MB DOC) [file pone.0009547.s004.doc]

**Table S2** Haplotype-form compositions. Derived allelic state of each SNP is shown in red, and ancestral state is shown in light blue.

| Hap | |  | SNP | | | | | | | | | | | | | | | | | | | | | | | | | | | | |
| --- | --- | --- | --- | --- | --- | --- | --- | --- | --- | --- | --- | --- | --- | --- | --- | --- | --- | --- | --- | --- | --- | --- | --- | --- | --- | --- | --- | --- | --- | --- | --- |
|  |  |  | S1 | S2 | S3 | S4 | S5 | S6 | S7 | S8 | S9 | S10 | S11 | S12 | S13 | S14 | S15 | S16 | S17 | S18 | S19 | S20 | S21 | S22 | S23 | S24 | S25 | S26 | S27 | S28 | S29 |
|  |  | dbSNP ID^a^ | rs6556547 | rs6891988 | rs1816071 | rs35351365 | rs1816072 | rs10060148 | rs2617505 | rs2546620 | rs34820615 | rs10060079 | rs41298420 | rs41298418 | rs41298416 | rs34691510 | rs13178374 | rs35858335 | rs34941072 | rs41298414 | rs41298412 | rs194072 | rs41298410 | rs35595756 | rs41298408 | rs252944 | rs41298406 | rs41298404 | rs252943 | rs10052351 | rs187269 |
|  | Genbank  Acc. No.^c^ | Chr Pos^b^ | 160,692,753 | 160,692,586 | 160,692,534 | 160,692,463 | 160,692,234 | 160,691,058 | 160,691,225 | 160,691,233 | 160,691,462 | 160,691,476 | 160,691,479 | 160,691,521 | 160,691,546 | 160,691,582 | 160,691,598 | 160,691,609 | 160,691,611 | 160,691,624 | 160,691,683 | 160,691,705 | 160,691,706 | 160,691,722 | 160,690,984 | 160,690,485 | 160,690,915 | 160,690,164 | 160,690,316 | 160,690,040 | 160,689,203 |
| H1 | GU117807 |  | G | G | A | C | T | C | C | A | C | C | G | C | T | G | G | G | C | G | C | T | A | C | C | G | C | A | C | A | T |
| H2 | GU117804 |  | G | G | A | C | T | C | C | A | C | C | G | C | T | G | G | G | C | G | C | T | A | C | C | G | C | A | C | A | C |
| H3 | GU117813 |  | G | G | A | C | T | C | C | A | C | C | G | C | T | G | G | G | C | G | C | T | A | C | C | G | C | A | C | G | T |
| H4 | GU117803 |  | G | G | A | C | T | C | C | A | C | C | G | C | T | G | G | G | C | G | C | T | A | C | C | G | C | A | A | A | T |
| H5 | GU117788 |  | G | G | A | C | T | C | C | A | C | C | G | C | T | G | G | G | C | G | C | T | A | C | C | G | C | A | A | A | C |
| H6 | GU117786 |  | G | G | A | C | T | C | C | A | C | C | G | C | T | G | G | G | C | G | C | T | A | C | C | G | C | A | A | G | T |
| H7 | GU117787 |  | G | G | A | C | T | C | C | A | C | C | G | C | T | G | G | G | C | G | C | T | A | C | C | G | C | A | A | G | C |
| H8 | GU117808 |  | G | G | A | C | T | C | C | A | C | C | G | C | T | G | G | G | C | G | C | T | A | C | C | G | T | A | C | A | T |
| H9 | GU117782 |  | G | G | A | C | T | C | C | A | C | C | G | C | T | G | G | G | C | G | C | T | A | C | C | C | C | A | C | A | T |
| H10 | GU117784 |  | G | G | A | C | T | C | C | A | C | C | G | C | T | G | G | G | C | G | C | C | A | C | C | G | C | A | C | A | T |
| H11 | GU117785 |  | G | G | A | C | T | C | C | A | C | C | G | C | T | G | G | G | C | G | C | C | A | C | C | C | C | A | C | A | T |
| H12 | GU117805 |  | G | G | A | C | T | C | C | A | C | C | G | C | T | G | G | G | C | A | C | T | A | C | C | G | C | A | C | A | T |
| H13 | GU117779 |  | G | G | A | C | T | C | C | A | C | C | G | C | T | G | C | G | C | G | C | T | A | C | C | G | C | A | C | A | T |
| H14 | GU117809 |  | G | G | A | C | T | C | C | A | C | C | G | T | T | G | G | G | C | G | C | T | A | C | C | G | C | A | C | A | T |
| H15 | GU117806 |  | G | G | A | C | T | C | C | A | C | C | A | C | T | G | G | G | C | G | C | T | A | C | C | G | C | A | C | A | T |
| H16 | GU117810 |  | G | G | A | C | T | C | C | A | C | T | G | C | T | G | G | G | C | G | C | T | A | C | C | G | C | A | C | A | T |
| H17 | GU117828 |  | G | G | A | C | T | C | G | G | C | C | G | C | T | G | G | G | C | G | C | C | A | C | C | C | C | A | A | A | C |
| H18 | GU117811 |  | G | G | A | C | T | T | C | A | C | C | G | C | T | G | G | G | C | G | C | T | A | C | C | G | C | A | C | A | T |
| H19 | GU117823 |  | G | G | A | C | C | C | C | A | C | C | G | C | T | G | G | G | C | G | C | T | A | C | C | G | C | A | C | A | T |
| H20 | GU117802 |  | G | G | A | C | C | C | C | A | C | C | G | C | T | G | G | G | C | G | C | T | A | C | C | G | C | A | A | A | C |
| H21 | GU117783 |  | G | G | A | C | C | C | C | A | C | C | G | C | T | G | G | G | C | G | C | T | A | C | C | C | C | A | C | A | T |
| H22 | GU117781 |  | G | G | A | C | C | C | C | A | C | C | G | C | T | G | C | G | C | G | C | T | A | C | C | G | C | A | C | A | T |
| H23 | GU117822 |  | G | G | A | T | C | C | C | A | C | C | G | C | T | G | G | G | C | G | C | T | A | C | C | G | C | A | C | A | T |
| H24 | GU117824 |  | G | G | G | C | T | C | C | A | C | C | G | C | T | G | G | G | C | G | C | T | A | C | C | G | C | A | C | A | T |
| H25 | GU117780 |  | G | G | G | C | T | C | C | A | C | C | G | C | T | G | C | G | C | G | C | T | A | C | C | G | C | A | C | A | T |
| H26 | GU117851 |  | G | G | G | C | C | C | C | A | C | C | G | C | T | G | G | G | C | G | C | T | A | C | C | G | C | A | C | A | T |
| H27 | GU117838 |  | G | G | G | C | C | C | C | A | C | C | G | C | T | G | G | G | C | G | C | T | A | C | C | G | C | A | A | A | T |
| H28 | GU117789 |  | G | G | G | C | C | C | C | A | C | C | G | C | T | G | G | G | C | G | C | T | A | C | C | G | C | A | A | A | C |
| H29 | GU117801 |  | G | G | G | C | C | C | C | A | C | C | G | C | T | G | G | G | C | G | C | T | A | C | C | G | C | A | A | G | C |
| H30 | GU117790 |  | G | G | G | C | C | C | C | A | C | C | G | C | T | G | G | G | C | G | C | T | A | C | A | G | C | A | A | A | C |
| H31 | GU117791 |  | G | G | G | C | C | C | C | A | C | C | G | C | T | G | G | G | C | G | C | T | C | C | C | G | C | A | A | A | C |
| H32 | GU117852 |  | G | G | G | C | C | C | C | A | C | C | G | C | T | G | G | G | C | G | C | C | A | C | C | C | C | A | C | A | C |
| H33 | GU117853 |  | G | G | G | C | C | C | C | A | C | C | G | C | T | G | G | G | C | G | C | C | A | C | C | C | C | A | A | A | C |
| H34 | GU117840 |  | G | G | G | C | C | C | C | A | C | C | G | C | T | G | C | G | C | G | C | T | A | C | C | G | C | A | C | A | T |
| H35 | GU117848 |  | G | G | G | C | C | C | C | A | C | C | G | C | T | G | C | G | C | G | C | T | A | C | C | G | C | A | C | A | C |
| H36 | GU117839 |  | G | G | G | C | C | C | C | A | C | C | G | C | T | G | C | G | C | G | C | T | A | C | C | G | C | A | A | A | T |
| H37 | GU117841 |  | G | G | G | C | C | C | C | A | C | C | G | C | T | G | C | G | C | G | C | T | A | C | C | G | C | G | C | A | T |
| H38 | GU117842 |  | G | G | G | C | C | C | C | A | C | C | G | C | T | G | C | G | C | G | C | C | A | C | C | G | C | A | C | A | T |
| H39 | GU117843 |  | G | G | G | C | C | C | C | A | C | C | G | C | T | A | G | A | T | G | C | T | A | A | C | G | C | A | C | A | C |
| H40 | GU117844 |  | G | G | G | C | C | C | C | A | C | C | G | C | T | A | G | A | T | G | C | T | A | A | C | G | C | A | A | A | C |
| H41 | GU117847 |  | G | G | G | C | C | C | C | A | C | C | G | C | T | A | C | A | T | G | C | T | A | A | C | G | C | A | C | A | C |
| H42 | GU117845 |  | G | G | G | C | C | C | C | A | C | C | G | C | T | A | C | A | T | G | C | T | A | A | C | G | C | A | A | A | T |
| H43 | GU117846 |  | G | G | G | C | C | C | C | A | C | C | G | C | T | A | C | A | T | G | C | T | A | A | C | G | C | A | A | A | C |
| H44 | GU117796 |  | G | G | G | C | C | C | C | A | T | T | G | C | T | G | G | G | C | G | C | T | A | C | C | G | C | A | A | G | C |
| H45 | GU117836 |  | G | G | G | C | C | C | G | G | C | C | G | C | T | G | G | G | C | G | C | T | A | C | C | G | C | A | C | A | T |
| H46 | GU117830 |  | G | G | G | C | C | C | G | G | C | C | G | C | T | G | G | G | C | G | C | T | A | C | C | G | C | A | A | A | C |
| H47 | GU117829 |  | G | G | G | C | C | C | G | G | C | C | G | C | T | G | G | G | C | G | C | T | A | C | C | C | C | A | A | A | C |
| H48 | GU117833 |  | G | G | G | C | C | C | G | G | C | C | G | C | T | G | G | G | C | G | C | T | C | C | C | G | C | A | A | A | C |
| H49 | GU117827 |  | G | G | G | C | C | C | G | G | C | C | G | C | T | G | G | G | C | G | C | C | A | C | C | C | C | A | C | A | C |
| H50 | GU117825 |  | G | G | G | C | C | C | G | G | C | C | G | C | T | G | G | G | C | G | C | C | A | C | C | C | C | A | A | A | C |
| H51 | GU117837 |  | G | G | G | C | C | C | G | G | C | C | G | C | T | G | C | G | C | G | C | T | A | C | C | G | C | A | C | A | T |
| H52 | GU117835 |  | G | G | G | C | C | C | G | G | C | C | G | C | T | G | C | G | C | G | C | T | A | C | C | G | C | A | A | A | C |
| H53 | GU117832 |  | G | G | G | C | C | C | G | G | C | C | G | C | T | A | G | G | C | G | C | T | A | C | C | G | C | A | A | A | C |
| H54 | GU117831 |  | G | G | G | C | C | C | G | G | C | C | G | C | C | G | G | G | C | G | C | T | A | C | C | G | C | A | A | A | C |
| H55 | GU117834 |  | G | G | G | C | C | C | G | G | C | T | G | C | T | G | G | G | C | G | C | T | A | C | C | G | C | A | A | A | C |
| H56 | GU117800 |  | G | G | G | C | C | T | C | A | C | C | G | C | T | G | G | G | C | G | C | T | A | C | C | G | C | A | A | G | C |
| H57 | GU117799 |  | G | G | G | C | C | T | C | A | C | T | G | C | T | G | G | G | C | G | C | T | A | C | C | G | C | A | A | A | C |
| H58 | GU117797 |  | G | G | G | C | C | T | C | A | C | T | G | C | T | G | G | G | C | G | C | T | A | C | C | G | C | A | A | G | C |
| H59 | GU117798 |  | G | G | G | C | C | T | C | A | C | T | G | C | T | G | G | G | C | G | A | T | A | C | C | G | C | A | A | G | C |
| H60 | GU117849 |  | G | G | G | C | C | T | C | A | T | T | G | C | T | G | G | G | C | G | C | T | A | C | C | G | C | A | C | A | T |
| H61 | GU117850 |  | G | G | G | C | C | T | C | A | T | T | G | C | T | G | G | G | C | G | C | T | A | C | C | G | C | A | C | A | C |
| H62 | GU117795 |  | G | G | G | C | C | T | C | A | T | T | G | C | T | G | G | G | C | G | C | T | A | C | C | G | C | A | A | A | C |
| H63 | GU117793 |  | G | G | G | C | C | T | C | A | T | T | G | C | T | G | G | G | C | G | C | T | A | C | C | G | C | A | A | G | T |
| H64 | GU117794 |  | G | G | G | C | C | T | C | A | T | T | G | C | T | G | G | G | C | G | C | T | A | C | C | G | C | A | A | G | C |
| H65 | GU117821 |  | G | A | A | T | T | C | C | A | C | C | G | C | T | G | G | G | C | G | C | T | A | C | C | G | C | A | C | A | T |
| H66 | GU117814 |  | G | A | A | T | C | C | C | A | C | C | G | C | T | G | G | G | C | G | C | T | A | C | C | G | C | A | C | A | T |
| H67 | GU117819 |  | G | A | A | T | C | C | C | A | C | C | G | C | T | G | G | G | C | G | C | T | A | C | C | G | C | A | A | A | T |
| H68 | GU117820 |  | G | A | A | T | C | C | C | A | C | C | G | C | T | G | G | G | C | G | C | T | A | C | C | G | C | A | A | G | T |
| H69 | GU117815 |  | G | A | A | T | C | C | C | A | C | C | G | C | T | G | G | G | C | G | C | T | A | C | C | G | T | A | C | A | T |
| H70 | GU117817 |  | G | A | A | T | C | C | C | A | C | C | G | C | T | G | G | G | C | G | C | T | A | C | C | C | C | A | C | A | C |
| H71 | GU117818 |  | G | A | A | T | C | C | C | A | C | C | G | C | T | G | G | G | C | G | C | C | A | C | C | C | C | A | C | A | T |
| H72 | GU117812 |  | T | G | A | C | T | C | C | A | C | C | G | C | T | G | G | G | C | G | C | T | A | C | C | G | C | A | C | A | T |
| H73 | GU117854 |  | T | G | A | C | T | C | C | A | C | C | G | C | T | G | G | G | C | G | C | C | A | C | C | C | C | A | A | A | C |
| H74 | GU117864 |  | T | G | G | C | T | C | C | A | C | C | G | C | T | G | G | G | C | G | C | C | A | C | C | C | C | A | C | A | T |
| H75 | GU117855 |  | T | G | G | C | T | C | C | A | C | C | G | C | T | G | G | G | C | G | C | C | A | C | C | C | C | A | A | A | C |
| H76 | GU117865 |  | T | G | G | C | T | C | C | A | C | C | G | C | T | G | G | G | C | G | C | C | A | C | C | C | T | A | C | A | T |
| H77 | GU117868 |  | T | G | G | C | C | C | C | A | C | C | G | C | T | G | G | G | C | G | C | T | A | C | C | G | C | A | C | A | T |
| H78 | GU117792 |  | T | G | G | C | C | C | C | A | C | C | G | C | T | G | G | G | C | G | C | T | A | C | C | G | C | A | A | A | C |
| H79 | GU117863 |  | T | G | G | C | C | C | C | A | C | C | G | C | T | G | G | G | C | G | C | C | A | C | C | G | C | A | A | A | C |
| H80 | GU117866 |  | T | G | G | C | C | C | C | A | C | C | G | C | T | G | G | G | C | G | C | C | A | C | C | C | C | A | C | A | T |
| H81 | GU117862 |  | T | G | G | C | C | C | C | A | C | C | G | C | T | G | G | G | C | G | C | C | A | C | C | C | C | A | C | A | C |
| H82 | GU117867 |  | T | G | G | C | C | C | C | A | C | C | G | C | T | G | G | G | C | G | C | C | A | C | C | C | C | A | A | A | T |
| H83 | GU117856 |  | T | G | G | C | C | C | C | A | C | C | G | C | T | G | G | G | C | G | C | C | A | C | C | C | C | A | A | A | C |
| H84 | GU117858 |  | T | G | G | C | C | C | C | A | C | C | G | C | T | G | G | G | C | G | C | C | A | C | C | C | C | A | A | G | C |
| H85 | GU117861 |  | T | G | G | C | C | C | C | A | C | C | G | C | T | G | C | G | C | G | C | C | A | C | C | C | C | A | A | A | C |
| H86 | GU117857 |  | T | G | G | C | C | C | C | A | C | C | G | T | T | G | G | G | C | G | C | C | A | C | C | C | C | A | A | A | C |
| H87 | GU117826 |  | T | G | G | C | C | C | G | G | C | C | G | C | T | G | G | G | C | G | C | C | A | C | C | C | C | A | A | A | C |
| H88 | GU117816 |  | T | A | A | T | C | C | C | A | C | C | G | T | T | G | G | G | C | G | C | T | A | C | C | G | C | A | C | A | T |
| H89 | GU117859 |  | T | A | G | C | C | C | C | A | C | C | G | C | T | G | G | G | C | G | C | C | A | C | C | C | C | A | A | A | C |
| H90 | GU117860 |  | T | A | G | T | C | C | C | A | C | C | G | C | T | G | G | G | C | G | C | C | A | C | C | C | C | A | A | A | C |
| ANC^d^ |  |  | T | G | G | C | C | C | C | A | C | C | G | C | T | G | G | G | C | G | C | T | A | C | C | G | C | A | C | A | C |

^a^ dbSNP, SNP database http://www.ncbi.nlm.nih.gov/projects/SNP/

^b^ Based on chromosome 5 contig NT_023133.12. Derived alleles of SNPs are shown in red, and ancestral alleles are in light blue.

^c^ Genomic sequences containing each of haplotypes are available in the NCBI Genbank database.

^d^ Hypothetical ancestral haplotype.
