# Supplementary material for: A Recombination Hotspot in a Schizophrenia-Associated Region of GABRB2
Source: PLoS One. 2010 Mar 8;5(3):e9547. doi: 10.1371/journal.pone.0009547 (PMC2833194; doi:10.1371/journal.pone.0009547)
Supplement: Table S3 — (0.30 MB DOC) [file pone.0009547.s005.doc]

**Table S3**

**A**. Numbers of various inferred haplotype-forms in different population cohorts. Finite count of a haplotype form in a CN group is shaded blue when the corresponding SZ group displayed a zero count. Finite count of a haplotype form in an SZ group is shaded pink when the corresponding CN group displayed a zero count.

| **Hap#** | **AF** | | **GE** | | | | **US** | | | | **JP** | | | |
| --- | --- | --- | --- | --- | --- | --- | --- | --- | --- | --- | --- | --- | --- | --- |
|  |  |  | **CN** | | **SZ** | | **CN** | | **SZ** | | **CN** | | **SZ** | |
|  | **M** | **F** | **M** | **F** | **M** | **F** | **M** | **F** | **M** | **F** | **M** | **F** | **M** | **F** |
| Total | 58 | 60 | 228 | 152 | 286 | 66 | 262 | 252 | 212 | 88 | 190 | 200 | 406 | 184 |
| H1 | 12 | 11 | 129 | 80 | 175 | 55 | 164 | 170 | 134 | 60 | 109 | 108 | 196 | 98 |
| H2 | - | - | 4 | 1 | 1 | - | 1 | - | 1 | - | - | - | - | - |
| H3 | - | - | - | - | - | - | 1 | - | - | - | - | - | - | - |
| H4 | - | - | - | - | - | - | - | - | - | - | 1 | - | 1 | 5 |
| H5 | - | - | - | - | - | - | 1 | - | - | - | - | - | - | - |
| H6 | - | - | - | - | - | - | - | - | - | - | 1 | - | 1 | 1 |
| H7 | - | - | 1 | - | - | - | - | - | - | - | - | - | - | - |
| H8 | - | - | - | - | - | - | - | - | - | - | 4 | 2 | 5 | 2 |
| H9 | - | - | - | - | - | - | - | - | - | - | 1 | - | 1 | - |
| H10 | 1 | - | - | - | - | - | - | - | - | - | 1 | - | - | - |
| H11 | - | - | - | - | - | - | - | - | - | - | - | 1 | - | - |
| H12 | 1 | 2 | - | - | - | - | - | - | - | - | - | - | - | - |
| H13 | - | - | - | - | - | - | - | - | - | - | - | 1 | - | - |
| H14 | 3 | 1 | - | - | - | - | 1 | 1 | - | - | - | - | - | - |
| H15 | 2 | 1 | - | - | - | - | 1 | - | - | - | - | - | - | - |
| H16 | - | - | - | - | 2 | - | - | - | - | - | - | - | - | - |
| H17 | - | - | - | - | - | 1 | - | - | - | - | - | - | - | - |
| H18 | - | - | - | - | 1 | - | - | - | - | - | - | - | - | - |
| H19 | 3 | 12 | - | - | - | - | 1 | - | - | 1 | 1 | 1 | 12 | 3 |
| H20 | - | - | - | - | - | - | - | - | 1 | - | - | - | - | - |
| H21 | - | - | - | - | - | - | - | - | - | - | - | - | 1 | - |
| H22 | - | - | 1 | - | - | - | - | - | - | - | - | - | - | 1 |
| H23 | - | - | - | - | - | - | - | - | - | - | - | 1 | - | - |
| H24 | - | - | 1 | 1 | - | - | - | - | - | - | - | - | - | - |
| H25 | - | - | - | - | - | - | - | - | - | - | - | - | 1 | 3 |
| H26 | - | - | 5 | 3 | 1 | - | 1 | - | - | - | 4 | 2 | - | 1 |
| H27 | - | - | - | - | 1 | - | - | - | - | - | - | - | - | - |
| H28 | 1 | 1 | - | - | - | - | - | - | 2 | 1 | - | - | - | - |
| H29 | - | - | - | - | - | - | - | - | 1 | 1 | - | - | - | 1 |
| H30 | 2 | - | - | - | - | - | - | - | - | - | - | - | - | - |
| H31 | 1 | - | - | - | - | - | - | - | - | - | - | - | - | - |
| H32 | - | - | - | - | - | - | - | - | - | - | - | - | 1 | - |
| H33 | - | - | 1 | - | 1 | - | - | 2 | 2 | - | 1 | 2 | - | - |
| H34 | - | - | 6 | - | 8 | - | 6 | 8 | 8 | 2 | 12 | 12 | 26 | 8 |
| H35 | - | - | - | - | - | - | - | - | - | - | - | - | - | 1 |
| H36 | - | - | - | - | - | - | - | - | - | - | - | - | 4 | - |
| H37 | - | - | - | - | - | - | 1 | - | - | - | - | - | - | - |
| H38 | - | - | - | - | - | - | - | - | - | - | 1 | - | - | 1 |
| H39 | - | - | - | - | - | - | - | 1 | - | - | - | - | - | - |
| H40 | - | - | 4 | 1 | 5 | 1 | 7 | 5 | 2 | 1 | - | - | - | - |
| H41 | - | - | 1 | - | 1 | - | - | - | - | - | - | - | - | - |
| H42 | - | - | - | 1 | - | - | - | - | - | - | - | - | - | - |
| H43 | - | - | 3 | 1 | - | - | - | - | - | - | - | - | - | - |
| H44 | - | - | - | - | 4 | - | - | - | - | - | - | - | - | - |
| H45 | - | - | - | - | - | - | - | - | 1 | - | - | - | - | - |
| H46 | 5 | 3 | 22 | 25 | 34 | 5 | 27 | 21 | 17 | 8 | 1 | 1 | - | - |
| H47 | - | - | - | - | - | - | - | 1 | - | - | - | - | - | - |
| H48 | - | - | - | - | - | - | - | 1 | - | - | - | - | - | - |
| H49 | - | - | 2 | - | 1 | - | - | - | 1 | - | - | - | - | - |
| H50 | - | - | 11 | 15 | 22 | - | 14 | 10 | 18 | 4 | - | - | - | 2 |
| H51 | - | - | 1 | - | - | - | - | - | - | - | - | - | - | - |
| H52 | - | - | - | - | - | - | - | 1 | - | - | - | - | - | - |
| H53 | - | - | - | - | 1 | - | - | - | - | - | - | - | - | - |
| H54 | 1 | 1 | - | - | - | - | 1 | - | 1 | - | - | - | - | - |
| H55 | - | - | - | - | 2 | - | - | - | - | - | - | - | - | - |
| H56 | - | 1 | - | - | - | - | - | - | - | - | - | - | - | - |
| H57 | 1 | - | - | - | - | - | - | - | - | - | - | - | - | - |
| H58 | 1 | 7 | 2 | - | - | - | - | 1 | - | - | - | - | - | - |
| H59 | - | 1 | - | - | - | - | - | - | - | - | - | - | - | - |
| H60 | - | - | - | - | - | - | - | - | - | - | - | - | - | 1 |
| H61 | - | - | - | - | - | - | - | - | - | - | - | - | 2 | 1 |
| H62 | - | - | - | - | 1 | - | - | - | - | - | - | - | 2 | - |
| H63 | - | - | 1 | 1 | - | - | - | - | - | - | - | - | - | - |
| H64 | - | - | 16 | 11 | 10 | 2 | 16 | 16 | 14 | 4 | 7 | 4 | 17 | 2 |
| H65 | - | - | - | - | - | - | - | - | - | - | - | 1 | 2 | 2 |
| H66 | 11 | 14 | - | - | - | - | 2 | - | - | - | 22 | 22 | 61 | 17 |
| H67 | - | - | - | - | - | - | - | - | - | - | - | - | 8 | 1 |
| H68 | - | - | - | - | - | - | - | - | - | - | - | - | - | 1 |
| H69 | - | - | - | - | - | - | - | - | - | - | - | - | 1 | - |
| H70 | - | - | - | - | - | - | - | - | - | - | - | 1 | - | - |
| H71 | - | - | - | - | - | - | - | - | - | - | - | 1 | 1 | - |
| H72 | - | - | - | - | - | - | - | - | - | - | 1 | 2 | 2 | - |
| H73 | - | - | - | - | - | - | - | - | - | - | 3 | 2 | - | - |
| H74 | - | - | - | - | - | - | - | - | - | - | - | - | - | 1 |
| H75 | - | - | - | - | - | - | - | - | - | - | 1 | - | 9 | - |
| H76 | - | - | - | - | - | - | - | - | - | - | - | - | - | 1 |
| H77 | - | - | - | - | - | - | - | - | - | - | - | - | - | 1 |
| H78 | - | - | - | - | - | - | - | - | - | - | - | - | - | 2 |
| H79 | - | - | - | - | - | - | - | - | - | - | 1 | 2 | - | - |
| H80 | - | - | - | 1 | - | - | - | - | - | - | - | - | 2 | - |
| H81 | - | - | - | - | - | - | 1 | - | - | - | 1 | - | 14 | 6 |
| H82 | - | - | - | - | - | - | - | - | 1 | - | - | - | 1 | - |
| H83 | 9 | 4 | 16 | 10 | 15 | 2 | 16 | 14 | 8 | 6 | 16 | 33 | 32 | 21 |
| H84 | - | - | - | - | - | - | - | - | - | - | - | - | 3 | - |
| H85 | - | - | - | - | - | - | - | - | - | - | 1 | - | - | - |
| H86 | 3 | - | - | - | - | - | - | - | - | - | - | - | - | - |
| H87 | - | - | 1 | 1 | - | - | - | - | - | - | - | - | - | - |
| H88 | - | 1 | - | - | - | - | - | - | - | - | - | - | - | - |
| H89 | - | - | - | - | - | - | - | - | - | - | - | 1 | - | - |
| H90 | 1 | - | - | - | - | - | - | - | - | - | - | - | - | - |

[[1]](#footnote-2) 

**B**. *P*-values of Haplotype H26 in CN-SZ pairs

|  | **GE** | |  | **US** | |  | **JP** | |
| --- | --- | --- | --- | --- | --- | --- | --- | --- |
|  | **M** | **F** |  | **M** | **F** |  | **M** | **F** |
| Total number of haplotypes in CN | 228 | 152 |  | 262 | 252 |  | 190 | 200 |
| Observed number of H26 in CN | 5 | 3 |  | 1 | 0 |  | 4 | 2 |
| Total number of haplotypes in SZ | 286 | 66 |  | 212 | 88 |  | 406 | 184 |
| Observed number of H26 in SZ | 1 | 0 |  | 0 | 0 |  | 0 | 1 |
| Expected number of H26 in SZ ^a^ | 6 | 1 |  | 1 | 0 |  | 9 | 2 |
| *P_U_*-value ^b^ | 0.0532 | 0.250 |  | 0.368 | - |  | ***0.00335*** | 0.612 |
| *P_C_*-value ^c^ | ***0.0391*** | 0.314 |  | 0.316 | - |  | ***0.00242*** | 0.477 |
| *P_P_*-value ^d^ | ***0.0174*** | 0.368 |  | 0.368 | - |  | ***0.000123*** | 0.736 |

^a^ **Estimation of the expected number of a haplotype.** Assuming that both the CN and SZ cohorts of the same gender in the same ethnic population were derived from a single population, the observed count of any haplotype in the higher-frequency cohort could be employed to estimate the expected count in the lower-frequency cohort. For example, in the JP male CN cohort, there were 190 chromosomes in total of which 4 chromosomes were inferred to contain H26; since there were 406 chromosomes in the JP male SZ cohort, the expected number of H26 in the SZ cohort was estimated to be 4 × (406 / 190) = 8.55 or 9 by taking its nearest integer value.

^b^ ***P*-values for observing the actual count of an inferred haplotype in the lower-frequency cohort estimated using log-likelihood ratio test.** P-values were calculated using log-likelihood ratio test in UNPHASED and represented as *P_U_.*

^c^ ***P*-values for observing the actual count of an inferred haplotype in the lower-frequency cohort estimated using χ^2^ goodness-of-fit test.** Using the expected number of a haplotype derived in footnote a, the probability of observing the actual haplotype count in the lower frequency cohort was calculated using χ^2^ goodness-of-fit test and represented as *P_C_.*

^d^ ***P*-values for observing the actual count of an inferred haplotype in the lower-frequency cohort estimated using Poisson distribution.** Assuming that the number of counts of a given haplotype in a population is a Poisson distribution, based on the expected count (μ), the probability of an observed count (*x*) being equal to or less than *x* is given by:

$$P(x)=\sum_{y=0}^{x} \frac{e^{-\mu}\mu^{y}}{y!}$$

For example, in JP male, the expected count of H26 in the SZ cohort was 9 whereas the observed count was 0. It follows that

$$\begin{matrix} P(x=0)=\frac{e^{-9}9^{0}}{0!} \\ =e^{-9} \\ =0.000123 \end{matrix}$$

Therefore the probability of encountering a population of 406 chromosomes in SZ cohort with zero H26 count was 0.000123. Cases where a zero expected count rendered the Poisson procedure inapplicable, as in the case of the US F cohort, are indicated by “-“. The *P*-value calculated using Poisson distribution is represented by *P_P_.*

**C**. *P*-values of Haplotype H73 in CN-SZ pairs

|  | **GE** | |  | **US** | |  | **JP** | |
| --- | --- | --- | --- | --- | --- | --- | --- | --- |
|  | **M** | **F** |  | **M** | **F** |  | **M** | **F** |
| Total number of haplotypes in CN | 228 | 152 |  | 262 | 252 |  | 190 | 200 |
| Observed number of H73 in CN | 0 | 0 |  | 0 | 0 |  | 3 | 2 |
| Total number of haplotypes in SZ | 286 | 66 |  | 212 | 88 |  | 406 | 184 |
| Observed number of H73 in SZ | 0 | 0 |  | 0 | 0 |  | 0 | 0 |
| Expected number of H73 in SZ | 0 | 0 |  | 0 | 0 |  | 6 | 2 |
| *P_U_*-value | - | - |  | - | - |  | ***0.0111*** | 0.174 |
| *P_C_*-value | - | - |  | - | - |  | ***0.0136*** | 0.155 |
| *P_P_*-value | - | - |  | - | - |  | ***0.00248*** | 0.135 |

**D**. *P*-values of Haplotype H19 in CN-SZ pairs

|  | **GE** | |  | **US** |  | **JP** | |
| --- | --- | --- | --- | --- | --- | --- | --- |
|  | **M** | **F** |  | **F** |  | **M** | **F** |
| Total number of haplotypes in SZ | 286 | 66 |  | 88 |  | 406 | 184 |
| Observed number of H19 in SZ | 0 | 0 |  | 1 |  | 12 ^#^ | 3 |
| Total number of haplotypes in CN | 228 | 152 |  | 252 |  | 190 | 200 |
| Observed number of H19 in CN | 0 | 0 |  | 0 |  | 1 | 1 |
| Expected number of H19 in CN | 0 | 0 |  | 3 |  | 6 | 3 |
| *P_U_*-value | - | - |  | 0.0901 |  | 0.0585 | 0.276 |
| *P_C_*-value | - | - |  | 0.0814 |  | ***0.0381*** | 0.245 |
| *P_P_*-value | - | - |  | ***0.0498*** |  | ***0.0174*** | 0.199 |

^#^ 4 homozygous and 4 heterozygous individuals

|  | **US** |
| --- | --- |
|  | **M** |
| Total number of haplotypes in CN | 262 |
| Observed number of H19 in CN | 1 |
| Total number of haplotypes in SZ | 212 |
| Observed number of H19 in SZ | 0 |
| Expected number of H19 in SZ | 1 |
| *P_U_*-value | 0.368 |
| *P_C_*-value | 0.316 |
| *P_P_*-value | 0.368 |

**E**. *P*-values of Haplotype H81 in CN-SZ pairs

|  | **GE** | |  | **US** |  | **JP** | |
| --- | --- | --- | --- | --- | --- | --- | --- |
|  | **M** | **F** |  | **F** |  | **M** | **F** |
| Total number of haplotypes in SZ | 286 | 66 |  | 88 |  | 406 | 184 |
| Observed number of H81 in SZ | 0 | 0 |  | 0 |  | 14* | 6 |
| Total number of haplotypes in CN | 228 | 152 |  | 252 |  | 190 | 200 |
| Observed number of H81 in CN | 0 | 0 |  | 0 |  | 1 | 0 |
| Expected number of H81 in CN | 0 | 0 |  | 0 |  | 7 | 7 |
| *P_U_*-value | - | - |  | - |  | ***0.0338*** | ***0.0101*** |
| *P_C_*-value | - | - |  | - |  | ***0.0208*** | ***0.00708*** |
| *P_P_*-value | - | - |  | - |  | ***0.00729*** | ***0.000912*** |

* One homozygous and 12 heterozygous individuals

|  | **US** |
| --- | --- |
|  | **M** |
| Total number of haplotypes in CN | 262 |
| Observed number of H81 in CN | 1 |
| Total number of haplotypes in SZ | 212 |
| Observed number of H81 in SZ | 0 |
| Expected number of H81 in SZ | 1 |
| *P_U_*-value | 0.368 |
| *P_C_*-value | 0.316 |
| *P_P_*-value | 0.368 |

1.    AF, African; GE, German Caucasian; US, American Caucasian; JP, Japanese; F, female; M, male; CN, controls; SZ, schizophrenics. [↑](#footnote-ref-2)
