# Supplementary material for: A Recombination Hotspot in a Schizophrenia-Associated Region of GABRB2
Source: PLoS One. 2010 Mar 8;5(3):e9547. doi: 10.1371/journal.pone.0009547 (PMC2833194; doi:10.1371/journal.pone.0009547)
Supplement: Table S4 — Detection of recombination between two target SNPs (SNP X and SNP Y) by cloning and allele-specific real-time PCR. (0.06 MB DOC) [file pone.0009547.s006.doc]

**Table S4** Detection of recombination between two target SNPs (SNP X and SNP Y) by cloning and allele-specific real-time PCR.

**A**. Cloning of sperm DNA

| DNA sample | SNP X | SNP Y | Clones obtained | Recombinant clones observed | Genetic X-Y distance (cM)^a^ | Physical X-Y distance (bp) | Recombination  Rate (cM/Mb)^b^ |
| --- | --- | --- | --- | --- | --- | --- | --- |
| Genome 1^c^ | S3^d^ | S5 | 43 | 0 | 0.00 | 300 | 0.0 |
| Sperm 1 | S3 | S5 | 141 | 2 | 1.42 | 300 | 4,728.1 |
| Sperm 2 | S2 | S4 | 91 | 0 | 0.00 | 123 | 0.0 |
|  | S4 | S5 | 91 | 1 | 1.10 | 229 | 4,798.7 |

cM, centimorgan; Mb, megabase.

^a^ Genetic distance is the fraction of recombinant clones among total clones obtained.

^b^ Recombination rate is calculated by genetic distance over the physical distance.

^c^ Genome 1 is obtained from donor of Sperm 1.

^d^ See Figure S1a for numbering of different SNPs in the 3,551-bp segment.

**B**. Allele-specific real-time PCR (AS-RT-PRC) of Sperm sample 1

| SNP X | SNP Y | No. of  crossovers | No. of amplifiable genomes | Genetic X-Y distance (cM) | Physical X-Y distance (bp) | Recombination  Rate (cM/Mb)^a^ |
| --- | --- | --- | --- | --- | --- | --- |
| S3 | S5 | 3 | 7,700 | 3.90 x 10^-2^ | 300 | 129.9 |
| S5 | S15 | 5 | 7,700 | 6.49 x 10^-2^ | 688 | 94.4 |

^a^ Recombination calculated by the method as described.^10^
