# Supplementary material for: A Recombination Hotspot in a Schizophrenia-Associated Region of GABRB2
Source: PLoS One. 2010 Mar 8;5(3):e9547. doi: 10.1371/journal.pone.0009547 (PMC2833194; doi:10.1371/journal.pone.0009547)
Supplement: Table S5 — Pairwise SNP linkage disequilibrium (LD) r∧2 values. (0.72 MB DOC) [file pone.0009547.s007.doc]

**Table S5** Pairwise SNP linkage disequilibrium (LD) *r*^2^ values

| SNP X | SNP Y | AF | | GE | | | | US | | | | JP | | | |
| --- | --- | --- | --- | --- | --- | --- | --- | --- | --- | --- | --- | --- | --- | --- | --- |
|  |  | F | M | CN | | SZ | | CN | | SZ | | CN | | SZ | |
|  |  |  |  | F | M | F | M | F | M | F | M | F | M | F | M |
| S1 | S2 | <0.001 | 0.023 | - | - | - | - | - | - | - | - | 0.037 | 0.019 | 0.027 | 0.040 |
| S1 | S3 | 0.124 | 0.378 | 0.098 | 0.117 | 0.175 | 0.092 | 0.124 | 0.132 | 0.166 | 0.080 | 0.543 | 0.453 | 0.520 | 0.421 |
| S1 | S4 | <0.001 | 0.023 | - | - | - | - | - | - | - | - | 0.024 | 0.019 | 0.027 | 0.040 |
| S1 | S5 | 0.039 | 0.146 | 0.100 | 0.117 | 0.175 | 0.092 | 0.124 | 0.126 | 0.157 | 0.078 | 0.295 | 0.227 | 0.270 | 0.094 |
| S1 | S6 | 0.018 | 0.012 | 0.007 | 0.007 | 0.001 | 0.002 | 0.004 | 0.004 | 0.003 | 0.003 | 0.005 | 0.005 | 0.005 | 0.010 |
| S1 | S7 | 0.006 | 0.039 | 0.028 | 0.006 | 0.003 | 0.015 | 0.009 | 0.013 | 0.011 | 0.010 | 0.001 | 0.001 | - | 0.010 |
| S1 | S8 | 0.006 | 0.039 | 0.015 | 0.014 | 0.003 | 0.015 | 0.009 | 0.013 | 0.011 | 0.010 | 0.001 | 0.001 | - | 0.010 |
| S1 | S9 | - | - | 0.007 | 0.007 | 0.001 | 0.003 | 0.004 | 0.004 | 0.003 | 0.003 | 0.005 | 0.005 | 0.005 | 0.010 |
| S1 | S10 | 0.018 | 0.012 | 0.007 | 0.007 | 0.001 | 0.004 | 0.004 | 0.004 | 0.003 | 0.003 | 0.005 | 0.005 | 0.005 | 0.010 |
| S1 | S11 | 0.002 | 0.006 | - | - | - | - | - | <0.001 | - | - | - | - | - | - |
| S1 | S12 | 0.076 | 0.053 | - | - | - | - | <0.001 | <0.001 | - | - | - | - | - | - |
| S1 | S13 | 0.004 | 0.006 | - | - | - | - | - | <0.001 | - | <0.001 | - | - | - | - |
| S1 | S14 | - | - | 0.002 | 0.003 | <0.001 | 0.001 | 0.001 | 0.002 | 0.001 | <0.001 | - | - | - | - |
| S1 | S15 | - | - | 0.001 | 0.004 | <0.001 | - | 0.002 | 0.002 | 0.002 | 0.002 | 0.017 | 0.002 | 0.017 | 0.015 |
| S1 | S16 | - | - | 0.002 | 0.003 | <0.001 | 0.001 | 0.001 | 0.002 | 0.001 | <0.001 | - | - | - | - |
| S1 | S17 | - | - | 0.002 | 0.003 | <0.001 | 0.001 | 0.001 | 0.002 | 0.001 | <0.001 | - | - | - | - |
| S1 | S18 | 0.004 | 0.006 | - | - | - | - | - | - | - | - | - | - | - | - |
| S1 | S19 | 0.002 | - | - | - | - | - | - | - | - | - | - | - | - | - |
| S1 | S20 | 0.783 | 0.901 | 0.397 | 0.513 | 0.656 | 0.350 | 0.511 | 0.517 | 0.570 | 0.269 | 0.717 | 0.667 | 0.787 | 0.908 |
| S1 | S21 | - | 0.012 | - | - | - | - | <0.001 | - | - | - | - | - | - | - |
| S1 | S22 | - | - | 0.002 | 0.003 | <0.001 | 0.001 | 0.001 | 0.002 | 0.001 | <0.001 | - | - | - | - |
| S1 | S23 | - | 0.012 | - | - | - | - | - | - | - | - | - | - | - | - |
| S1 | S24 | 0.783 | 1.000 | 0.397 | 0.513 | 0.656 | 0.350 | 0.490 | 0.517 | 0.570 | 0.269 | 0.638 | 0.654 | 0.817 | 0.874 |
| S1 | S25 | - | - | - | - | - | - | - | - | - | - | 0.003 | 0.003 | 0.003 | 0.003 |
| S1 | S26 | - | - | - | - | - | - | - | <0.001 | - | - | - | - | - | - |
| S1 | S27 | 0.124 | 0.378 | 0.081 | 0.155 | 0.156 | 0.110 | 0.147 | 0.127 | 0.184 | 0.096 | 0.653 | 0.438 | 0.366 | 0.331 |
| S1 | S28 | 0.018 | 0.006 | 0.007 | 0.008 | 0.001 | 0.003 | 0.004 | 0.005 | 0.004 | 0.003 | 0.005 | 0.006 | 0.006 | <0.001 |
| S1 | S29 | 0.124 | 0.378 | 0.084 | 0.138 | 0.156 | 0.106 | 0.144 | 0.147 | 0.184 | 0.066 | 0.634 | 0.516 | 0.676 | 0.632 |
| S2 | S3 | 0.120 | 0.113 | - | - | - | - | - | - | - | - | 0.060 | 0.042 | 0.052 | 0.086 |
| S2 | S4 | 1.000 | 1.000 | - | - | - | - | - | - | - | - | 0.914 | 1.000 | 1.000 | 1.000 |
| S2 | S5 | 0.110 | 0.116 | - | - | - | - | - | - | - | - | 0.184 | 0.235 | 0.147 | 0.228 |
| S2 | S6 | 0.050 | 0.010 | - | - | - | - | - | - | - | - | 0.003 | 0.005 | 0.003 | 0.012 |
| S2 | S7 | 0.017 | 0.031 | - | - | - | - | - | - | - | - | 0.001 | 0.001 | - | 0.012 |
| S2 | S8 | 0.017 | 0.031 | - | - | - | - | - | - | - | - | 0.001 | 0.001 | - | 0.012 |
| S2 | S9 | - | - | - | - | - | - | - | - | - | - | 0.003 | 0.005 | 0.003 | 0.012 |
| S2 | S10 | 0.050 | 0.010 | - | - | - | - | - | - | - | - | 0.003 | 0.005 | 0.003 | 0.012 |
| S2 | S11 | 0.005 | 0.005 | - | - | - | - | - | - | - | - | - | - | - | - |
| S2 | S12 | 0.017 | 0.031 | - | - | - | - | - | - | - | - | - | - | - | - |
| S2 | S13 | 0.011 | 0.005 | - | - | - | - | - | - | - | - | - | - | - | - |
| S2 | S15 | - | - | - | - | - | - | - | - | - | - | 0.010 | 0.010 | 0.011 | 0.018 |
| S2 | S18 | 0.011 | 0.005 | - | - | - | - | - | - | - | - | - | - | - | - |
| S2 | S19 | 0.005 | - | - | - | - | - | - | - | - | - | - | - | - | - |
| S2 | S20 | 0.023 | 0.029 | - | - | - | - | - | - | - | - | 0.027 | 0.021 | 0.027 | 0.033 |
| S2 | S21 | - | 0.010 | - | - | - | - | - | - | - | - | - | - | - | - |
| S2 | S23 | - | 0.010 | - | - | - | - | - | - | - | - | - | - | - | - |
| S2 | S24 | 0.023 | 0.023 | - | - | - | - | - | - | - | - | 0.015 | 0.019 | 0.026 | 0.035 |
| S2 | S25 | - | - | - | - | - | - | - | - | - | - | 0.002 | 0.003 | 0.002 | <0.001 |
| S2 | S27 | 0.120 | 0.113 | - | - | - | - | - | - | - | - | 0.043 | 0.028 | 0.008 | 0.010 |
| S2 | S28 | 0.050 | 0.005 | - | - | - | - | - | - | - | - | 0.003 | 0.006 | 0.002 | 0.012 |
| S2 | S29 | 0.120 | 0.113 | - | - | - | - | - | - | - | - | 0.031 | 0.027 | 0.031 | 0.053 |
| S3 | S4 | 0.120 | 0.113 | - | - | - | - | - | - | - | - | 0.045 | 0.042 | 0.052 | 0.086 |
| S3 | S5 | 0.162 | 0.386 | 0.974 | 0.964 | 1.000 | 1.000 | 1.000 | 0.951 | 0.949 | 0.980 | 0.562 | 0.534 | 0.461 | 0.317 |
| S3 | S6 | 0.413 | 0.051 | 0.098 | 0.132 | 0.175 | 0.055 | 0.153 | 0.125 | 0.108 | 0.127 | 0.051 | 0.120 | 0.055 | 0.140 |
| S3 | S7 | 0.140 | 0.165 | 0.367 | 0.281 | 0.361 | 0.444 | 0.329 | 0.365 | 0.356 | 0.391 | 0.013 | 0.017 | - | 0.140 |
| S3 | S8 | 0.140 | 0.165 | 0.421 | 0.254 | 0.361 | 0.444 | 0.329 | 0.365 | 0.356 | 0.391 | 0.013 | 0.017 | - | 0.140 |
| S3 | S9 | - | - | 0.098 | 0.117 | 0.175 | 0.092 | 0.143 | 0.125 | 0.108 | 0.127 | 0.051 | 0.120 | 0.055 | 0.140 |
| S3 | S10 | 0.413 | 0.051 | 0.098 | 0.132 | 0.175 | 0.082 | 0.153 | 0.125 | 0.108 | 0.127 | 0.051 | 0.120 | 0.055 | 0.140 |
| S3 | S11 | 0.008 | 0.016 | - | - | - | - | - | 0.002 | - | - | - | - | - | - |
| S3 | S12 | 0.016 | 0.002 | - | - | - | - | 0.002 | 0.002 | - | - | - | - | - | - |
| S3 | S13 | 0.016 | 0.025 | - | - | - | - | - | 0.007 | - | 0.008 | - | - | - | - |
| S3 | S14 | - | - | 0.023 | 0.053 | 0.086 | 0.042 | 0.052 | 0.052 | 0.026 | 0.017 | - | - | - | - |
| S3 | S15 | - | - | 0.015 | 0.060 | 0.086 | - | 0.078 | 0.052 | 0.052 | 0.070 | 0.139 | 0.249 | 0.165 | 0.212 |
| S3 | S16 | - | - | 0.023 | 0.053 | 0.086 | 0.036 | 0.052 | 0.052 | 0.026 | 0.017 | - | - | - | - |
| S3 | S17 | - | - | 0.023 | 0.053 | 0.086 | 0.036 | 0.052 | 0.052 | 0.026 | 0.017 | - | - | - | - |
| S3 | S18 | 0.016 | 0.016 | - | - | - | - | - | - | - | - | - | - | - | - |
| S3 | S19 | 0.045 | - | - | - | - | - | - | - | - | - | - | - | - | - |
| S3 | S20 | 0.190 | 0.312 | 0.246 | 0.228 | 0.099 | 0.264 | 0.243 | 0.256 | 0.289 | 0.295 | 0.501 | 0.315 | 0.520 | 0.450 |
| S3 | S21 | - | 0.051 | - | - | - | - | 0.008 | - | - | - | - | - | - | - |
| S3 | S22 | - | - | 0.023 | 0.053 | 0.086 | 0.036 | 0.052 | 0.052 | 0.026 | 0.017 | - | - | - | - |
| S3 | S23 | - | 0.051 | - | - | - | - | - | - | - | - | - | - | - | - |
| S3 | S24 | 0.190 | 0.378 | 0.246 | 0.228 | 0.099 | 0.264 | 0.253 | 0.256 | 0.289 | 0.295 | 0.445 | 0.276 | 0.501 | 0.428 |
| S3 | S25 | - | - | - | - | - | - | - | - | - | - | 0.004 | 0.007 | <0.001 | 0.006 |
| S3 | S26 | - | - | - | - | - | - | - | 0.007 | - | - | - | - | - | - |
| S3 | S27 | 1.000 | 1.000 | 0.876 | 0.722 | 0.893 | 0.845 | 0.845 | 0.839 | 0.897 | 0.789 | 0.640 | 0.421 | 0.284 | 0.411 |
| S3 | S28 | 0.413 | 0.025 | 0.098 | 0.117 | 0.175 | 0.086 | 0.153 | 0.110 | 0.136 | 0.136 | 0.051 | 0.096 | 0.013 | 0.122 |
| S3 | S29 | 1.000 | 1.000 | 0.805 | 0.686 | 0.893 | 0.845 | 0.861 | 0.837 | 0.897 | 0.769 | 0.619 | 0.487 | 0.601 | 0.619 |
| S4 | S5 | 0.110 | 0.116 | - | - | - | - | - | - | - | - | 0.184 | 0.235 | 0.147 | 0.228 |
| S4 | S6 | 0.050 | 0.010 | - | - | - | - | - | - | - | - | 0.003 | 0.005 | 0.003 | 0.012 |
| S4 | S7 | 0.017 | 0.031 | - | - | - | - | - | - | - | - | 0.001 | 0.001 | - | 0.012 |
| S4 | S8 | 0.017 | 0.031 | - | - | - | - | - | - | - | - | 0.001 | 0.001 | - | 0.012 |
| S4 | S9 | - | - | - | - | - | - | - | - | - | - | 0.003 | 0.005 | 0.003 | 0.012 |
| S4 | S10 | 0.050 | 0.010 | - | - | - | - | - | - | - | - | 0.003 | 0.005 | 0.003 | 0.012 |
| S4 | S11 | 0.005 | 0.005 | - | - | - | - | - | - | - | - | - | - | - | - |
| S4 | S12 | 0.017 | 0.031 | - | - | - | - | - | - | - | - | - | - | - | - |
| S4 | S13 | 0.011 | 0.005 | - | - | - | - | - | - | - | - | - | - | - | - |
| S4 | S15 | - | - | - | - | - | - | - | - | - | - | 0.010 | 0.010 | 0.011 | 0.018 |
| S4 | S18 | 0.011 | 0.005 | - | - | - | - | - | - | - | - | - | - | - | - |
| S4 | S19 | 0.005 | - | - | - | - | - | - | - | - | - | - | - | - | - |
| S4 | S20 | 0.023 | 0.029 | - | - | - | - | - | - | - | - | 0.016 | 0.021 | 0.027 | 0.033 |
| S4 | S21 | - | 0.010 | - | - | - | - | - | - | - | - | - | - | - | - |
| S4 | S23 | - | 0.010 | - | - | - | - | - | - | - | - | - | - | - | - |
| S4 | S24 | 0.023 | 0.023 | - | - | - | - | - | - | - | - | 0.007 | 0.019 | 0.026 | 0.035 |
| S4 | S25 | - | - | - | - | - | - | - | - | - | - | 0.002 | 0.003 | 0.002 | <0.001 |
| S4 | S27 | 0.120 | 0.113 | - | - | - | - | - | - | - | - | 0.030 | 0.028 | 0.008 | 0.010 |
| S4 | S28 | 0.050 | 0.005 | - | - | - | - | - | - | - | - | 0.003 | 0.006 | 0.002 | 0.012 |
| S4 | S29 | 0.120 | 0.113 | - | - | - | - | - | - | - | - | 0.020 | 0.027 | 0.031 | 0.053 |
| S5 | S6 | 0.067 | 0.019 | 0.100 | 0.132 | 0.175 | 0.055 | 0.153 | 0.118 | 0.102 | 0.124 | 0.029 | 0.069 | 0.035 | 0.063 |
| S5 | S7 | 0.022 | 0.064 | 0.377 | 0.281 | 0.361 | 0.444 | 0.329 | 0.347 | 0.339 | 0.383 | 0.007 | 0.009 | - | 0.063 |
| S5 | S8 | 0.022 | 0.064 | 0.433 | 0.254 | 0.361 | 0.444 | 0.329 | 0.347 | 0.339 | 0.383 | 0.007 | 0.009 | - | 0.063 |
| S5 | S9 | - | - | 0.100 | 0.117 | 0.175 | 0.092 | 0.143 | 0.118 | 0.102 | 0.124 | 0.029 | 0.069 | 0.035 | 0.063 |
| S5 | S10 | 0.067 | 0.019 | 0.100 | 0.132 | 0.175 | 0.082 | 0.153 | 0.118 | 0.102 | 0.124 | 0.029 | 0.069 | 0.035 | 0.063 |
| S5 | S11 | 0.049 | 0.040 | - | - | - | - | - | 0.002 | - | - | - | - | - | - |
| S5 | S12 | 0.009 | 0.018 | - | - | - | - | 0.002 | 0.002 | - | - | - | - | - | - |
| S5 | S13 | 0.100 | 0.010 | - | - | - | - | - | 0.007 | - | 0.008 | - | - | - | - |
| S5 | S14 | - | - | 0.024 | 0.053 | 0.086 | 0.042 | 0.052 | 0.050 | 0.025 | 0.017 | - | - | - | - |
| S5 | S15 | - | - | 0.016 | 0.081 | 0.086 | - | 0.078 | 0.050 | 0.050 | 0.069 | 0.074 | 0.143 | 0.056 | 0.085 |
| S5 | S16 | - | - | 0.024 | 0.053 | 0.086 | 0.036 | 0.052 | 0.050 | 0.025 | 0.017 | - | - | - | - |
| S5 | S17 | - | - | 0.024 | 0.053 | 0.086 | 0.036 | 0.052 | 0.050 | 0.025 | 0.017 | - | - | - | - |
| S5 | S18 | 0.100 | 0.040 | - | - | - | - | - | - | - | - | - | - | - | - |
| S5 | S19 | 0.007 | - | - | - | - | - | - | - | - | - | - | - | - | - |
| S5 | S20 | 0.031 | 0.095 | 0.253 | 0.228 | 0.099 | 0.264 | 0.243 | 0.244 | 0.275 | 0.289 | 0.288 | 0.140 | 0.270 | 0.115 |
| S5 | S21 | - | 0.019 | - | - | - | - | 0.008 | - | - | - | - | - | - | - |
| S5 | S22 | - | - | 0.024 | 0.053 | 0.086 | 0.036 | 0.052 | 0.050 | 0.025 | 0.017 | - | - | - | - |
| S5 | S23 | - | 0.019 | - | - | - | - | - | - | - | - | - | - | - | - |
| S5 | S24 | 0.031 | 0.146 | 0.253 | 0.228 | 0.099 | 0.264 | 0.253 | 0.244 | 0.275 | 0.289 | 0.278 | 0.118 | 0.258 | 0.112 |
| S5 | S25 | - | - | - | - | - | - | - | - | - | - | 0.007 | 0.012 | 0.010 | 0.005 |
| S5 | S26 | - | - | - | - | - | - | - | 0.007 | - | - | - | - | - | - |
| S5 | S27 | 0.162 | 0.386 | 0.899 | 0.722 | 0.893 | 0.845 | 0.845 | 0.797 | 0.850 | 0.810 | 0.349 | 0.194 | 0.205 | 0.150 |
| S5 | S28 | 0.067 | 0.010 | 0.100 | 0.117 | 0.175 | 0.086 | 0.153 | 0.104 | 0.129 | 0.133 | 0.029 | 0.051 | 0.020 | 0.052 |
| S5 | S29 | 0.162 | 0.386 | 0.826 | 0.686 | 0.893 | 0.845 | 0.861 | 0.796 | 0.850 | 0.790 | 0.361 | 0.235 | 0.387 | 0.174 |
| S6 | S7 | 0.010 | 0.005 | 0.028 | 0.018 | 0.003 | 0.012 | 0.011 | 0.012 | 0.008 | 0.015 | <0.001 | <0.001 | - | 1.000 |
| S6 | S8 | 0.010 | 0.005 | 0.032 | 0.016 | 0.003 | 0.012 | 0.011 | 0.012 | 0.008 | 0.015 | <0.001 | <0.001 | - | 1.000 |
| S6 | S9 | - | - | 1.000 | 0.885 | 1.000 | 0.658 | 0.937 | 1.000 | 1.000 | 1.000 | 1.000 | 1.000 | 1.000 | 1.000 |
| S6 | S10 | 1.000 | 1.000 | 1.000 | 1.000 | 1.000 | 0.510 | 1.000 | 1.000 | 1.000 | 1.000 | 1.000 | 1.000 | 1.000 | 1.000 |
| S6 | S11 | 0.003 | 0.001 | - | - | - | - | - | <0.001 | - | - | - | - | - | - |
| S6 | S12 | 0.007 | 0.005 | - | - | - | - | <0.001 | <0.001 | - | - | - | - | - | - |
| S6 | S13 | 0.007 | 0.001 | - | - | - | - | - | <0.001 | - | <0.001 | - | - | - | - |
| S6 | S14 | - | - | 0.002 | 0.003 | <0.001 | 0.001 | 0.002 | 0.002 | 0.001 | 0.001 | - | - | - | - |
| S6 | S15 | - | - | 0.001 | 0.005 | <0.001 | - | 0.003 | 0.002 | 0.001 | 0.003 | 0.001 | 0.003 | 0.002 | 0.004 |
| S6 | S16 | - | - | 0.002 | 0.003 | <0.001 | 0.001 | 0.002 | 0.002 | 0.001 | 0.001 | - | - | - | - |
| S6 | S17 | - | - | 0.002 | 0.003 | <0.001 | 0.001 | 0.002 | 0.002 | 0.001 | 0.001 | - | - | - | - |
| S6 | S18 | 0.007 | 0.001 | - | - | - | - | - | - | - | - | - | - | - | - |
| S6 | S19 | 0.108 | - | - | - | - | - | - | - | - | - | - | - | - | - |
| S6 | S20 | 0.014 | 0.013 | 0.018 | 0.014 | 0.002 | 0.007 | 0.008 | 0.009 | 0.006 | 0.012 | 0.005 | 0.006 | 0.005 | 0.010 |
| S6 | S21 | - | 0.002 | - | - | - | - | <0.001 | - | - | - | - | - | - | - |
| S6 | S22 | - | - | 0.002 | 0.003 | <0.001 | 0.001 | 0.002 | 0.002 | 0.001 | 0.001 | - | - | - | - |
| S6 | S23 | - | 0.002 | - | - | - | - | - | - | - | - | - | - | - | - |
| S6 | S24 | 0.014 | 0.012 | 0.018 | 0.014 | 0.002 | 0.007 | 0.009 | 0.009 | 0.006 | 0.012 | 0.005 | 0.005 | 0.004 | 0.010 |
| S6 | S25 | - | - | - | - | - | - | - | - | - | - | <0.001 | 0.001 | <0.001 | 0.001 |
| S6 | S26 | - | - | - | - | - | - | - | <0.001 | - | - | - | - | - | - |
| S6 | S27 | 0.413 | 0.051 | 0.112 | 0.175 | 0.156 | 0.066 | 0.181 | 0.143 | 0.120 | 0.153 | 0.070 | 0.182 | 0.013 | 0.178 |
| S6 | S28 | 1.000 | 0.490 | 1.000 | 0.945 | 1.000 | 0.579 | 1.000 | 0.937 | 0.790 | 0.929 | 1.000 | 0.870 | 0.188 | 0.638 |
| S6 | S29 | 0.413 | 0.051 | 0.084 | 0.131 | 0.156 | 0.064 | 0.177 | 0.138 | 0.120 | 0.150 | 0.068 | 0.189 | 0.043 | 0.226 |
| S7 | S8 | 1.000 | 1.000 | 0.870 | 0.904 | 1.000 | 1.000 | 1.000 | 1.000 | 1.000 | 1.000 | 1.000 | 1.000 | - | 1.000 |
| S7 | S9 | - | - | 0.028 | 0.016 | 0.003 | 0.015 | 0.011 | 0.012 | 0.008 | 0.015 | <0.001 | <0.001 | - | 1.000 |
| S7 | S10 | 0.010 | 0.005 | 0.028 | 0.018 | 0.003 | 0.005 | 0.011 | 0.012 | 0.008 | 0.015 | <0.001 | <0.001 | - | 1.000 |
| S7 | S11 | 0.001 | 0.003 | - | - | - | - | - | 0.001 | - | - | - | - | - | - |
| S7 | S12 | 0.002 | 0.017 | - | - | - | - | 0.001 | 0.001 | - | - | - | - | - | - |
| S7 | S13 | 0.002 | 0.151 | - | - | - | - | - | 0.020 | - | 0.022 | - | - | - | - |
| S7 | S14 | - | - | 0.006 | 0.007 | 0.002 | 0.001 | 0.004 | 0.005 | 0.002 | 0.002 | - | - | - | - |
| S7 | S15 | - | - | 0.004 | 0.003 | 0.002 | - | <0.001 | 0.005 | 0.004 | 0.009 | <0.001 | <0.001 | - | 0.004 |
| S7 | S16 | - | - | 0.006 | 0.007 | 0.002 | 0.006 | 0.004 | 0.005 | 0.002 | 0.002 | - | - | - | - |
| S7 | S17 | - | - | 0.006 | 0.007 | 0.002 | 0.006 | 0.004 | 0.005 | 0.002 | 0.002 | - | - | - | - |
| S7 | S18 | 0.002 | 0.003 | - | - | - | - | - | - | - | - | - | - | - | - |
| S7 | S19 | 0.001 | - | - | - | - | - | - | - | - | - | - | - | - | - |
| S7 | S20 | 0.005 | 0.044 | 0.089 | 0.097 | 0.034 | 0.138 | 0.062 | 0.085 | 0.076 | 0.231 | 0.001 | 0.001 | - | 0.010 |
| S7 | S21 | - | 0.005 | - | - | - | - | 0.026 | - | - | - | - | - | - | - |
| S7 | S22 | - | - | 0.006 | 0.007 | 0.002 | 0.006 | 0.004 | 0.005 | 0.002 | 0.002 | - | - | - | - |
| S7 | S23 | - | 0.005 | - | - | - | - | - | - | - | - | - | - | - | - |
| S7 | S24 | 0.005 | 0.039 | 0.089 | 0.097 | 0.034 | 0.138 | 0.076 | 0.085 | 0.076 | 0.231 | 0.001 | 0.001 | - | 0.010 |
| S7 | S25 | - | - | - | - | - | - | - | - | - | - | <0.001 | <0.001 | - | 0.001 |
| S7 | S26 | - | - | - | - | - | - | - | 0.001 | - | - | - | - | - | - |
| S7 | S27 | 0.140 | 0.165 | 0.419 | 0.286 | 0.500 | 0.500 | 0.389 | 0.419 | 0.398 | 0.403 | 0.017 | 0.025 | - | 0.178 |
| S7 | S28 | 0.010 | 0.003 | 0.028 | 0.018 | 0.003 | 0.014 | 0.011 | 0.013 | 0.010 | 0.017 | <0.001 | <0.001 | - | 0.638 |
| S7 | S29 | 0.140 | 0.165 | 0.430 | 0.305 | 0.500 | 0.510 | 0.382 | 0.404 | 0.398 | 0.428 | 0.017 | 0.026 | - | 0.226 |
| S8 | S9 | - | - | 0.032 | 0.014 | 0.003 | 0.015 | 0.011 | 0.012 | 0.008 | 0.015 | <0.001 | <0.001 | - | 1.000 |
| S8 | S10 | 0.010 | 0.005 | 0.032 | 0.016 | 0.003 | 0.005 | 0.011 | 0.012 | 0.008 | 0.015 | <0.001 | <0.001 | - | 1.000 |
| S8 | S11 | 0.001 | 0.003 | - | - | - | - | - | 0.001 | - | - | - | - | - | - |
| S8 | S12 | 0.002 | 0.017 | - | - | - | - | 0.001 | 0.001 | - | - | - | - | - | - |
| S8 | S13 | 0.002 | 0.151 | - | - | - | - | - | 0.020 | - | 0.022 | - | - | - | - |
| S8 | S14 | - | - | 0.007 | 0.006 | 0.002 | 0.001 | 0.004 | 0.005 | 0.002 | 0.002 | - | - | - | - |
| S8 | S15 | - | - | 0.005 | 0.010 | 0.002 | - | <0.001 | 0.005 | 0.004 | 0.009 | <0.001 | <0.001 | - | 0.004 |
| S8 | S16 | - | - | 0.007 | 0.006 | 0.002 | 0.006 | 0.004 | 0.005 | 0.002 | 0.002 | - | - | - | - |
| S8 | S17 | - | - | 0.007 | 0.006 | 0.002 | 0.006 | 0.004 | 0.005 | 0.002 | 0.002 | - | - | - | - |
| S8 | S18 | 0.002 | 0.003 | - | - | - | - | - | - | - | - | - | - | - | - |
| S8 | S19 | 0.001 | - | - | - | - | - | - | - | - | - | - | - | - | - |
| S8 | S20 | 0.005 | 0.044 | 0.114 | 0.091 | 0.034 | 0.138 | 0.062 | 0.085 | 0.076 | 0.231 | 0.001 | 0.001 | - | 0.010 |
| S8 | S21 | - | 0.005 | - | - | - | - | 0.026 | - | - | - | - | - | - | - |
| S8 | S22 | - | - | 0.007 | 0.006 | 0.002 | 0.006 | 0.004 | 0.005 | 0.002 | 0.002 | - | - | - | - |
| S8 | S23 | - | 0.005 | - | - | - | - | - | - | - | - | - | - | - | - |
| S8 | S24 | 0.005 | 0.039 | 0.114 | 0.091 | 0.034 | 0.138 | 0.076 | 0.085 | 0.076 | 0.231 | 0.001 | 0.001 | - | 0.010 |
| S8 | S25 | - | - | - | - | - | - | - | - | - | - | <0.001 | <0.001 | - | 0.001 |
| S8 | S26 | - | - | - | - | - | - | - | 0.001 | - | - | - | - | - | - |
| S8 | S27 | 0.140 | 0.165 | 0.482 | 0.280 | 0.500 | 0.500 | 0.389 | 0.419 | 0.398 | 0.403 | 0.017 | 0.025 | - | 0.178 |
| S8 | S28 | 0.010 | 0.003 | 0.032 | 0.017 | 0.003 | 0.014 | 0.011 | 0.013 | 0.010 | 0.017 | <0.001 | <0.001 | - | 0.638 |
| S8 | S29 | 0.140 | 0.165 | 0.494 | 0.300 | 0.500 | 0.510 | 0.382 | 0.404 | 0.398 | 0.428 | 0.017 | 0.026 | - | 0.226 |
| S9 | S10 | - | - | 1.000 | 0.885 | 1.000 | 0.778 | 0.937 | 1.000 | 1.000 | 1.000 | 1.000 | 1.000 | 1.000 | 1.000 |
| S9 | S11 | - | - | - | - | - | - | - | <0.001 | - | - | - | - | - | - |
| S9 | S12 | - | - | - | - | - | - | <0.001 | <0.001 | - | - | - | - | - | - |
| S9 | S13 | - | - | - | - | - | - | - | <0.001 | - | <0.001 | - | - | - | - |
| S9 | S14 | - | - | 0.002 | 0.003 | <0.001 | 0.001 | 0.002 | 0.002 | 0.001 | 0.001 | - | - | - | - |
| S9 | S15 | - | - | 0.001 | 0.004 | <0.001 | - | 0.003 | 0.002 | 0.001 | 0.003 | 0.001 | 0.003 | 0.002 | 0.004 |
| S9 | S16 | - | - | 0.002 | 0.003 | <0.001 | 0.001 | 0.002 | 0.002 | 0.001 | 0.001 | - | - | - | - |
| S9 | S17 | - | - | 0.002 | 0.003 | <0.001 | 0.001 | 0.002 | 0.002 | 0.001 | 0.001 | - | - | - | - |
| S9 | S20 | - | - | 0.018 | 0.013 | 0.002 | 0.009 | 0.008 | 0.009 | 0.006 | 0.012 | 0.005 | 0.006 | 0.005 | 0.010 |
| S9 | S21 | - | - | - | - | - | - | <0.001 | - | - | - | - | - | - | - |
| S9 | S22 | - | - | 0.002 | 0.003 | <0.001 | 0.001 | 0.002 | 0.002 | 0.001 | 0.001 | - | - | - | - |
| S9 | S24 | - | - | 0.018 | 0.013 | 0.002 | 0.009 | 0.008 | 0.009 | 0.006 | 0.012 | 0.005 | 0.005 | 0.004 | 0.010 |
| S9 | S25 | - | - | - | - | - | - | - | - | - | - | <0.001 | 0.001 | <0.001 | 0.001 |
| S9 | S26 | - | - | - | - | - | - | - | <0.001 | - | - | - | - | - | - |
| S9 | S27 | - | - | 0.112 | 0.155 | 0.156 | 0.110 | 0.170 | 0.143 | 0.120 | 0.153 | 0.070 | 0.182 | 0.013 | 0.178 |
| S9 | S28 | - | - | 1.000 | 0.837 | 1.000 | 0.929 | 0.937 | 0.937 | 0.790 | 0.929 | 1.000 | 0.870 | 0.188 | 0.638 |
| S9 | S29 | - | - | 0.084 | 0.114 | 0.156 | 0.106 | 0.166 | 0.138 | 0.120 | 0.150 | 0.068 | 0.189 | 0.043 | 0.226 |
| S10 | S11 | 0.003 | 0.001 | - | - | - | - | - | <0.001 | - | - | - | - | - | - |
| S10 | S12 | 0.007 | 0.005 | - | - | - | - | <0.001 | <0.001 | - | - | - | - | - | - |
| S10 | S13 | 0.007 | 0.001 | - | - | - | - | - | <0.001 | - | <0.001 | - | - | - | - |
| S10 | S14 | - | - | 0.002 | 0.003 | <0.001 | 0.002 | 0.002 | 0.002 | 0.001 | 0.001 | - | - | - | - |
| S10 | S15 | - | - | 0.001 | 0.005 | <0.001 | - | 0.003 | 0.002 | 0.001 | 0.003 | 0.001 | 0.003 | 0.002 | 0.004 |
| S10 | S16 | - | - | 0.002 | 0.003 | <0.001 | 0.002 | 0.002 | 0.002 | 0.001 | 0.001 | - | - | - | - |
| S10 | S17 | - | - | 0.002 | 0.003 | <0.001 | 0.002 | 0.002 | 0.002 | 0.001 | 0.001 | - | - | - | - |
| S10 | S18 | 0.007 | 0.001 | - | - | - | - | - | - | - | - | - | - | - | - |
| S10 | S19 | 0.108 | - | - | - | - | - | - | - | - | - | - | - | - | - |
| S10 | S20 | 0.014 | 0.013 | 0.018 | 0.014 | 0.002 | 0.011 | 0.008 | 0.009 | 0.006 | 0.012 | 0.005 | 0.006 | 0.005 | 0.010 |
| S10 | S21 | - | 0.002 | - | - | - | - | <0.001 | - | - | - | - | - | - | - |
| S10 | S22 | - | - | 0.002 | 0.003 | <0.001 | 0.002 | 0.002 | 0.002 | 0.001 | 0.001 | - | - | - | - |
| S10 | S23 | - | 0.002 | - | - | - | - | - | - | - | - | - | - | - | - |
| S10 | S24 | 0.014 | 0.012 | 0.018 | 0.014 | 0.002 | 0.011 | 0.009 | 0.009 | 0.006 | 0.012 | 0.005 | 0.005 | 0.004 | 0.010 |
| S10 | S25 | - | - | - | - | - | - | - | - | - | - | <0.001 | 0.001 | <0.001 | 0.001 |
| S10 | S26 | - | - | - | - | - | - | - | <0.001 | - | - | - | - | - | - |
| S10 | S27 | 0.413 | 0.051 | 0.112 | 0.175 | 0.156 | 0.100 | 0.181 | 0.143 | 0.120 | 0.153 | 0.070 | 0.182 | 0.013 | 0.178 |
| S10 | S28 | 1.000 | 0.490 | 1.000 | 0.945 | 1.000 | 0.722 | 1.000 | 0.937 | 0.790 | 0.929 | 1.000 | 0.870 | 0.188 | 0.638 |
| S10 | S29 | 0.413 | 0.051 | 0.084 | 0.131 | 0.156 | 0.096 | 0.177 | 0.138 | 0.120 | 0.150 | 0.068 | 0.189 | 0.043 | 0.226 |
| S11 | S12 | 0.001 | 0.003 | - | - | - | - | - | <0.001 | - | - | - | - | - | - |
| S11 | S13 | 0.001 | <0.001 | - | - | - | - | - | <0.001 | - | - | - | - | - | - |
| S11 | S14 | - | - | - | - | - | - | - | <0.001 | - | - | - | - | - | - |
| S11 | S15 | - | - | - | - | - | - | - | <0.001 | - | - | - | - | - | - |
| S11 | S16 | - | - | - | - | - | - | - | <0.001 | - | - | - | - | - | - |
| S11 | S17 | - | - | - | - | - | - | - | <0.001 | - | - | - | - | - | - |
| S11 | S18 | 0.001 | <0.001 | - | - | - | - | - | - | - | - | - | - | - | - |
| S11 | S19 | <0.001 | - | - | - | - | - | - | - | - | - | - | - | - | - |
| S11 | S20 | 0.002 | 0.007 | - | - | - | - | - | 0.001 | - | - | - | - | - | - |
| S11 | S21 | - | 0.001 | - | - | - | - | - | - | - | - | - | - | - | - |
| S11 | S22 | - | - | - | - | - | - | - | <0.001 | - | - | - | - | - | - |
| S11 | S23 | - | 0.001 | - | - | - | - | - | - | - | - | - | - | - | - |
| S11 | S24 | 0.002 | 0.006 | - | - | - | - | - | 0.001 | - | - | - | - | - | - |
| S11 | S26 | - | - | - | - | - | - | - | <0.001 | - | - | - | - | - | - |
| S11 | S27 | 0.008 | 0.016 | - | - | - | - | - | 0.002 | - | - | - | - | - | - |
| S11 | S28 | 0.003 | <0.001 | - | - | - | - | - | <0.001 | - | - | - | - | - | - |
| S11 | S29 | 0.008 | 0.016 | - | - | - | - | - | 0.002 | - | - | - | - | - | - |
| S12 | S13 | 0.001 | 0.003 | - | - | - | - | - | <0.001 | - | - | - | - | - | - |
| S12 | S14 | - | - | - | - | - | - | <0.001 | <0.001 | - | - | - | - | - | - |
| S12 | S15 | - | - | - | - | - | - | <0.001 | <0.001 | - | - | - | - | - | - |
| S12 | S16 | - | - | - | - | - | - | <0.001 | <0.001 | - | - | - | - | - | - |
| S12 | S17 | - | - | - | - | - | - | <0.001 | <0.001 | - | - | - | - | - | - |
| S12 | S18 | 0.001 | 0.003 | - | - | - | - | - | - | - | - | - | - | - | - |
| S12 | S19 | 0.001 | - | - | - | - | - | - | - | - | - | - | - | - | - |
| S12 | S20 | 0.003 | 0.044 | - | - | - | - | <0.001 | 0.001 | - | - | - | - | - | - |
| S12 | S21 | - | 0.005 | - | - | - | - | <0.001 | - | - | - | - | - | - | - |
| S12 | S22 | - | - | - | - | - | - | <0.001 | <0.001 | - | - | - | - | - | - |
| S12 | S23 | - | 0.005 | - | - | - | - | - | - | - | - | - | - | - | - |
| S12 | S24 | 0.003 | 0.053 | - | - | - | - | <0.001 | 0.001 | - | - | - | - | - | - |
| S12 | S26 | - | - | - | - | - | - | - | <0.001 | - | - | - | - | - | - |
| S12 | S27 | 0.016 | 0.002 | - | - | - | - | 0.002 | 0.002 | - | - | - | - | - | - |
| S12 | S28 | 0.007 | 0.003 | - | - | - | - | <0.001 | <0.001 | - | - | - | - | - | - |
| S12 | S29 | 0.016 | 0.002 | - | - | - | - | 0.002 | 0.002 | - | - | - | - | - | - |
| S13 | S14 | - | - | - | - | - | - | - | <0.001 | - | <0.001 | - | - | - | - |
| S13 | S15 | - | - | - | - | - | - | - | <0.001 | - | <0.001 | - | - | - | - |
| S13 | S16 | - | - | - | - | - | - | - | <0.001 | - | <0.001 | - | - | - | - |
| S13 | S17 | - | - | - | - | - | - | - | <0.001 | - | <0.001 | - | - | - | - |
| S13 | S18 | 0.001 | <0.001 | - | - | - | - | - | - | - | - | - | - | - | - |
| S13 | S19 | 0.001 | - | - | - | - | - | - | - | - | - | - | - | - | - |
| S13 | S20 | 0.003 | 0.007 | - | - | - | - | - | 0.001 | - | 0.001 | - | - | - | - |
| S13 | S21 | - | 0.001 | - | - | - | - | - | - | - | - | - | - | - | - |
| S13 | S22 | - | - | - | - | - | - | - | <0.001 | - | <0.001 | - | - | - | - |
| S13 | S23 | - | 0.001 | - | - | - | - | - | - | - | - | - | - | - | - |
| S13 | S24 | 0.003 | 0.006 | - | - | - | - | - | 0.001 | - | 0.001 | - | - | - | - |
| S13 | S26 | - | - | - | - | - | - | - | <0.001 | - | - | - | - | - | - |
| S13 | S27 | 0.016 | 0.025 | - | - | - | - | - | 0.008 | - | 0.010 | - | - | - | - |
| S13 | S28 | 0.007 | <0.001 | - | - | - | - | - | <0.001 | - | <0.001 | - | - | - | - |
| S13 | S29 | 0.016 | 0.025 | - | - | - | - | - | 0.008 | - | 0.010 | - | - | - | - |
| S14 | S15 | - | - | 0.663 | 0.146 | 1.000 | - | 0.001 | 0.001 | <0.001 | <0.001 | 0.001 | 0.003 | 0.002 | 0.004 |
| S14 | S16 | - | - | 1.000 | 1.000 | 1.000 | 0.854 | 1.000 | 1.000 | 1.000 | 1.000 | - | - | - | - |
| S14 | S17 | - | - | 1.000 | 1.000 | 1.000 | 0.854 | 1.000 | 1.000 | 1.000 | 1.000 | - | - | - | - |
| S14 | S20 | - | - | 0.004 | 0.006 | 0.001 | 0.004 | 0.003 | 0.004 | 0.001 | 0.002 | 0.005 | 0.006 | 0.005 | 0.010 |
| S14 | S21 | - | - | - | - | - | - | <0.001 | - | - | - | - | - | - | - |
| S14 | S22 | - | - | 1.000 | 1.000 | 1.000 | 0.854 | 1.000 | 1.000 | 1.000 | 1.000 | - | - | - | - |
| S14 | S24 | - | - | 0.004 | 0.006 | 0.001 | 0.004 | 0.003 | 0.004 | 0.001 | 0.002 | 0.005 | 0.005 | 0.004 | 0.010 |
| S14 | S25 | - | - | - | - | - | - | - | - | - | - | <0.001 | 0.001 | <0.001 | 0.001 |
| S14 | S26 | - | - | - | - | - | - | - | <0.001 | - | - | - | - | - | - |
| S14 | S27 | - | - | 0.026 | 0.046 | 0.077 | 0.031 | 0.036 | 0.060 | 0.029 | 0.021 | 0.070 | 0.182 | 0.013 | 0.178 |
| S14 | S28 | - | - | 0.002 | 0.003 | <0.001 | 0.001 | 0.002 | 0.002 | 0.001 | 0.001 | 1.000 | 0.870 | 0.188 | 0.638 |
| S14 | S29 | - | - | 0.005 | 0.062 | 0.077 | 0.048 | 0.060 | 0.058 | 0.029 | 0.020 | 0.068 | 0.189 | 0.043 | 0.226 |
| S15 | S16 | - | - | 0.663 | 0.146 | 1.000 | - | 0.001 | 0.001 | <0.001 | <0.001 | - | - | - | - |
| S15 | S17 | - | - | 0.663 | 0.146 | 1.000 | - | 0.001 | 0.001 | <0.001 | <0.001 | - | - | - | - |
| S15 | S20 | - | - | 0.003 | 0.009 | 0.001 | - | 0.004 | 0.004 | 0.003 | 0.006 | 0.018 | <0.001 | 0.006 | 0.015 |
| S15 | S21 | - | - | - | - | - | - | <0.001 | - | - | - | - | - | - | - |
| S15 | S22 | - | - | 0.663 | 0.146 | 1.000 | - | 0.001 | 0.001 | <0.001 | <0.001 | - | - | - | - |
| S15 | S24 | - | - | 0.003 | 0.009 | 0.001 | - | 0.004 | 0.004 | 0.003 | 0.006 | 0.018 | 0.002 | 0.017 | 0.016 |
| S15 | S25 | - | - | - | - | - | - | - | - | - | - | 0.001 | 0.002 | 0.001 | 0.001 |
| S15 | S26 | - | - | - | - | - | - | - | 0.140 | - | - | - | - | - | - |
| S15 | S27 | - | - | 0.017 | 0.002 | 0.077 | - | 0.005 | 0.013 | 0.009 | 0.018 | 0.020 | 0.006 | 0.020 | 0.002 |
| S15 | S28 | - | - | 0.001 | 0.005 | <0.001 | - | 0.003 | 0.002 | 0.001 | 0.003 | 0.001 | 0.003 | 0.002 | 0.004 |
| S15 | S29 | - | - | <0.001 | <0.001 | 0.077 | - | 0.006 | 0.013 | 0.009 | 0.018 | 0.021 | 0.005 | 0.008 | 0.020 |
| S16 | S17 | - | - | 1.000 | 1.000 | 1.000 | 1.000 | 1.000 | 1.000 | 1.000 | 1.000 | - | - | - | - |
| S16 | S20 | - | - | 0.004 | 0.006 | 0.001 | 0.003 | 0.003 | 0.004 | 0.001 | 0.002 | - | - | - | - |
| S16 | S21 | - | - | - | - | - | - | <0.001 | - | - | - | - | - | - | - |
| S16 | S22 | - | - | 1.000 | 1.000 | 1.000 | 1.000 | 1.000 | 1.000 | 1.000 | 1.000 | - | - | - | - |
| S16 | S24 | - | - | 0.004 | 0.006 | 0.001 | 0.003 | 0.003 | 0.004 | 0.001 | 0.002 | - | - | - | - |
| S16 | S26 | - | - | - | - | - | - | - | <0.001 | - | - | - | - | - | - |
| S16 | S27 | - | - | 0.026 | 0.046 | 0.077 | 0.024 | 0.036 | 0.060 | 0.029 | 0.021 | - | - | - | - |
| S16 | S28 | - | - | 0.002 | 0.003 | <0.001 | 0.001 | 0.002 | 0.002 | 0.001 | 0.001 | - | - | - | - |
| S16 | S29 | - | - | 0.005 | 0.062 | 0.077 | 0.041 | 0.060 | 0.058 | 0.029 | 0.020 | - | - | - | - |
| S17 | S20 | - | - | 0.004 | 0.006 | 0.001 | 0.003 | 0.003 | 0.004 | 0.001 | 0.002 | - | - | - | - |
| S17 | S21 | - | - | - | - | - | - | <0.001 | - | - | - | - | - | - | - |
| S17 | S22 | - | - | 1.000 | 1.000 | 1.000 | 1.000 | 1.000 | 1.000 | 1.000 | 1.000 | - | - | - | - |
| S17 | S24 | - | - | 0.004 | 0.006 | 0.001 | 0.003 | 0.003 | 0.004 | 0.001 | 0.002 | - | - | - | - |
| S17 | S26 | - | - | - | - | - | - | - | <0.001 | - | - | - | - | - | - |
| S17 | S27 | - | - | 0.026 | 0.046 | 0.077 | 0.024 | 0.036 | 0.060 | 0.029 | 0.021 | - | - | - | - |
| S17 | S28 | - | - | 0.002 | 0.003 | <0.001 | 0.001 | 0.002 | 0.002 | 0.001 | 0.001 | - | - | - | - |
| S17 | S29 | - | - | 0.005 | 0.062 | 0.077 | 0.041 | 0.060 | 0.058 | 0.029 | 0.020 | - | - | - | - |
| S18 | S19 | 0.001 | - | - | - | - | - | - | - | - | - | - | - | - | - |
| S18 | S20 | 0.003 | 0.007 | - | - | - | - | - | - | - | - | - | - | - | - |
| S18 | S21 | - | 0.001 | - | - | - | - | - | - | - | - | - | - | - | - |
| S18 | S23 | - | 0.001 | - | - | - | - | - | - | - | - | - | - | - | - |
| S18 | S24 | 0.003 | 0.006 | - | - | - | - | - | - | - | - | - | - | - | - |
| S18 | S27 | 0.016 | 0.016 | - | - | - | - | - | - | - | - | - | - | - | - |
| S18 | S28 | 0.007 | <0.001 | - | - | - | - | - | - | - | - | - | - | - | - |
| S18 | S29 | 0.016 | 0.016 | - | - | - | - | - | - | - | - | - | - | - | - |
| S19 | S20 | 0.002 | - | - | - | - | - | - | - | - | - | - | - | - | - |
| S19 | S24 | 0.002 | - | - | - | - | - | - | - | - | - | - | - | - | - |
| S19 | S27 | 0.045 | - | - | - | - | - | - | - | - | - | - | - | - | - |
| S19 | S28 | 0.108 | - | - | - | - | - | - | - | - | - | - | - | - | - |
| S19 | S29 | 0.045 | - | - | - | - | - | - | - | - | - | - | - | - | - |
| S20 | S21 | - | 0.013 | - | - | - | - | <0.001 | - | - | - | - | - | - | - |
| S20 | S22 | - | - | 0.004 | 0.006 | 0.001 | 0.003 | 0.003 | 0.004 | 0.001 | 0.002 | - | - | - | - |
| S20 | S23 | - | 0.013 | - | - | - | - | - | - | - | - | - | - | - | - |
| S20 | S24 | 1.000 | 0.901 | 1.000 | 1.000 | 1.000 | 1.000 | 0.958 | 1.000 | 1.000 | 1.000 | 0.912 | 0.826 | 0.962 | 0.964 |
| S20 | S25 | - | - | - | - | - | - | - | - | - | - | 0.003 | 0.003 | 0.003 | 0.003 |
| S20 | S26 | - | - | - | - | - | - | - | 0.001 | - | - | - | - | - | - |
| S20 | S27 | 0.190 | 0.312 | 0.246 | 0.246 | 0.238 | 0.288 | 0.287 | 0.267 | 0.323 | 0.323 | 0.806 | 0.558 | 0.366 | 0.323 |
| S20 | S28 | 0.014 | 0.007 | 0.018 | 0.015 | 0.002 | 0.008 | 0.008 | 0.009 | 0.008 | 0.013 | 0.005 | 0.007 | 0.006 | <0.001 |
| S20 | S29 | 0.190 | 0.312 | 0.253 | 0.269 | 0.238 | 0.303 | 0.282 | 0.284 | 0.323 | 0.316 | 0.783 | 0.645 | 0.676 | 0.618 |
| S21 | S22 | - | - | - | - | - | - | <0.001 | - | - | - | - | - | - | - |
| S21 | S23 | - | 0.013 | - | - | - | - | - | - | - | - | - | - | - | - |
| S21 | S24 | - | 0.901 | - | - | - | - | <0.001 | - | - | - | - | - | - | - |
| S21 | S27 | - | 0.312 | - | - | - | - | 0.010 | - | - | - | - | - | - | - |
| S21 | S28 | - | 0.007 | - | - | - | - | <0.001 | - | - | - | - | - | - | - |
| S21 | S29 | - | 0.312 | - | - | - | - | 0.010 | - | - | - | - | - | - | - |
| S22 | S24 | - | - | 0.004 | 0.006 | 0.001 | 0.003 | 0.003 | 0.004 | 0.001 | 0.002 | - | - | - | - |
| S22 | S26 | - | - | - | - | - | - | - | <0.001 | - | - | - | - | - | - |
| S22 | S27 | - | - | 0.026 | 0.046 | 0.077 | 0.024 | 0.036 | 0.060 | 0.029 | 0.021 | - | - | - | - |
| S22 | S28 | - | - | 0.002 | 0.003 | <0.001 | 0.001 | 0.002 | 0.002 | 0.001 | 0.001 | - | - | - | - |
| S22 | S29 | - | - | 0.005 | 0.062 | 0.077 | 0.041 | 0.060 | 0.058 | 0.029 | 0.020 | - | - | - | - |
| S23 | S24 | - | 0.012 | - | - | - | - | - | - | - | - | - | - | - | - |
| S23 | S27 | - | 0.051 | - | - | - | - | - | - | - | - | - | - | - | - |
| S23 | S28 | - | 0.001 | - | - | - | - | - | - | - | - | - | - | - | - |
| S23 | S29 | - | 0.051 | - | - | - | - | - | - | - | - | - | - | - | - |
| S24 | S25 | - | - | - | - | - | - | - | - | - | - | 0.003 | 0.003 | 0.003 | 0.003 |
| S24 | S26 | - | - | - | - | - | - | - | 0.001 | - | - | - | - | - | - |
| S24 | S27 | 0.190 | 0.378 | 0.246 | 0.246 | 0.238 | 0.288 | 0.300 | 0.267 | 0.323 | 0.323 | 0.728 | 0.557 | 0.384 | 0.307 |
| S24 | S28 | 0.014 | 0.006 | 0.018 | 0.015 | 0.002 | 0.008 | 0.009 | 0.009 | 0.008 | 0.013 | 0.005 | 0.006 | 0.006 | <0.001 |
| S24 | S29 | 0.190 | 0.378 | 0.253 | 0.269 | 0.238 | 0.303 | 0.294 | 0.284 | 0.323 | 0.316 | 0.757 | 0.645 | 0.704 | 0.591 |
| S25 | S27 | - | - | - | - | - | - | - | - | - | - | 0.003 | 0.004 | 0.004 | 0.004 |
| S25 | S28 | - | - | - | - | - | - | - | - | - | - | <0.001 | 0.001 | <0.001 | 0.001 |
| S25 | S29 | - | - | - | - | - | - | - | - | - | - | 0.003 | 0.004 | 0.004 | 0.004 |
| S26 | S27 | - | - | - | - | - | - | - | 0.002 | - | - | - | - | - | - |
| S26 | S28 | - | - | - | - | - | - | - | <0.001 | - | - | - | - | - | - |
| S26 | S29 | - | - | - | - | - | - | - | 0.002 | - | - | - | - | - | - |
| S27 | S28 | 0.413 | 0.025 | 0.112 | 0.185 | 0.156 | 0.102 | 0.181 | 0.127 | 0.152 | 0.165 | 0.070 | 0.209 | 0.115 | 0.229 |
| S27 | S29 | 1.000 | 1.000 | 0.922 | 0.856 | 1.000 | 0.939 | 0.980 | 0.966 | 1.000 | 0.935 | 0.972 | 0.891 | 0.524 | 0.570 |
| S28 | S29 | 0.413 | 0.025 | 0.084 | 0.140 | 0.156 | 0.099 | 0.177 | 0.122 | 0.152 | 0.162 | 0.068 | 0.157 | 0.029 | 0.200 |
