# Supplementary material for: A Recombination Hotspot in a Schizophrenia-Associated Region of GABRB2
Source: PLoS One. 2010 Mar 8;5(3):e9547. doi: 10.1371/journal.pone.0009547 (PMC2833194; doi:10.1371/journal.pone.0009547)
Supplement: Table S6 — Pairwise SNP linkage disequilibrium (LD) r∧2 values for N and D groups. (0.64 MB DOC) [file pone.0009547.s008.doc]

**Table S6** Pairwise SNP linkage disequilibrium (LD) *r*^2^ values for N and D groups

| SNP X | SNP Y | AF | |  | GE | |  | US | |  | JP | |
| --- | --- | --- | --- | --- | --- | --- | --- | --- | --- | --- | --- | --- |
|  |  | N | D |  | N | D |  | N | D |  | N | D |
| S1 | S2 | 0.082 | - |  | - | - |  | 0.005 | - |  | 0.177 | - |
| S1 | S3 | 0.259 | - |  | 0.002 | - |  | 0.009 | - |  | 0.184 | 0.184 |
| S1 | S4 | 0.082 | - |  | - | - |  | 0.005 | - |  | 0.177 | - |
| S1 | S5 | - | - |  | - | - |  | - | - |  | - | - |
| S1 | S6 | 0.030 | - |  | 0.046 | - |  | 0.049 | - |  | 0.044 | - |
| S1 | S7 | 0.086 | - |  | 0.075 | - |  | 0.158 | - |  | 0.008 | - |
| S1 | S8 | 0.086 | - |  | 0.075 | - |  | 0.158 | - |  | 0.008 | - |
| S1 | S9 | - | - |  | 0.038 | - |  | 0.049 | - |  | 0.044 | - |
| S1 | S10 | 0.030 | - |  | 0.046 | - |  | 0.049 | - |  | 0.044 | - |
| S1 | S11 | - | - |  | - | - |  | - | - |  | - | - |
| S1 | S12 | 0.158 | - |  | - | - |  | - | - |  | - | - |
| S1 | S13 | 0.011 | - |  | - | - |  | 0.001 | - |  | - | - |
| S1 | S14 | - | - |  | 0.016 | - |  | 0.026 | - |  | - | - |
| S1 | S15 | - | - |  | 0.025 | - |  | 0.016 | - |  | 0.042 | - |
| S1 | S16 | - | - |  | 0.016 | - |  | 0.026 | - |  | - | - |
| S1 | S17 | - | - |  | 0.016 | - |  | 0.026 | - |  | - | - |
| S1 | S18 | - | - |  | - | - |  | - | - |  | - | - |
| S1 | S19 | - | - |  | - | - |  | - | - |  | - | - |
| S1 | S20 | 1.000 | - |  | 0.402 | - |  | 0.544 | - |  | 0.893 | 0.823 |
| S1 | S21 | 0.009 | - |  | - | - |  | - | - |  | - | - |
| S1 | S22 | - | - |  | 0.016 | - |  | 0.026 | - |  | - | - |
| S1 | S23 | 0.032 | - |  | - | - |  | - | - |  | - | - |
| S1 | S24 | 1.000 | - |  | 0.402 | - |  | 0.544 | - |  | 0.915 | 0.509 |
| S1 | S25 | - | - |  | - | - |  | - | - |  | - | 0.001 |
| S1 | S26 | - | - |  | - | - |  | 0.001 | - |  | - | - |
| S1 | S27 | 0.259 | - |  | 0.042 | - |  | 0.006 | - |  | 0.466 | 0.490 |
| S1 | S28 | 0.016 | - |  | 0.046 | - |  | 0.049 | - |  | 0.044 | <0.001 |
| S1 | S29 | 0.259 | - |  | 0.032 | - |  | 0.031 | - |  | 0.540 | 0.936 |
| S2 | S3 | 0.521 | - |  | - | - |  | 0.529 | - |  | 0.963 | - |
| S2 | S4 | 1.000 | - |  | - | - |  | 1.000 | - |  | 1.000 | - |
| S2 | S5 | - | - |  | - | - |  | - | - |  | - | - |
| S2 | S6 | 0.026 | - |  | - | - |  | 0.005 | - |  | 0.061 | - |
| S2 | S7 | 0.073 | - |  | - | - |  | 0.014 | - |  | 0.011 | - |
| S2 | S8 | 0.073 | - |  | - | - |  | 0.014 | - |  | 0.011 | - |
| S2 | S9 | - | - |  | - | - |  | 0.005 | - |  | 0.061 | - |
| S2 | S10 | 0.026 | - |  | - | - |  | 0.005 | - |  | 0.061 | - |
| S2 | S11 | - | - |  | - | - |  | - | - |  | - | - |
| S2 | S12 | 0.032 | - |  | - | - |  | - | - |  | - | - |
| S2 | S13 | 0.009 | - |  | - | - |  | <0.001 | - |  | - | - |
| S2 | S14 | - | - |  | - | - |  | 0.002 | - |  | - | - |
| S2 | S15 | - | - |  | - | - |  | 0.001 | - |  | 0.134 | - |
| S2 | S16 | - | - |  | - | - |  | 0.002 | - |  | - | - |
| S2 | S17 | - | - |  | - | - |  | 0.002 | - |  | - | - |
| S2 | S18 | - | - |  | - | - |  | - | - |  | - | - |
| S2 | S19 | - | - |  | - | - |  | - | - |  | - | - |
| S2 | S20 | 0.082 | - |  | - | - |  | 0.008 | - |  | 0.198 | - |
| S2 | S21 | 0.007 | - |  | - | - |  | - | - |  | - | - |
| S2 | S22 | - | - |  | - | - |  | 0.002 | - |  | - | - |
| S2 | S23 | 0.027 | - |  | - | - |  | - | - |  | - | - |
| S2 | S24 | 0.082 | - |  | - | - |  | 0.008 | - |  | 0.167 | - |
| S2 | S25 | - | - |  | - | - |  | - | - |  | - | - |
| S2 | S26 | - | - |  | - | - |  | <0.001 | - |  | - | - |
| S2 | S27 | 0.521 | - |  | - | - |  | 0.132 | - |  | 0.305 | - |
| S2 | S28 | 0.014 | - |  | - | - |  | 0.005 | - |  | 0.061 | - |
| S2 | S29 | 0.521 | - |  | - | - |  | 0.150 | - |  | 0.328 | - |
| S3 | S4 | 0.521 | - |  | - | - |  | 0.529 | - |  | 0.963 | - |
| S3 | S5 | - | - |  | - | - |  | - | - |  | - | - |
| S3 | S6 | 0.033 | - |  | 0.003 | - |  | 0.009 | - |  | 0.063 | - |
| S3 | S7 | 0.094 | - |  | 0.008 | - |  | 0.027 | - |  | 0.012 | - |
| S3 | S8 | 0.094 | - |  | 0.008 | - |  | 0.027 | - |  | 0.012 | - |
| S3 | S9 | - | - |  | 0.003 | - |  | 0.009 | - |  | 0.063 | - |
| S3 | S10 | 0.033 | - |  | 0.003 | - |  | 0.009 | - |  | 0.063 | - |
| S3 | S11 | - | - |  | - | - |  | - | - |  | - | - |
| S3 | S12 | 0.041 | - |  | - | - |  | - | - |  | - | - |
| S3 | S13 | 0.012 | - |  | - | - |  | <0.001 | - |  | - | - |
| S3 | S14 | - | - |  | 0.001 | - |  | 0.004 | - |  | - | - |
| S3 | S15 | - | - |  | 0.077 | - |  | 0.003 | - |  | 0.139 | - |
| S3 | S16 | - | - |  | 0.001 | - |  | 0.004 | - |  | - | - |
| S3 | S17 | - | - |  | 0.001 | - |  | 0.004 | - |  | - | - |
| S3 | S18 | - | - |  | - | - |  | - | - |  | - | - |
| S3 | S19 | - | - |  | - | - |  | - | - |  | - | - |
| S3 | S20 | 0.259 | - |  | 0.005 | - |  | 0.016 | - |  | 0.206 | 0.173 |
| S3 | S21 | 0.009 | - |  | - | - |  | - | - |  | - | - |
| S3 | S22 | - | - |  | 0.001 | - |  | 0.004 | - |  | - | - |
| S3 | S23 | 0.035 | - |  | - | - |  | - | - |  | - | - |
| S3 | S24 | 0.259 | - |  | 0.005 | - |  | 0.016 | - |  | 0.173 | 0.107 |
| S3 | S25 | - | - |  | - | - |  | - | - |  | - | <0.001 |
| S3 | S26 | - | - |  | - | - |  | <0.001 | - |  | - | - |
| S3 | S27 | 1.000 | - |  | 0.047 | <0.001 |  | 0.249 | - |  | 0.317 | 0.103 |
| S3 | S28 | 0.018 | - |  | 0.003 | <0.001 |  | 0.009 | - |  | 0.063 | <0.001 |
| S3 | S29 | 1.000 | - |  | 0.061 | <0.001 |  | 0.283 | - |  | 0.340 | 0.196 |
| S4 | S5 | - | - |  | - | - |  | - | - |  | - | - |
| S4 | S6 | 0.026 | - |  | - | - |  | 0.005 | - |  | 0.061 | - |
| S4 | S7 | 0.073 | - |  | - | - |  | 0.014 | - |  | 0.011 | - |
| S4 | S8 | 0.073 | - |  | - | - |  | 0.014 | - |  | 0.011 | - |
| S4 | S9 | - | - |  | - | - |  | 0.005 | - |  | 0.061 | - |
| S4 | S10 | 0.026 | - |  | - | - |  | 0.005 | - |  | 0.061 | - |
| S4 | S11 | - | - |  | - | - |  | - | - |  | - | - |
| S4 | S12 | 0.032 | - |  | - | - |  | - | - |  | - | - |
| S4 | S13 | 0.009 | - |  | - | - |  | <0.001 | - |  | - | - |
| S4 | S14 | - | - |  | - | - |  | 0.002 | - |  | - | - |
| S4 | S15 | - | - |  | - | - |  | 0.001 | - |  | 0.134 | - |
| S4 | S16 | - | - |  | - | - |  | 0.002 | - |  | - | - |
| S4 | S17 | - | - |  | - | - |  | 0.002 | - |  | - | - |
| S4 | S18 | - | - |  | - | - |  | - | - |  | - | - |
| S4 | S19 | - | - |  | - | - |  | - | - |  | - | - |
| S4 | S20 | 0.082 | - |  | - | - |  | 0.008 | - |  | 0.198 | - |
| S4 | S21 | 0.007 | - |  | - | - |  | - | - |  | - | - |
| S4 | S22 | - | - |  | - | - |  | 0.002 | - |  | - | - |
| S4 | S23 | 0.027 | - |  | - | - |  | - | - |  | - | - |
| S4 | S24 | 0.082 | - |  | - | - |  | 0.008 | - |  | 0.167 | - |
| S4 | S25 | - | - |  | - | - |  | - | - |  | - | - |
| S4 | S26 | - | - |  | - | - |  | <0.001 | - |  | - | - |
| S4 | S27 | 0.521 | - |  | - | - |  | 0.132 | - |  | 0.305 | - |
| S4 | S28 | 0.014 | - |  | - | - |  | 0.005 | - |  | 0.061 | - |
| S4 | S29 | 0.521 | - |  | - | - |  | 0.150 | - |  | 0.328 | - |
| S5 | S6 | - | - |  | - | - |  | - | - |  | - | - |
| S5 | S7 | - | - |  | - | - |  | - | - |  | - | - |
| S5 | S8 | - | - |  | - | - |  | - | - |  | - | - |
| S5 | S9 | - | - |  | - | - |  | - | - |  | - | - |
| S5 | S10 | - | - |  | - | - |  | - | - |  | - | - |
| S5 | S11 | - | - |  | - | - |  | - | - |  | - | - |
| S5 | S12 | - | - |  | - | - |  | - | - |  | - | - |
| S5 | S13 | - | - |  | - | - |  | - | - |  | - | - |
| S5 | S14 | - | - |  | - | - |  | - | - |  | - | - |
| S5 | S15 | - | - |  | - | - |  | - | - |  | - | - |
| S5 | S16 | - | - |  | - | - |  | - | - |  | - | - |
| S5 | S17 | - | - |  | - | - |  | - | - |  | - | - |
| S5 | S18 | - | - |  | - | - |  | - | - |  | - | - |
| S5 | S19 | - | - |  | - | - |  | - | - |  | - | - |
| S5 | S20 | - | - |  | - | - |  | - | - |  | - | - |
| S5 | S21 | - | - |  | - | - |  | - | - |  | - | - |
| S5 | S22 | - | - |  | - | - |  | - | - |  | - | - |
| S5 | S23 | - | - |  | - | - |  | - | - |  | - | - |
| S5 | S24 | - | - |  | - | - |  | - | - |  | - | - |
| S5 | S25 | - | - |  | - | - |  | - | - |  | - | - |
| S5 | S26 | - | - |  | - | - |  | - | - |  | - | - |
| S5 | S27 | - | - |  | - | - |  | - | - |  | - | - |
| S5 | S28 | - | - |  | - | - |  | - | - |  | - | - |
| S5 | S29 | - | - |  | - | - |  | - | - |  | - | - |
| S6 | S7 | 0.011 | - |  | 0.188 | - |  | 0.155 | - |  | 0.003 | - |
| S6 | S8 | 0.011 | - |  | 0.188 | - |  | 0.155 | - |  | 0.003 | - |
| S6 | S9 | - | - |  | 0.818 | - |  | 1.000 | - |  | 1.000 | - |
| S6 | S10 | 1.000 | - |  | 1.000 | - |  | 1.000 | - |  | 1.000 | - |
| S6 | S11 | - | - |  | - | - |  | - | - |  | - | - |
| S6 | S12 | 0.005 | - |  | - | - |  | - | - |  | - | - |
| S6 | S13 | 0.001 | - |  | - | - |  | 0.001 | - |  | - | - |
| S6 | S14 | - | - |  | 0.026 | - |  | 0.025 | - |  | - | - |
| S6 | S15 | - | - |  | 0.041 | - |  | 0.016 | - |  | 0.034 | - |
| S6 | S16 | - | - |  | 0.026 | - |  | 0.025 | - |  | - | - |
| S6 | S17 | - | - |  | 0.026 | - |  | 0.025 | - |  | - | - |
| S6 | S18 | - | - |  | - | - |  | - | - |  | - | - |
| S6 | S19 | - | - |  | - | - |  | - | - |  | - | - |
| S6 | S20 | 0.030 | - |  | 0.115 | - |  | 0.091 | - |  | 0.050 | - |
| S6 | S21 | 0.001 | - |  | - | - |  | - | - |  | - | - |
| S6 | S22 | - | - |  | 0.026 | - |  | 0.025 | - |  | - | - |
| S6 | S23 | 0.004 | - |  | - | - |  | - | - |  | - | - |
| S6 | S24 | 0.030 | - |  | 0.115 | - |  | 0.091 | - |  | 0.042 | - |
| S6 | S25 | - | - |  | - | - |  | - | - |  | - | - |
| S6 | S26 | - | - |  | - | - |  | 0.001 | - |  | - | - |
| S6 | S27 | 0.033 | - |  | 0.068 | - |  | 0.034 | - |  | 0.199 | - |
| S6 | S28 | 0.535 | - |  | 1.000 | - |  | 1.000 | - |  | 1.000 | - |
| S6 | S29 | 0.033 | - |  | 0.034 | - |  | 0.030 | - |  | 0.185 | - |
| S7 | S8 | 1.000 | - |  | 1.000 | - |  | 1.000 | - |  | 1.000 | - |
| S7 | S9 | - | - |  | 0.154 | - |  | 0.155 | - |  | 0.003 | - |
| S7 | S10 | 0.011 | - |  | 0.188 | - |  | 0.155 | - |  | 0.003 | - |
| S7 | S11 | - | - |  | - | - |  | - | - |  | - | - |
| S7 | S12 | 0.014 | - |  | - | - |  | - | - |  | - | - |
| S7 | S13 | 0.127 | - |  | - | - |  | 0.008 | - |  | - | - |
| S7 | S14 | - | - |  | 0.065 | - |  | 0.081 | - |  | - | - |
| S7 | S15 | - | - |  | 0.086 | - |  | 0.050 | - |  | 0.006 | - |
| S7 | S16 | - | - |  | 0.065 | - |  | 0.081 | - |  | - | - |
| S7 | S17 | - | - |  | 0.065 | - |  | 0.081 | - |  | - | - |
| S7 | S18 | - | - |  | - | - |  | - | - |  | - | - |
| S7 | S19 | - | - |  | - | - |  | - | - |  | - | - |
| S7 | S20 | 0.086 | - |  | 0.022 | - |  | 0.003 | - |  | 0.009 | - |
| S7 | S21 | 0.003 | - |  | - | - |  | - | - |  | - | - |
| S7 | S22 | - | - |  | 0.065 | - |  | 0.081 | - |  | - | - |
| S7 | S23 | 0.012 | - |  | - | - |  | - | - |  | - | - |
| S7 | S24 | 0.086 | - |  | 0.022 | - |  | 0.003 | - |  | 0.008 | - |
| S7 | S25 | - | - |  | - | - |  | - | - |  | - | - |
| S7 | S26 | - | - |  | - | - |  | 0.004 | - |  | - | - |
| S7 | S27 | 0.094 | - |  | 0.063 | - |  | 0.109 | - |  | 0.037 | - |
| S7 | S28 | 0.006 | - |  | 0.188 | - |  | 0.155 | - |  | 0.003 | - |
| S7 | S29 | 0.094 | - |  | 0.113 | - |  | 0.096 | - |  | 0.035 | - |
| S8 | S9 | - | - |  | 0.154 | - |  | 0.155 | - |  | 0.003 | - |
| S8 | S10 | 0.011 | - |  | 0.188 | - |  | 0.155 | - |  | 0.003 | - |
| S8 | S11 | - | - |  | - | - |  | - | - |  | - | - |
| S8 | S12 | 0.014 | - |  | - | - |  | - | - |  | - | - |
| S8 | S13 | 0.127 | - |  | - | - |  | 0.008 | - |  | - | - |
| S8 | S14 | - | - |  | 0.065 | - |  | 0.081 | - |  | - | - |
| S8 | S15 | - | - |  | 0.086 | - |  | 0.050 | - |  | 0.006 | - |
| S8 | S16 | - | - |  | 0.065 | - |  | 0.081 | - |  | - | - |
| S8 | S17 | - | - |  | 0.065 | - |  | 0.081 | - |  | - | - |
| S8 | S18 | - | - |  | - | - |  | - | - |  | - | - |
| S8 | S19 | - | - |  | - | - |  | - | - |  | - | - |
| S8 | S20 | 0.086 | - |  | 0.022 | - |  | 0.003 | - |  | 0.009 | - |
| S8 | S21 | 0.003 | - |  | - | - |  | - | - |  | - | - |
| S8 | S22 | - | - |  | 0.065 | - |  | 0.081 | - |  | - | - |
| S8 | S23 | 0.012 | - |  | - | - |  | - | - |  | - | - |
| S8 | S24 | 0.086 | - |  | 0.022 | - |  | 0.003 | - |  | 0.008 | - |
| S8 | S25 | - | - |  | - | - |  | - | - |  | - | - |
| S8 | S26 | - | - |  | - | - |  | 0.004 | - |  | - | - |
| S8 | S27 | 0.094 | - |  | 0.063 | - |  | 0.109 | - |  | 0.037 | - |
| S8 | S28 | 0.006 | - |  | 0.188 | - |  | 0.155 | - |  | 0.003 | - |
| S8 | S29 | 0.094 | - |  | 0.113 | - |  | 0.096 | - |  | 0.035 | - |
| S9 | S10 | - | - |  | 0.818 | - |  | 1.000 | - |  | 1.000 | - |
| S9 | S11 | - | - |  | - | - |  | - | - |  | - | - |
| S9 | S12 | - | - |  | - | - |  | - | - |  | - | - |
| S9 | S13 | - | - |  | - | - |  | 0.001 | - |  | - | - |
| S9 | S14 | - | - |  | 0.021 | - |  | 0.025 | - |  | - | - |
| S9 | S15 | - | - |  | 0.034 | - |  | 0.016 | - |  | 0.034 | - |
| S9 | S16 | - | - |  | 0.021 | - |  | 0.025 | - |  | - | - |
| S9 | S17 | - | - |  | 0.021 | - |  | 0.025 | - |  | - | - |
| S9 | S18 | - | - |  | - | - |  | - | - |  | - | - |
| S9 | S19 | - | - |  | - | - |  | - | - |  | - | - |
| S9 | S20 | - | - |  | 0.094 | - |  | 0.091 | - |  | 0.050 | - |
| S9 | S21 | - | - |  | - | - |  | - | - |  | - | - |
| S9 | S22 | - | - |  | 0.021 | - |  | 0.025 | - |  | - | - |
| S9 | S23 | - | - |  | - | - |  | - | - |  | - | - |
| S9 | S24 | - | - |  | 0.094 | - |  | 0.091 | - |  | 0.042 | - |
| S9 | S25 | - | - |  | - | - |  | - | - |  | - | - |
| S9 | S26 | - | - |  | - | - |  | 0.001 | - |  | - | - |
| S9 | S27 | - | - |  | 0.056 | - |  | 0.034 | - |  | 0.199 | - |
| S9 | S28 | - | - |  | 0.818 | - |  | 1.000 | - |  | 1.000 | - |
| S9 | S29 | - | - |  | 0.025 | - |  | 0.030 | - |  | 0.185 | - |
| S10 | S11 | - | - |  | - | - |  | - | - |  | - | - |
| S10 | S12 | 0.005 | - |  | - | - |  | - | - |  | - | - |
| S10 | S13 | 0.001 | - |  | - | - |  | 0.001 | - |  | - | - |
| S10 | S14 | - | - |  | 0.026 | - |  | 0.025 | - |  | - | - |
| S10 | S15 | - | - |  | 0.041 | - |  | 0.016 | - |  | 0.034 | - |
| S10 | S16 | - | - |  | 0.026 | - |  | 0.025 | - |  | - | - |
| S10 | S17 | - | - |  | 0.026 | - |  | 0.025 | - |  | - | - |
| S10 | S18 | - | - |  | - | - |  | - | - |  | - | - |
| S10 | S19 | - | - |  | - | - |  | - | - |  | - | - |
| S10 | S20 | 0.030 | - |  | 0.115 | - |  | 0.091 | - |  | 0.050 | - |
| S10 | S21 | 0.001 | - |  | - | - |  | - | - |  | - | - |
| S10 | S22 | - | - |  | 0.026 | - |  | 0.025 | - |  | - | - |
| S10 | S23 | 0.004 | - |  | - | - |  | - | - |  | - | - |
| S10 | S24 | 0.030 | - |  | 0.115 | - |  | 0.091 | - |  | 0.042 | - |
| S10 | S25 | - | - |  | - | - |  | - | - |  | - | - |
| S10 | S26 | - | - |  | - | - |  | 0.001 | - |  | - | - |
| S10 | S27 | 0.033 | - |  | 0.068 | - |  | 0.034 | - |  | 0.199 | - |
| S10 | S28 | 0.535 | - |  | 1.000 | - |  | 1.000 | - |  | 1.000 | - |
| S10 | S29 | 0.033 | - |  | 0.034 | - |  | 0.030 | - |  | 0.185 | - |
| S11 | S12 | - | 0.026 |  | - | - |  | - | <0.001 |  | - | - |
| S11 | S13 | - | - |  | - | - |  | - | - |  | - | - |
| S11 | S14 | - | - |  | - | - |  | - | - |  | - | - |
| S11 | S15 | - | - |  | - | - |  | - | - |  | - | - |
| S11 | S16 | - | - |  | - | - |  | - | - |  | - | - |
| S11 | S17 | - | - |  | - | - |  | - | - |  | - | - |
| S11 | S18 | - | 0.007 |  | - | - |  | - | - |  | - | - |
| S11 | S19 | - | - |  | - | - |  | - | - |  | - | - |
| S11 | S20 | - | 0.007 |  | - | - |  | - | - |  | - | - |
| S11 | S21 | - | - |  | - | - |  | - | - |  | - | - |
| S11 | S22 | - | - |  | - | - |  | - | - |  | - | - |
| S11 | S23 | - | - |  | - | - |  | - | - |  | - | - |
| S11 | S24 | - | - |  | - | - |  | - | - |  | - | - |
| S11 | S25 | - | - |  | - | - |  | - | - |  | - | - |
| S11 | S26 | - | - |  | - | - |  | - | - |  | - | - |
| S11 | S27 | - | - |  | - | - |  | - | <0.001 |  | - | - |
| S11 | S28 | - | - |  | - | - |  | - | <0.001 |  | - | - |
| S11 | S29 | - | - |  | - | - |  | - | <0.001 |  | - | - |
| S12 | S13 | 0.002 | - |  | - | - |  | - | - |  | - | - |
| S12 | S14 | - | - |  | - | - |  | - | - |  | - | - |
| S12 | S15 | - | - |  | - | - |  | - | - |  | - | - |
| S12 | S16 | - | - |  | - | - |  | - | - |  | - | - |
| S12 | S17 | - | - |  | - | - |  | - | - |  | - | - |
| S12 | S18 | - | 0.010 |  | - | - |  | - | - |  | - | - |
| S12 | S19 | - | - |  | - | - |  | - | - |  | - | - |
| S12 | S20 | 0.158 | 0.010 |  | - | - |  | - | - |  | - | - |
| S12 | S21 | 0.001 | - |  | - | - |  | - | - |  | - | - |
| S12 | S22 | - | - |  | - | - |  | - | - |  | - | - |
| S12 | S23 | 0.005 | - |  | - | - |  | - | - |  | - | - |
| S12 | S24 | 0.158 | - |  | - | - |  | - | - |  | - | - |
| S12 | S25 | - | - |  | - | - |  | - | - |  | - | - |
| S12 | S26 | - | - |  | - | - |  | - | - |  | - | - |
| S12 | S27 | 0.041 | - |  | - | - |  | - | <0.001 |  | - | - |
| S12 | S28 | 0.003 | - |  | - | - |  | - | <0.001 |  | - | - |
| S12 | S29 | 0.041 | - |  | - | - |  | - | <0.001 |  | - | - |
| S13 | S14 | - | - |  | - | - |  | <0.001 | - |  | - | - |
| S13 | S15 | - | - |  | - | - |  | <0.001 | - |  | - | - |
| S13 | S16 | - | - |  | - | - |  | <0.001 | - |  | - | - |
| S13 | S17 | - | - |  | - | - |  | <0.001 | - |  | - | - |
| S13 | S18 | - | - |  | - | - |  | - | - |  | - | - |
| S13 | S19 | - | - |  | - | - |  | - | - |  | - | - |
| S13 | S20 | 0.011 | - |  | - | - |  | 0.002 | - |  | - | - |
| S13 | S21 | <0.001 | - |  | - | - |  | - | - |  | - | - |
| S13 | S22 | - | - |  | - | - |  | <0.001 | - |  | - | - |
| S13 | S23 | 0.001 | - |  | - | - |  | - | - |  | - | - |
| S13 | S24 | 0.011 | - |  | - | - |  | 0.002 | - |  | - | - |
| S13 | S25 | - | - |  | - | - |  | - | - |  | - | - |
| S13 | S26 | - | - |  | - | - |  | <0.001 | - |  | - | - |
| S13 | S27 | 0.012 | - |  | - | - |  | <0.001 | - |  | - | - |
| S13 | S28 | <0.001 | - |  | - | - |  | 0.001 | - |  | - | - |
| S13 | S29 | 0.012 | - |  | - | - |  | <0.001 | - |  | - | - |
| S14 | S15 | - | - |  | 0.099 | - |  | 0.008 | - |  | - | - |
| S14 | S16 | - | - |  | 1.000 | - |  | 1.000 | - |  | - | - |
| S14 | S17 | - | - |  | 1.000 | - |  | 1.000 | - |  | - | - |
| S14 | S18 | - | - |  | - | - |  | - | - |  | - | - |
| S14 | S19 | - | - |  | - | - |  | - | - |  | - | - |
| S14 | S20 | - | - |  | 0.040 | - |  | 0.047 | - |  | - | - |
| S14 | S21 | - | - |  | - | - |  | - | - |  | - | - |
| S14 | S22 | - | - |  | 1.000 | - |  | 1.000 | - |  | - | - |
| S14 | S23 | - | - |  | - | - |  | - | - |  | - | - |
| S14 | S24 | - | - |  | 0.040 | - |  | 0.047 | - |  | - | - |
| S14 | S25 | - | - |  | - | - |  | - | - |  | - | - |
| S14 | S26 | - | - |  | - | - |  | <0.001 | - |  | - | - |
| S14 | S27 | - | - |  | <0.001 | - |  | 0.018 | - |  | - | - |
| S14 | S28 | - | - |  | 0.026 | - |  | 0.025 | - |  | - | - |
| S14 | S29 | - | - |  | 0.018 | - |  | 0.016 | - |  | - | - |
| S15 | S16 | - | - |  | 0.099 | - |  | 0.008 | - |  | - | - |
| S15 | S17 | - | - |  | 0.099 | - |  | 0.008 | - |  | - | - |
| S15 | S18 | - | - |  | - | - |  | - | - |  | - | - |
| S15 | S19 | - | - |  | - | - |  | - | - |  | - | - |
| S15 | S20 | - | - |  | 0.063 | - |  | 0.029 | - |  | 0.014 | - |
| S15 | S21 | - | - |  | - | - |  | - | - |  | - | - |
| S15 | S22 | - | - |  | 0.099 | - |  | 0.008 | - |  | - | - |
| S15 | S23 | - | - |  | - | - |  | - | - |  | - | - |
| S15 | S24 | - | - |  | 0.063 | - |  | 0.029 | - |  | 0.038 | - |
| S15 | S25 | - | - |  | - | - |  | - | - |  | - | - |
| S15 | S26 | - | - |  | - | - |  | 0.082 | - |  | - | - |
| S15 | S27 | - | - |  | 0.368 | - |  | 0.453 | - |  | 0.098 | - |
| S15 | S28 | - | - |  | 0.041 | - |  | 0.016 | - |  | 0.034 | - |
| S15 | S29 | - | - |  | 0.306 | - |  | 0.516 | - |  | 0.108 | - |
| S16 | S17 | - | - |  | 1.000 | - |  | 1.000 | - |  | - | - |
| S16 | S18 | - | - |  | - | - |  | - | - |  | - | - |
| S16 | S19 | - | - |  | - | - |  | - | - |  | - | - |
| S16 | S20 | - | - |  | 0.040 | - |  | 0.047 | - |  | - | - |
| S16 | S21 | - | - |  | - | - |  | - | - |  | - | - |
| S16 | S22 | - | - |  | 1.000 | - |  | 1.000 | - |  | - | - |
| S16 | S23 | - | - |  | - | - |  | - | - |  | - | - |
| S16 | S24 | - | - |  | 0.040 | - |  | 0.047 | - |  | - | - |
| S16 | S25 | - | - |  | - | - |  | - | - |  | - | - |
| S16 | S26 | - | - |  | - | - |  | <0.001 | - |  | - | - |
| S16 | S27 | - | - |  | <0.001 | - |  | 0.018 | - |  | - | - |
| S16 | S28 | - | - |  | 0.026 | - |  | 0.025 | - |  | - | - |
| S16 | S29 | - | - |  | 0.018 | - |  | 0.016 | - |  | - | - |
| S17 | S18 | - | - |  | - | - |  | - | - |  | - | - |
| S17 | S19 | - | - |  | - | - |  | - | - |  | - | - |
| S17 | S20 | - | - |  | 0.040 | - |  | 0.047 | - |  | - | - |
| S17 | S21 | - | - |  | - | - |  | - | - |  | - | - |
| S17 | S22 | - | - |  | 1.000 | - |  | 1.000 | - |  | - | - |
| S17 | S23 | - | - |  | - | - |  | - | - |  | - | - |
| S17 | S24 | - | - |  | 0.040 | - |  | 0.047 | - |  | - | - |
| S17 | S25 | - | - |  | - | - |  | - | - |  | - | - |
| S17 | S26 | - | - |  | - | - |  | <0.001 | - |  | - | - |
| S17 | S27 | - | - |  | <0.001 | - |  | 0.018 | - |  | - | - |
| S17 | S28 | - | - |  | 0.026 | - |  | 0.025 | - |  | - | - |
| S17 | S29 | - | - |  | 0.018 | - |  | 0.016 | - |  | - | - |
| S18 | S19 | - | - |  | - | - |  | - | - |  | - | - |
| S18 | S20 | - | 0.003 |  | - | - |  | - | - |  | - | - |
| S18 | S21 | - | - |  | - | - |  | - | - |  | - | - |
| S18 | S22 | - | - |  | - | - |  | - | - |  | - | - |
| S18 | S23 | - | - |  | - | - |  | - | - |  | - | - |
| S18 | S24 | - | - |  | - | - |  | - | - |  | - | - |
| S18 | S25 | - | - |  | - | - |  | - | - |  | - | - |
| S18 | S26 | - | - |  | - | - |  | - | - |  | - | - |
| S18 | S27 | - | - |  | - | - |  | - | - |  | - | - |
| S18 | S28 | - | - |  | - | - |  | - | - |  | - | - |
| S18 | S29 | - | - |  | - | - |  | - | - |  | - | - |
| S19 | S20 | - | - |  | - | - |  | - | - |  | - | - |
| S19 | S21 | - | - |  | - | - |  | - | - |  | - | - |
| S19 | S22 | - | - |  | - | - |  | - | - |  | - | - |
| S19 | S23 | - | - |  | - | - |  | - | - |  | - | - |
| S19 | S24 | - | - |  | - | - |  | - | - |  | - | - |
| S19 | S25 | - | - |  | - | - |  | - | - |  | - | - |
| S19 | S26 | - | - |  | - | - |  | - | - |  | - | - |
| S19 | S27 | - | - |  | - | - |  | - | - |  | - | - |
| S19 | S28 | - | - |  | - | - |  | - | - |  | - | - |
| S19 | S29 | - | - |  | - | - |  | - | - |  | - | - |
| S20 | S21 | 0.009 | - |  | - | - |  | - | - |  | - | - |
| S20 | S22 | - | - |  | 0.040 | - |  | 0.047 | - |  | - | - |
| S20 | S23 | 0.032 | - |  | - | - |  | - | - |  | - | - |
| S20 | S24 | 1.000 | - |  | 1.000 | - |  | 1.000 | - |  | 0.842 | 0.477 |
| S20 | S25 | - | - |  | - | - |  | - | - |  | - | 0.001 |
| S20 | S26 | - | - |  | - | - |  | 0.002 | - |  | - | - |
| S20 | S27 | 0.259 | - |  | 0.030 | - |  | 0.026 | - |  | 0.409 | 0.459 |
| S20 | S28 | 0.016 | - |  | 0.115 | - |  | 0.091 | - |  | 0.050 | <0.001 |
| S20 | S29 | 0.259 | - |  | 0.080 | - |  | 0.056 | - |  | 0.474 | 0.880 |
| S21 | S22 | - | - |  | - | - |  | - | - |  | - | - |
| S21 | S23 | 0.001 | - |  | - | - |  | - | - |  | - | - |
| S21 | S24 | 0.009 | - |  | - | - |  | - | - |  | - | - |
| S21 | S25 | - | - |  | - | - |  | - | - |  | - | - |
| S21 | S26 | - | - |  | - | - |  | - | - |  | - | - |
| S21 | S27 | 0.009 | - |  | - | - |  | - | - |  | - | - |
| S21 | S28 | <0.001 | - |  | - | - |  | - | - |  | - | - |
| S21 | S29 | 0.009 | - |  | - | - |  | - | - |  | - | - |
| S22 | S23 | - | - |  | - | - |  | - | - |  | - | - |
| S22 | S24 | - | - |  | 0.040 | - |  | 0.047 | - |  | - | - |
| S22 | S25 | - | - |  | - | - |  | - | - |  | - | - |
| S22 | S26 | - | - |  | - | - |  | <0.001 | - |  | - | - |
| S22 | S27 | - | - |  | <0.001 | - |  | 0.018 | - |  | - | - |
| S22 | S28 | - | - |  | 0.026 | - |  | 0.025 | - |  | - | - |
| S22 | S29 | - | - |  | 0.018 | - |  | 0.016 | - |  | - | - |
| S23 | S24 | 0.032 | - |  | - | - |  | - | - |  | - | - |
| S23 | S25 | - | - |  | - | - |  | - | - |  | - | - |
| S23 | S26 | - | - |  | - | - |  | - | - |  | - | - |
| S23 | S27 | 0.035 | - |  | - | - |  | - | - |  | - | - |
| S23 | S28 | 0.002 | - |  | - | - |  | - | - |  | - | - |
| S23 | S29 | 0.035 | - |  | - | - |  | - | - |  | - | - |
| S24 | S25 | - | - |  | - | - |  | - | - |  | - | 0.002 |
| S24 | S26 | - | - |  | - | - |  | 0.002 | - |  | - | - |
| S24 | S27 | 0.259 | - |  | 0.030 | - |  | 0.026 | - |  | 0.435 | 0.276 |
| S24 | S28 | 0.016 | - |  | 0.115 | - |  | 0.091 | - |  | 0.042 | <0.001 |
| S24 | S29 | 0.259 | - |  | 0.080 | - |  | 0.056 | - |  | 0.509 | 0.545 |
| S25 | S26 | - | - |  | - | - |  | - | - |  | - | - |
| S25 | S27 | - | - |  | - | - |  | - | - |  | - | 0.002 |
| S25 | S28 | - | - |  | - | - |  | - | - |  | - | <0.001 |
| S25 | S29 | - | - |  | - | - |  | - | - |  | - | 0.001 |
| S26 | S27 | - | - |  | - | - |  | 0.037 | - |  | - | - |
| S26 | S28 | - | - |  | - | - |  | 0.001 | - |  | - | - |
| S26 | S29 | - | - |  | - | - |  | 0.042 | - |  | - | - |
| S27 | S28 | 0.018 | - |  | 0.068 | 1.000 |  | 0.034 | <0.001 |  | 0.199 | 0.207 |
| S27 | S29 | 1.000 | - |  | 0.687 | 0.253 |  | 0.879 | 0.622 |  | 0.932 | 0.525 |
| S28 | S29 | 0.018 | - |  | 0.034 | 0.253 |  | 0.030 | <0.001 |  | 0.185 | <0.001 |
