# Supplementary material for: A Recombination Hotspot in a Schizophrenia-Associated Region of GABRB2
Source: PLoS One. 2010 Mar 8;5(3):e9547. doi: 10.1371/journal.pone.0009547 (PMC2833194; doi:10.1371/journal.pone.0009547)
Supplement: Table S7 — P-value for gender differences and disease association in terms of LD and haplotype frequencies. (1.08 MB DOC) [file pone.0009547.s009.doc]

**Table S7** *P*-value for gender differences and disease association in terms of LD and haplotype frequencies

1. Comparison between female and male groups

| SNP X | SNP Y | AF | | GE | | | | US | | | | JP | | | |
| --- | --- | --- | --- | --- | --- | --- | --- | --- | --- | --- | --- | --- | --- | --- | --- |
|  |  | LD[[1]](#footnote-2) | HF[[2]](#footnote-3) | CN | | SZ | | CN | | SZ | | CN | | SZ | |
|  |  |  |  | LD | HF | LD | HF | LD | HF | LD | HF | LD | HF | LD | HF |
| S1 | S2 | 0.783 | 0.098 | - | - | - | - | - | - | - | - | 0.717 | 0.107 | 0.385 | 0.115 |
| S1 | S3 | 0.618 | 0.092 | 0.215 | 0.508 | 0.602 | 0.001 | 0.552 | 0.837 | 0.993 | 0.309 | 0.662 | 0.115 | 0.175 | 0.748 |
| S1 | S4 | 0.784 | 0.098 | - | - | - | - | - | - | - | - | 0.731 | 0.107 | 0.390 | 0.115 |
| S1 | S5 | 0.434 | 0.029 | 0.233 | 0.587 | 0.602 | 0.001 | 0.665 | 0.714 | 0.952 | 0.360 | 0.980 | 0.115 | 0.092 | 0.023 |
| S1 | S6 | 0.155 | 0.019 | 0.821 | 0.974 | 0.371 | 0.646 | 0.143 | 0.814 | 0.129 | 0.292 | 0.158 | 0.090 | 0.633 | 0.189 |
| S1 | S7 | 0.001 | 0.059 | 0.173 | 0.021 | 0.626 | 0.034 | 0.946 | 0.625 | 0.195 | 0.469 | **0.008[[3]](#footnote-4)** | **0.033** | - | - |
| S1 | S8 | 0.001 | 0.059 | 0.180 | 0.021 | 0.631 | 0.034 | 0.949 | 0.625 | 0.200 | 0.469 | **0.009** | **0.033** | - | - |
| S1 | S9 | - | - | 0.751 | 0.939 | 0.259 | 0.513 | 0.362 | 0.903 | 0.123 | 0.292 | 0.155 | 0.090 | 0.633 | 0.189 |
| S1 | S10 | 0.120 | 0.023 | 0.816 | 0.974 | 0.285 | 0.335 | 0.139 | 0.814 | 0.123 | 0.292 | 0.151 | 0.090 | 0.636 | 0.189 |
| S1 | S11 | 0.762 | 0.156 | - | - | - | - | - | - | - | - | - | - | - | - |
| S1 | S12 | 0.682 | 0.132 | - | - | - | - | 0.502 | 0.657 | - | - | - | - | - | - |
| S1 | S13 | 0.004 | 0.097 | - | - | - | - | - | - | - | - | - | - | - | - |
| S1 | S14 | - | - | 0.453 | 0.665 | 0.287 | 0.640 | 0.789 | 0.883 | 0.357 | 0.654 | - | - | - | - |
| S1 | S15 | - | - | 0.982 | 0.101 | - | - | **0.038** | **0.049** | 0.588 | 0.539 | 0.511 | 0.120 | 0.706 | 0.848 |
| S1 | S16 | - | - | 0.453 | 0.665 | 0.317 | 0.686 | 0.791 | 0.883 | 0.357 | 0.654 | - | - | - | - |
| S1 | S17 | - | - | 0.453 | 0.665 | 0.318 | 0.686 | 0.792 | 0.883 | 0.351 | 0.654 | - | - | - | - |
| S1 | S18 | 0.315 | 0.090 | - | - | - | - | - | - | - | - | - | - | - | - |
| S1 | S20 | 0.608 | 0.030 | 0.806 | 0.400 | 0.104 | 0.049 | 0.794 | 0.859 | 0.358 | 0.198 | 0.960 | 0.086 | 0.187 | 0.294 |
| S1 | S22 | - | - | 0.464 | 0.665 | 0.315 | 0.686 | 0.791 | 0.883 | 0.360 | 0.654 | - | - | - | - |
| S1 | S24 | 0.177 | 0.025 | 0.809 | 0.400 | 0.103 | 0.049 | 0.714 | 0.900 | 0.359 | 0.198 | 0.378 | 0.098 | 0.478 | 0.600 |
| S1 | S25 | - | - | - | - | - | - | - | - | - | - | 0.704 | 0.105 | 0.111 | 0.375 |
| S1 | S27 | 0.616 | 0.092 | 0.196 | 0.103 | 0.623 | 0.019 | 0.678 | 0.775 | 0.936 | 0.427 | 0.379 | 0.225 | 0.915 | 0.924 |
| S1 | S28 | 0.299 | 0.006 | 0.840 | 0.952 | 0.283 | 0.560 | 0.123 | 0.902 | 0.181 | 0.644 | 0.214 | 0.075 | 0.143 | 1.000 |
| S1 | S29 | 0.626 | 0.092 | 0.539 | 0.386 | 0.564 | 0.014 | 0.826 | 0.738 | 0.948 | 0.394 | 0.974 | 0.121 | 0.639 | 0.795 |
| S2 | S3 | 0.606 | 0.333 | - | - | - | - | - | - | - | - | 0.539 | 0.507 | 0.222 | 0.109 |
| S2 | S4 | 1.000 | 0.577 | - | - | - | - | - | - | - | - | 1.000 | 0.669 | 1.000 | 0.038 |
| S2 | S5 | 0.217 | 0.628 | - | - | - | - | - | - | - | - | 0.843 | 0.507 | 0.801 | 0.074 |
| S2 | S6 | 0.020 | 0.093 | - | - | - | - | - | - | - | - | 0.163 | 0.493 | 0.124 | 0.017 |
| S2 | S7 | 0.675 | 0.699 | - | - | - | - | - | - | - | - | 0.314 | 0.670 | - | - |
| S2 | S8 | 0.666 | 0.699 | - | - | - | - | - | - | - | - | 0.308 | 0.670 | - | - |
| S2 | S9 | - | - | - | - | - | - | - | - | - | - | 0.154 | 0.493 | 0.112 | 0.017 |
| S2 | S10 | 0.038 | 0.153 | - | - | - | - | - | - | - | - | 0.157 | 0.493 | 0.113 | 0.017 |
| S2 | S11 | 0.501 | 0.726 | - | - | - | - | - | - | - | - | - | - | - | - |
| S2 | S12 | 0.348 | 0.286 | - | - | - | - | - | - | - | - | - | - | - | - |
| S2 | S13 | 0.366 | 0.856 | - | - | - | - | - | - | - | - | - | - | - | - |
| S2 | S15 | - | - | - | - | - | - | - | - | - | - | 0.947 | 0.865 | 0.044 | 0.083 |
| S2 | S18 | 0.601 | 0.710 | - | - | - | - | - | - | - | - | - | - | - | - |
| S2 | S20 | 0.989 | 0.026 | - | - | - | - | - | - | - | - | 0.542 | 0.275 | 0.460 | 0.141 |
| S2 | S24 | 0.912 | 0.045 | - | - | - | - | - | - | - | - | 0.462 | 0.137 | 0.573 | 0.158 |
| S2 | S25 | - | - | - | - | - | - | - | - | - | - | 0.546 | 0.623 | 0.455 | 0.117 |
| S2 | S27 | 0.606 | 0.333 | - | - | - | - | - | - | - | - | 0.427 | 0.356 | 0.516 | 0.227 |
| S2 | S28 | 0.106 | 0.013 | - | - | - | - | - | - | - | - | 0.113 | 0.368 | 0.195 | 0.033 |
| S2 | S29 | 0.603 | 0.333 | - | - | - | - | - | - | - | - | 0.364 | 0.229 | 0.421 | 0.104 |
| S3 | S4 | 0.608 | 0.333 | - | - | - | - | - | - | - | - | 0.542 | 0.507 | 0.221 | 0.109 |
| S3 | S5 | 0.513 | 0.056 | 0.753 | 0.278 | 1.000 | <0.001 | 0.215 | 0.108 | 0.736 | 0.588 | 0.907 | 0.507 | 0.169 | 0.090 |
| S3 | S6 | **0.004** | **0.002** | 0.722 | 0.442 | 0.110 | 0.001 | 0.253 | 0.754 | 0.507 | 0.628 | 0.033 | 0.257 | 0.015 | 0.152 |
| S3 | S7 | 0.062 | 0.331 | 0.537 | 0.038 | 0.900 | 0.001 | 0.982 | 0.718 | 0.621 | 0.609 | 0.113 | 0.331 | - | - |
| S3 | S8 | 0.066 | 0.331 | 0.542 | 0.038 | 0.897 | 0.001 | 0.982 | 0.718 | 0.614 | 0.609 | 0.109 | 0.331 | - | - |
| S3 | S9 | - | - | 0.910 | 0.508 | 0.241 | 0.001 | 0.418 | 0.822 | 0.504 | 0.628 | 0.031 | 0.257 | 0.014 | 0.152 |
| S3 | S10 | **0.007** | **0.006** | 0.711 | 0.442 | 0.191 | 0.001 | 0.242 | 0.754 | 0.508 | 0.628 | 0.032 | 0.257 | 0.012 | 0.152 |
| S3 | S11 | 0.138 | 0.213 | - | - | - | - | - | - | - | - | - | - | - | - |
| S3 | S12 | 0.696 | 0.208 | - | - | - | - | 0.159 | 0.531 | - | - | - | - | - | - |
| S3 | S13 | 0.130 | 0.325 | - | - | - | - | - | - | - | - | - | - | - | - |
| S3 | S14 | - | - | 0.954 | 0.274 | 0.667 | 0.001 | 0.823 | 0.865 | 0.233 | 0.663 | - | - | - | - |
| S3 | S15 | - | - | 0.767 | 0.080 | - | - | 0.355 | 0.657 | 0.535 | 0.613 | 0.204 | 0.315 | 0.332 | 0.888 |
| S3 | S16 | - | - | 0.950 | 0.274 | 0.658 | 0.001 | 0.817 | 0.865 | 0.237 | 0.663 | - | - | - | - |
| S3 | S17 | - | - | 0.955 | 0.274 | 0.654 | 0.001 | 0.813 | 0.865 | 0.234 | 0.663 | - | - | - | - |
| S3 | S18 | 0.259 | 0.406 | - | - | - | - | - | - | - | - | - | - | - | - |
| S3 | S20 | 0.768 | 0.046 | 0.118 | 0.430 | 0.175 | 0.001 | 0.957 | 0.824 | 0.745 | 0.672 | 0.341 | 0.262 | 0.333 | 0.748 |
| S3 | S22 | - | - | 0.947 | 0.274 | 0.658 | 0.001 | 0.815 | 0.865 | 0.232 | 0.663 | - | - | - | - |
| S3 | S24 | 0.570 | 0.045 | 0.118 | 0.430 | 0.168 | 0.001 | 0.927 | 0.858 | 0.748 | 0.672 | 0.519 | 0.089 | 0.285 | 0.856 |
| S3 | S25 | - | - | - | - | - | - | - | - | - | - | 0.633 | 0.449 | 0.379 | 0.854 |
| S3 | S27 | 0.389 | 0.139 | 0.103 | 0.074 | 0.665 | <0.001 | 0.992 | 0.831 | 0.364 | 0.472 | 0.220 | 0.142 | 0.270 | 0.735 |
| S3 | S28 | **0.003** | **<0.001** | 0.809 | 0.492 | 0.246 | 0.001 | 0.258 | 0.819 | 0.262 | 0.683 | 0.124 | 0.293 | 0.027 | 0.162 |
| S3 | S29 | 1.000 | 0.139 | 0.769 | 0.441 | 0.597 | 0.001 | 0.650 | 0.820 | 0.414 | 0.537 | 0.829 | 0.344 | 0.944 | 0.942 |
| S4 | S5 | 0.217 | 0.628 | - | - | - | - | - | - | - | - | 0.848 | 0.507 | 0.812 | 0.074 |
| S4 | S6 | 0.021 | 0.093 | - | - | - | - | - | - | - | - | 0.158 | 0.493 | 0.118 | 0.017 |
| S4 | S7 | 0.669 | 0.699 | - | - | - | - | - | - | - | - | 0.308 | 0.670 | - | - |
| S4 | S8 | 0.671 | 0.699 | - | - | - | - | - | - | - | - | 0.316 | 0.670 | - | - |
| S4 | S9 | - | - | - | - | - | - | - | - | - | - | 0.155 | 0.493 | 0.122 | 0.017 |
| S4 | S10 | 0.042 | 0.153 | - | - | - | - | - | - | - | - | 0.158 | 0.493 | 0.113 | 0.017 |
| S4 | S11 | 0.502 | 0.726 | - | - | - | - | - | - | - | - | - | - | - | - |
| S4 | S12 | 0.356 | 0.286 | - | - | - | - | - | - | - | - | - | - | - | - |
| S4 | S13 | 0.375 | 0.856 | - | - | - | - | - | - | - | - | - | - | - | - |
| S4 | S15 | - | - | - | - | - | - | - | - | - | - | 0.951 | 0.865 | 0.041 | 0.083 |
| S4 | S18 | 0.594 | 0.710 | - | - | - | - | - | - | - | - | - | - | - | - |
| S4 | S20 | 0.989 | 0.026 | - | - | - | - | - | - | - | - | 0.539 | 0.275 | 0.459 | 0.141 |
| S4 | S24 | 0.918 | 0.045 | - | - | - | - | - | - | - | - | 0.475 | 0.137 | 0.577 | 0.158 |
| S4 | S25 | - | - | - | - | - | - | - | - | - | - | 0.547 | 0.623 | 0.458 | 0.117 |
| 2S4 | S27 | 0.603 | 0.333 | - | - | - | - | - | - | - | - | 0.422 | 0.356 | 0.515 | 0.227 |
| S4 | S28 | 0.104 | 0.013 | - | - | - | - | - | - | - | - | 0.113 | 0.368 | 0.194 | 0.033 |
| S4 | S29 | 0.612 | 0.333 | - | - | - | - | - | - | - | - | 0.360 | 0.229 | 0.422 | 0.104 |
| S5 | S6 | 0.632 | 0.072 | 0.739 | 0.519 | 0.117 | 0.001 | 0.301 | 0.592 | 0.520 | 0.675 | 0.159 | 0.223 | 0.416 | 0.079 |
| S5 | S7 | 0.236 | 0.539 | 0.495 | 0.035 | 0.899 | 0.001 | 0.721 | 0.662 | 0.562 | 0.635 | 0.062 | 0.244 | - | - |
| S5 | S8 | 0.237 | 0.539 | 0.485 | 0.035 | 0.901 | 0.001 | 0.724 | 0.662 | 0.577 | 0.635 | 0.061 | 0.244 | - | - |
| S5 | S9 | - | - | 0.939 | 0.587 | 0.246 | 0.001 | 0.481 | 0.660 | 0.514 | 0.675 | 0.167 | 0.223 | 0.421 | 0.079 |
| S5 | S10 | 0.819 | 0.117 | 0.747 | 0.519 | 0.190 | 0.001 | 0.301 | 0.592 | 0.520 | 0.675 | 0.164 | 0.223 | 0.414 | 0.079 |
| S5 | S11 | 0.369 | 0.599 | - | - | - | - | - | - | - | - | - | - | - | - |
| S5 | S12 | 0.959 | 0.361 | - | - | - | - | 0.125 | 0.368 | - | - | - | - | - | - |
| S5 | S13 | 0.123 | 0.645 | - | - | - | - | - | - | - | - | - | - | - | - |
| S5 | S14 | - | - | 0.970 | 0.322 | 0.672 | 0.001 | 0.900 | 0.724 | 0.246 | 0.731 | - | - | - | - |
| S5 | S15 | - | - | 0.479 | 0.033 | - | - | 0.421 | 0.519 | 0.517 | 0.662 | 0.210 | 0.272 | 0.884 | 0.167 |
| S5 | S16 | - | - | 0.973 | 0.322 | 0.656 | 0.001 | 0.900 | 0.724 | 0.246 | 0.731 | - | - | - | - |
| S5 | S17 | - | - | 0.974 | 0.322 | 0.658 | 0.001 | 0.902 | 0.724 | 0.241 | 0.731 | - | - | - | - |
| S5 | S18 | 0.269 | 0.462 | - | - | - | - | - | - | - | - | - | - | - | - |
| S5 | S20 | 0.522 | 0.023 | 0.137 | 0.471 | 0.171 | 0.001 | 0.731 | 0.713 | 0.681 | 0.722 | 0.396 | 0.151 | 0.145 | 0.036 |
| S5 | S22 | - | - | 0.967 | 0.322 | 0.651 | 0.001 | 0.906 | 0.724 | 0.243 | 0.731 | - | - | - | - |
| S5 | S24 | 0.322 | 0.012 | 0.133 | 0.471 | 0.180 | 0.001 | 0.703 | 0.724 | 0.689 | 0.722 | 0.516 | 0.142 | 0.243 | 0.051 |
| S5 | S25 | - | - | - | - | - | - | - | - | - | - | 0.569 | 0.376 | 0.954 | 0.215 |
| S5 | S27 | 0.529 | 0.056 | 0.061 | 0.043 | 0.667 | <0.001 | 0.474 | 0.658 | 0.654 | 0.719 | 0.333 | 0.163 | 0.795 | 0.269 |
| S5 | S28 | 0.211 | 0.025 | 0.840 | 0.571 | 0.244 | 0.001 | 0.305 | 0.655 | 0.280 | 0.743 | 0.424 | 0.288 | 0.379 | 0.086 |
| S5 | S29 | 0.517 | 0.056 | 0.619 | 0.395 | 0.607 | 0.001 | 0.306 | 0.625 | 0.716 | 0.749 | 0.578 | 0.303 | **0.012** | **0.001** |
| S6 | S7 | 0.687 | 0.071 | 0.274 | 0.041 | 0.298 | 0.047 | 0.482 | 0.704 | 0.834 | 0.473 | 0.185 | 0.313 | - | - |
| S6 | S8 | 0.671 | 0.071 | 0.276 | 0.041 | 0.303 | 0.047 | 0.487 | 0.704 | 0.832 | 0.473 | 0.186 | 0.313 | - | - |
| S6 | S9 | - | - | 0.238 | 0.894 | 0.369 | 0.406 | 0.501 | 0.900 | 0.999 | 0.483 | 0.999 | 0.313 | 1.000 | 0.076 |
| S6 | S10 | 0.994 | 0.066 | 1.000 | 0.878 | 0.304 | 0.172 | 1.000 | 0.768 | 0.999 | 0.483 | 0.999 | 0.313 | 1.000 | 0.076 |
| S6 | S11 | 0.334 | 0.071 | - | - | - | - | - | - | - | - | - | - | - | - |
| S6 | S12 | 0.053 | 0.005 | - | - | - | - | 0.274 | 0.909 | - | - | - | - | - | - |
| S6 | S13 | 0.025 | 0.081 | - | - | - | - | - | - | - | - | - | - | - | - |
| S6 | S14 | - | - | 0.048 | 0.438 | 0.338 | 0.800 | 0.964 | 0.939 | 0.537 | 0.775 | - | - | - | - |
| S6 | S15 | - | - | **0.002** | **0.036** | - | - | 0.377 | 0.565 | 0.445 | 0.606 | 0.758 | 0.558 | 0.854 | 0.205 |
| S6 | S16 | - | - | 0.047 | 0.438 | 0.441 | 0.856 | 0.959 | 0.939 | 0.523 | 0.775 | - | - | - | - |
| S6 | S17 | - | - | 0.047 | 0.438 | 0.435 | 0.856 | 0.962 | 0.939 | 0.537 | 0.775 | - | - | - | - |
| S6 | S18 | 0.015 | 0.065 | - | - | - | - | - | - | - | - | - | - | - | - |
| S6 | S20 | 0.277 | 0.004 | 0.412 | 0.546 | 0.816 | 0.065 | 0.166 | 0.835 | 0.049 | 0.601 | 0.174 | 0.111 | 0.648 | 0.198 |
| S6 | S22 | - | - | 0.050 | 0.438 | 0.433 | 0.856 | 0.962 | 0.939 | 0.526 | 0.775 | - | - | - | - |
| S6 | S24 | 0.293 | 0.007 | 0.409 | 0.546 | 0.804 | 0.065 | 0.192 | 0.892 | 0.047 | 0.601 | **0.042** | **0.037** | 0.691 | 0.214 |
| S6 | S25 | - | - | - | - | - | - | - | - | - | - | 0.195 | 0.396 | 0.796 | 0.206 |
| S6 | S27 | **0.005** | **0.002** | 0.336 | 0.130 | 0.210 | 0.015 | 0.185 | 0.658 | 0.661 | 0.746 | 0.027 | 0.137 | **0.002** | **0.049** |
| S6 | S28 | **0.005** | **0.011** | 0.744 | 0.868 | 0.319 | 0.419 | 0.496 | 0.776 | 0.566 | 0.653 | 0.803 | 0.309 | 0.033 | 0.165 |
| S6 | S29 | **0.005** | **0.002** | 0.186 | 0.230 | 0.163 | 0.011 | 0.158 | 0.606 | 0.640 | 0.723 | 0.018 | 0.083 | 0.001 | 0.053 |
| S7 | S8 | 0.557 | 0.472 | 1.000 | 0.012 | 0.430 | 0.017 | 1.000 | 0.417 | 0.253 | 0.357 | 0.501 | 1.000 | - | - |
| S7 | S9 | - | - | 0.332 | 0.036 | 0.406 | 0.034 | 0.414 | 0.719 | 0.830 | 0.473 | 0.178 | 0.313 | - | - |
| S7 | S10 | 0.603 | 0.119 | 0.273 | 0.041 | 0.415 | 0.050 | 0.480 | 0.704 | 0.832 | 0.473 | 0.182 | 0.313 | - | - |
| S7 | S11 | 0.046 | 0.623 | - | - | - | - | - | - | - | - | - | - | - | - |
| S7 | S12 | 0.959 | 0.297 | - | - | - | - | 0.324 | 0.417 | - | - | - | - | - | - |
| S7 | S13 | 0.480 | 0.734 | - | - | - | - | - | - | - | - | - | - | - | - |
| S7 | S14 | - | - | 0.416 | 0.033 | 0.785 | 0.047 | 0.611 | 0.695 | 0.028 | 0.147 | - | - | - | - |
| S7 | S15 | - | - | 0.389 | 0.008 | - | - | 0.471 | 0.607 | 0.853 | 0.491 | 0.345 | 0.735 | - | - |
| S7 | S16 | - | - | 0.426 | 0.033 | 0.739 | 0.052 | 0.605 | 0.695 | 0.029 | 0.147 | - | - | - | - |
| S7 | S17 | - | - | 0.418 | 0.033 | 0.736 | 0.052 | 0.611 | 0.695 | 0.027 | 0.147 | - | - | - | - |
| S7 | S18 | 0.157 | 1.000 | - | - | - | - | - | - | - | - | - | - | - | - |
| S7 | S20 | **<0.001** | **0.015** | 0.297 | 0.074 | 0.374 | 0.038 | 0.667 | 0.862 | 0.169 | 0.445 | **0.012** | **0.007** | - | - |
| S7 | S22 | - | - | 0.422 | 0.033 | 0.746 | 0.052 | 0.610 | 0.695 | 0.027 | 0.147 | - | - | - | - |
| S7 | S24 | **<0.001** | **0.027** | 0.301 | 0.074 | 0.378 | 0.038 | 0.578 | 0.863 | 0.169 | 0.445 | **0.006** | **0.004** | - | - |
| S7 | S25 | - | - | - | - | - | - | - | - | - | - | 0.205 | 0.371 | - | - |
| S7 | S27 | 0.068 | 0.331 | 0.236 | 0.012 | 0.920 | 0.017 | 0.699 | 0.705 | 0.770 | 0.576 | 0.052 | 0.198 | - | - |
| S7 | S28 | 0.856 | 0.019 | 0.263 | 0.042 | 0.452 | 0.038 | 0.790 | 0.719 | 0.934 | 0.560 | 0.122 | 0.203 | - | - |
| S7 | S29 | 0.064 | 0.331 | 0.358 | 0.025 | 0.897 | 0.013 | 0.846 | 0.683 | 0.655 | 0.700 | 0.033 | 0.122 | - | - |
| S8 | S9 | - | - | 0.323 | 0.036 | 0.405 | 0.034 | 0.411 | 0.719 | 0.825 | 0.473 | 0.184 | 0.313 | - | - |
| S8 | S10 | 0.598 | 0.119 | 0.272 | 0.041 | 0.412 | 0.050 | 0.478 | 0.704 | 0.832 | 0.473 | 0.189 | 0.313 | - | - |
| S8 | S11 | 0.045 | 0.623 | - | - | - | - | - | - | - | - | - | - | - | - |
| S8 | S12 | 0.958 | 0.297 | - | - | - | - | 0.328 | 0.417 | - | - | - | - | - | - |
| S8 | S13 | 0.483 | 0.734 | - | - | - | - | - | - | - | - | - | - | - | - |
| S8 | S14 | - | - | 0.421 | 0.033 | 0.784 | 0.047 | 0.613 | 0.695 | 0.027 | 0.147 | - | - | - | - |
| S8 | S15 | - | - | 0.389 | 0.008 | - | - | 0.468 | 0.607 | 0.852 | 0.491 | 0.344 | 0.735 | - | - |
| S8 | S16 | - | - | 0.428 | 0.033 | 0.741 | 0.052 | 0.608 | 0.695 | 0.027 | 0.147 | - | - | - | - |
| S8 | S17 | - | - | 0.426 | 0.033 | 0.746 | 0.052 | 0.615 | 0.695 | 0.029 | 0.147 | - | - | - | - |
| S8 | S18 | 0.157 | 1.000 | - | - | - | - | - | - | - | - | - | - | - | - |
| S8 | S20 | **0.001** | **0.015** | 0.299 | 0.074 | 0.380 | 0.038 | 0.667 | 0.862 | 0.175 | 0.445 | **0.011** | **0.007** | - | - |
| S8 | S22 | - | - | 0.422 | 0.033 | 0.740 | 0.052 | 0.617 | 0.695 | 0.030 | 0.147 | - | - | - | - |
| S8 | S24 | **<0.001** | **0.027** | 0.295 | 0.074 | 0.377 | 0.038 | 0.569 | 0.863 | 0.183 | 0.445 | **0.006** | **0.004** | - | - |
| S8 | S25 | - | - | - | - | - | - | - | - | - | - | 0.203 | 0.371 | - | - |
| S8 | S27 | 0.064 | 0.331 | 0.240 | 0.012 | 0.917 | 0.017 | 0.700 | 0.705 | 0.767 | 0.576 | 0.051 | 0.198 | - | - |
| S8 | S28 | 0.850 | 0.019 | 0.263 | 0.042 | 0.458 | 0.038 | 0.788 | 0.719 | 0.928 | 0.560 | 0.121 | 0.203 | - | - |
| S8 | S29 | 0.064 | 0.331 | 0.361 | 0.025 | 0.891 | 0.013 | 0.843 | 0.683 | 0.662 | 0.700 | 0.030 | 0.122 | - | - |
| S9 | S10 | - | - | 0.242 | 0.894 | 0.318 | 0.310 | 0.506 | 0.900 | 0.999 | 0.483 | 0.999 | 0.313 | 1.000 | 0.076 |
| S9 | S12 | - | - | - | - | - | - | 0.248 | 0.942 | - | - | - | - | - | - |
| S9 | S14 | - | - | 0.066 | 0.466 | 0.275 | 0.640 | 0.930 | 1.000 | 0.533 | 0.775 | - | - | - | - |
| S9 | S15 | - | - | **0.003** | **0.039** | - | - | 0.404 | 0.617 | 0.448 | 0.606 | 0.754 | 0.558 | 0.847 | 0.205 |
| S9 | S16 | - | - | 0.063 | 0.466 | 0.318 | 0.686 | 0.934 | 1.000 | 0.534 | 0.775 | - | - | - | - |
| S9 | S17 | - | - | 0.060 | 0.466 | 0.313 | 0.686 | 0.929 | 1.000 | 0.528 | 0.775 | - | - | - | - |
| S9 | S20 | - | - | 0.421 | 0.523 | 0.911 | 0.049 | 0.339 | 0.859 | 0.051 | 0.601 | 0.171 | 0.111 | 0.642 | 0.198 |
| S9 | S22 | - | - | 0.065 | 0.466 | 0.306 | 0.686 | 0.932 | 1.000 | 0.527 | 0.775 | - | - | - | - |
| S9 | S24 | - | - | 0.418 | 0.523 | 0.911 | 0.049 | 0.389 | 0.920 | 0.049 | 0.601 | **0.043** | **0.037** | 0.692 | 0.214 |
| S9 | S25 | - | - | - | - | - | - | - | - | - | - | 0.204 | 0.396 | 0.802 | 0.206 |
| S9 | S27 | - | - | 0.418 | 0.168 | 0.426 | 0.019 | 0.335 | 0.731 | 0.666 | 0.746 | 0.026 | 0.137 | **0.002** | **0.049** |
| S9 | S28 | - | - | 0.104 | 0.213 | 0.879 | 0.488 | 1.000 | 0.909 | 0.568 | 0.653 | 0.805 | 0.309 | 0.030 | 0.165 |
| S9 | S29 | - | - | 0.241 | 0.290 | 0.364 | 0.014 | 0.290 | 0.678 | 0.651 | 0.723 | 0.018 | 0.083 | 0.002 | 0.053 |
| S10 | S11 | 0.323 | 0.120 | - | - | - | - | - | - | - | - | - | - | - | - |
| S10 | S12 | **0.046** | **0.007** | - | - | - | - | 0.271 | 0.909 | - | - | - | - | - | - |
| S10 | S13 | 0.040 | 0.137 | - | - | - | - | - | - | - | - | - | - | - | - |
| S10 | S14 | - | - | 0.052 | 0.438 | 0.299 | 0.422 | 0.960 | 0.939 | 0.534 | 0.775 | - | - | - | - |
| S10 | S15 | - | - | 0.003 | 0.036 | - | - | 0.370 | 0.565 | 0.439 | 0.606 | 0.760 | 0.558 | 0.849 | 0.205 |
| S10 | S16 | - | - | 0.050 | 0.438 | 0.332 | 0.455 | 0.960 | 0.939 | 0.534 | 0.775 | - | - | - | - |
| S10 | S17 | - | - | 0.049 | 0.438 | 0.329 | 0.455 | 0.961 | 0.939 | 0.536 | 0.775 | - | - | - | - |
| S10 | S18 | 0.026 | 0.111 | - | - | - | - | - | - | - | - | - | - | - | - |
| S10 | S20 | 0.239 | 0.006 | 0.406 | 0.546 | 0.972 | 0.030 | 0.163 | 0.835 | 0.045 | 0.601 | 0.162 | 0.111 | 0.643 | 0.198 |
| S10 | S22 | - | - | 0.050 | 0.438 | 0.331 | 0.455 | 0.957 | 0.939 | 0.521 | 0.775 | - | - | - | - |
| S10 | S24 | 0.246 | 0.011 | 0.412 | 0.546 | 0.975 | 0.030 | 0.184 | 0.892 | 0.048 | 0.601 | **0.044** | **0.037** | 0.695 | 0.214 |
| S10 | S25 | - | - | - | - | - | - | - | - | - | - | 0.196 | 0.396 | 0.803 | 0.206 |
| S10 | S27 | **0.006** | **0.006** | 0.336 | 0.130 | 0.357 | 0.017 | 0.179 | 0.658 | 0.666 | 0.746 | 0.028 | 0.137 | **0.003** | **0.049** |
| S10 | S28 | 0.276 | 0.027 | 0.747 | 0.868 | 0.325 | 0.272 | 0.501 | 0.776 | 0.568 | 0.653 | 0.803 | 0.309 | 0.032 | 0.165 |
| S10 | S29 | **0.007** | **0.006** | 0.184 | 0.230 | 0.316 | 0.013 | 0.157 | 0.606 | 0.650 | 0.723 | 0.018 | 0.083 | 0.002 | 0.053 |
| S11 | S12 | 0.047 | 0.240 | - | - | - | - | - | - | - | - | - | - | - | - |
| S11 | S13 | 0.192 | 0.825 | - | - | - | - | - | - | - | - | - | - | - | - |
| S11 | S18 | 0.585 | 0.711 | - | - | - | - | - | - | - | - | - | - | - | - |
| S11 | S20 | 0.763 | 0.019 | - | - | - | - | - | - | - | - | - | - | - | - |
| S11 | S24 | 0.764 | 0.033 | - | - | - | - | - | - | - | - | - | - | - | - |
| S11 | S27 | 0.145 | 0.213 | - | - | - | - | - | - | - | - | - | - | - | - |
| S11 | S28 | 0.378 | 0.019 | - | - | - | - | - | - | - | - | - | - | - | - |
| S11 | S29 | 0.147 | 0.213 | - | - | - | - | - | - | - | - | - | - | - | - |
| S12 | S13 | 0.068 | 0.302 | - | - | - | - | - | - | - | - | - | - | - | - |
| S12 | S14 | - | - | - | - | - | - | 0.500 | 0.834 | - | - | - | - | - | - |
| S12 | S15 | - | - | - | - | - | - | 0.303 | 0.557 | - | - | - | - | - | - |
| S12 | S16 | - | - | - | - | - | - | 0.507 | 0.834 | - | - | - | - | - | - |
| S12 | S17 | - | - | - | - | - | - | 0.496 | 0.834 | - | - | - | - | - | - |
| S12 | S18 | 0.412 | 0.268 | - | - | - | - | - | - | - | - | - | - | - | - |
| S12 | S20 | 0.308 | 0.023 | - | - | - | - | 0.388 | 0.585 | - | - | - | - | - | - |
| S12 | S22 | - | - | - | - | - | - | 0.497 | 0.834 | - | - | - | - | - | - |
| S12 | S24 | 0.253 | 0.035 | - | - | - | - | 0.467 | 0.689 | - | - | - | - | - | - |
| S12 | S27 | 0.707 | 0.208 | - | - | - | - | 0.119 | 0.439 | - | - | - | - | - | - |
| S12 | S28 | 0.108 | 0.002 | - | - | - | - | 0.246 | 0.949 | - | - | - | - | - | - |
| S12 | S29 | 0.705 | 0.208 | - | - | - | - | 0.110 | 0.390 | - | - | - | - | - | - |
| S13 | S18 | 0.386 | 0.854 | - | - | - | - | - | - | - | - | - | - | - | - |
| S13 | S20 | **0.002** | **0.026** | - | - | - | - | - | - | - | - | - | - | - | - |
| S13 | S24 | **0.001** | **0.045** | - | - | - | - | - | - | - | - | - | - | - | - |
| S13 | S27 | 0.125 | 0.325 | - | - | - | - | - | - | - | - | - | - | - | - |
| S13 | S28 | **0.005** | **0.022** | - | - | - | - | - | - | - | - | - | - | - | - |
| S13 | S29 | 0.127 | 0.325 | - | - | - | - | - | - | - | - | - | - | - | - |
| S14 | S15 | - | - | 0.118 | 0.023 | - | - | 0.835 | 0.826 | 0.594 | 0.784 | - | 0.736 | - | - |
| S14 | S16 | - | - | 0.996 | 0.371 | 0.779 | 0.749 | 1.000 | 0.834 | 0.631 | 0.880 | - | - | - | - |
| S14 | S17 | - | - | 0.997 | 0.371 | 0.781 | 0.749 | 1.000 | 0.834 | 0.634 | 0.880 | - | - | - | - |
| S14 | S20 | - | - | 0.870 | 0.391 | 0.075 | 0.065 | 0.700 | 0.836 | 0.485 | 0.801 | - | 0.056 | - | - |
| S14 | S22 | - | - | 0.998 | 0.371 | 0.778 | 0.749 | 1.000 | 0.834 | 0.629 | 0.880 | - | - | - | - |
| S14 | S24 | - | - | 0.867 | 0.391 | 0.079 | 0.065 | 0.748 | 0.898 | 0.478 | 0.801 | - | 0.036 | - | - |
| S14 | S25 | - | - | - | - | - | - | - | - | - | - | - | 0.372 | - | - |
| S14 | S27 | - | - | 0.959 | 0.155 | 0.347 | 0.016 | 0.453 | 0.722 | 0.278 | 0.836 | - | 0.205 | - | - |
| S14 | S28 | - | - | 0.042 | 0.412 | 0.336 | 0.696 | 0.923 | 0.973 | 0.625 | 0.896 | - | 0.203 | - | - |
| S14 | S29 | - | - | 0.024 | 0.070 | 0.460 | 0.014 | 0.788 | 0.748 | 0.279 | 0.796 | - | 0.128 | - | - |
| S15 | S16 | - | - | 0.116 | 0.023 | - | - | 0.831 | 0.826 | 0.602 | 0.784 | - | - | - | - |
| S15 | S17 | - | - | 0.118 | 0.023 | - | - | 0.832 | 0.826 | 0.607 | 0.784 | - | - | - | - |
| S15 | S20 | - | - | 0.880 | 0.065 | - | - | 0.086 | 0.224 | 0.699 | 0.618 | 0.177 | 0.109 | 0.382 | 0.898 |
| S15 | S22 | - | - | 0.119 | 0.023 | - | - | 0.824 | 0.826 | 0.603 | 0.784 | - | - | - | - |
| S15 | S24 | - | - | 0.872 | 0.065 | - | - | 0.070 | 0.223 | 0.689 | 0.618 | 0.503 | 0.111 | 0.940 | 0.968 |
| S15 | S25 | - | - | - | - | - | - | - | - | - | - | 0.331 | 0.627 | 0.531 | 0.990 |
| S15 | S27 | - | - | 0.129 | 0.015 | - | - | 0.262 | 0.382 | 0.941 | 0.648 | 0.402 | 0.441 | 0.296 | 0.901 |
| S15 | S28 | - | - | **0.002** | **0.033** | - | - | 0.403 | 0.164 | 0.558 | 0.706 | 0.829 | 0.537 | 0.019 | 0.371 |
| S15 | S29 | - | - | 0.957 | 0.114 | - | - | 0.265 | 0.387 | 0.910 | 0.613 | 0.348 | 0.266 | 0.658 | 0.999 |
| S16 | S17 | - | - | 0.997 | 0.371 | 0.376 | 0.752 | 1.000 | 0.834 | 0.627 | 0.880 | - | - | - | - |
| S16 | S20 | - | - | 0.870 | 0.391 | 0.118 | 0.071 | 0.710 | 0.836 | 0.488 | 0.801 | - | - | - | - |
| S16 | S22 | - | - | 0.998 | 0.371 | 0.380 | 0.752 | 1.000 | 0.834 | 0.622 | 0.880 | - | - | - | - |
| S16 | S24 | - | - | 0.865 | 0.391 | 0.111 | 0.071 | 0.748 | 0.898 | 0.477 | 0.801 | - | - | - | - |
| S16 | S27 | - | - | 0.957 | 0.155 | 0.334 | 0.015 | 0.452 | 0.722 | 0.275 | 0.836 | - | - | - | - |
| S16 | S28 | - | - | 0.042 | 0.412 | 0.327 | 0.746 | 0.925 | 0.973 | 0.621 | 0.896 | - | - | - | - |
| S16 | S29 | - | - | 0.025 | 0.070 | 0.411 | 0.013 | 0.788 | 0.748 | 0.280 | 0.796 | - | - | - | - |
| S17 | S20 | - | - | 0.870 | 0.391 | 0.116 | 0.071 | 0.701 | 0.836 | 0.486 | 0.801 | - | - | - | - |
| S17 | S22 | - | - | 0.997 | 0.371 | 0.378 | 0.752 | 1.000 | 0.834 | 0.619 | 0.880 | - | - | - | - |
| S17 | S24 | - | - | 0.865 | 0.391 | 0.116 | 0.071 | 0.747 | 0.898 | 0.484 | 0.801 | - | - | - | - |
| S17 | S27 | - | - | 0.956 | 0.155 | 0.327 | 0.015 | 0.452 | 0.722 | 0.282 | 0.836 | - | - | - | - |
| S17 | S28 | - | - | 0.043 | 0.412 | 0.318 | 0.746 | 0.924 | 0.973 | 0.626 | 0.896 | - | - | - | - |
| S17 | S29 | - | - | 0.024 | 0.070 | 0.412 | 0.013 | 0.785 | 0.748 | 0.274 | 0.796 | - | - | - | - |
| S18 | S20 | 0.183 | 0.024 | - | - | - | - | - | - | - | - | - | - | - | - |
| S18 | S24 | 0.151 | 0.042 | - | - | - | - | - | - | - | - | - | - | - | - |
| S18 | S27 | 0.254 | 0.406 | - | - | - | - | - | - | - | - | - | - | - | - |
| S18 | S28 | **0.004** | **0.017** | - | - | - | - | - | - | - | - | - | - | - | - |
| S18 | S29 | 0.255 | 0.406 | - | - | - | - | - | - | - | - | - | - | - | - |
| S20 | S22 | - | - | 0.867 | 0.391 | 0.112 | 0.071 | 0.700 | 0.836 | 0.474 | 0.801 | - | - | - | - |
| S20 | S24 | 0.645 | 0.020 | 0.343 | 0.272 | 1.000 | 0.024 | 0.281 | 0.595 | 0.441 | 0.512 | 0.033 | 0.051 | 0.741 | 0.682 |
| S20 | S25 | - | - | - | - | - | - | - | - | - | - | 0.695 | 0.118 | 0.111 | 0.375 |
| S20 | S27 | 0.759 | 0.046 | 0.504 | 0.342 | 0.745 | 0.013 | 0.310 | 0.777 | 0.834 | 0.818 | 0.043 | 0.097 | 0.909 | 0.926 |
| S20 | S28 | 0.516 | 0.001 | 0.414 | 0.539 | 0.877 | 0.054 | 0.135 | 0.859 | 0.084 | 0.706 | 0.229 | 0.086 | 0.151 | 0.491 |
| S20 | S29 | 0.770 | 0.046 | 0.369 | 0.448 | 0.527 | 0.010 | 0.353 | 0.738 | 0.698 | 0.771 | 0.216 | 0.248 | 0.937 | 0.790 |
| S22 | S24 | - | - | 0.868 | 0.391 | 0.114 | 0.071 | 0.748 | 0.898 | 0.478 | 0.801 | - | - | - | - |
| S22 | S27 | - | - | 0.960 | 0.155 | 0.333 | 0.015 | 0.439 | 0.722 | 0.278 | 0.836 | - | - | - | - |
| S22 | S28 | - | - | 0.042 | 0.412 | 0.323 | 0.746 | 0.928 | 0.973 | 0.627 | 0.896 | - | - | - | - |
| S22 | S29 | - | - | 0.023 | 0.070 | 0.421 | 0.013 | 0.798 | 0.748 | 0.277 | 0.796 | - | - | - | - |
| S24 | S25 | - | - | - | - | - | - | - | - | - | - | 0.678 | 0.082 | 0.103 | 0.384 |
| S24 | S27 | 0.580 | 0.045 | 0.503 | 0.342 | 0.737 | 0.013 | 0.282 | 0.768 | 0.833 | 0.818 | 0.225 | 0.152 | 0.956 | 0.914 |
| S24 | S28 | 0.530 | 0.002 | 0.412 | 0.539 | 0.880 | 0.054 | 0.162 | 0.920 | 0.081 | 0.706 | 0.061 | 0.060 | 0.171 | 1.000 |
| S24 | S29 | 0.570 | 0.045 | 0.370 | 0.448 | 0.533 | 0.010 | 0.334 | 0.748 | 0.701 | 0.771 | 0.819 | 0.098 | 0.783 | 0.842 |
| S25 | S27 | - | - | - | - | - | - | - | - | - | - | 0.667 | 0.320 | 0.186 | 0.984 |
| S25 | S28 | - | - | - | - | - | - | - | - | - | - | 0.141 | 0.291 | 0.510 | 0.371 |
| S25 | S29 | - | - | - | - | - | - | - | - | - | - | 0.689 | 0.227 | 0.964 | 0.990 |
| S27 | S28 | **0.003** | **<0.001** | 0.287 | 0.109 | 0.425 | 0.018 | 0.201 | 0.726 | 0.379 | 0.834 | 0.018 | 0.081 | 0.032 | 0.279 |
| S27 | S29 | 0.394 | 0.139 | 0.761 | 0.103 | 0.448 | 0.010 | 0.719 | 0.482 | 0.718 | 0.566 | 0.188 | 0.077 | 0.622 | 0.985 |
| S28 | S29 | **0.003** | **<0.001** | 0.229 | 0.497 | 0.359 | 0.013 | 0.166 | 0.673 | 0.361 | 0.802 | 0.091 | 0.095 | 0.003 | 0.079 |

1. Comparison between control and schizophrenia groups

| SNP X | SNP Y | GE | | | | US | | | | JP | | | |
| --- | --- | --- | --- | --- | --- | --- | --- | --- | --- | --- | --- | --- | --- |
|  |  | F | | M | | F | | M | | F | | M | |
|  |  | LD[[4]](#footnote-5) | HF[[5]](#footnote-6) | LD | HF | LD | HF | LD | HF | LD | HF | LD | HF |
| S1 | S2 | - | - | - | - | - | - | - | - | 0.664 | 0.673 | 0.973 | 0.053 |
| S1 | S3 | 0.538 | 0.001 | 0.253 | 0.480 | 0.944 | 0.949 | 0.506 | 0.353 | 0.954 | 0.378 | 0.386 | 0.579 |
| S1 | S4 | - | - | - | - | - | - | - | - | 0.666 | 0.673 | 0.970 | 0.053 |
| S1 | S5 | 0.552 | 0.001 | 0.263 | 0.480 | 0.882 | 0.993 | 0.600 | 0.356 | 0.539 | 0.912 | **0.013[[6]](#footnote-7)** | **0.001** |
| S1 | S6 | 0.958 | 0.333 | 0.554 | 0.352 | 0.706 | 0.656 | 0.551 | 0.351 | 0.748 | 0.805 | 0.371 | 0.307 |
| S1 | S7 | 0.459 | 0.020 | 0.263 | 0.552 | 0.253 | 0.992 | 0.778 | 0.384 | 0.363 | 0.466 | - | 0.273 |
| S1 | S8 | 0.465 | 0.020 | 0.274 | 0.552 | 0.256 | 0.992 | 0.778 | 0.384 | 0.359 | 0.466 | - | 0.273 |
| S1 | S9 | 0.959 | 0.333 | 0.446 | 0.586 | 0.333 | 0.689 | 0.551 | 0.351 | 0.744 | 0.805 | 0.369 | 0.307 |
| S1 | S10 | 0.958 | 0.333 | 0.432 | 0.522 | 0.705 | 0.656 | 0.550 | 0.351 | 0.746 | 0.805 | 0.370 | 0.307 |
| S1 | S14 | 0.280 | 0.717 | 0.318 | 0.343 | 0.543 | 0.932 | 0.009 | 0.115 | - | - | - | - |
| S1 | S15 | - | - | 0.280 | 0.448 | 0.798 | 0.907 | 0.083 | 0.018 | 0.529 | 0.761 | 0.272 | 0.535 |
| S1 | S16 | 0.274 | 0.717 | 0.216 | 0.249 | 0.545 | 0.932 | 0.008 | 0.115 | - | - | - | - |
| S1 | S17 | 0.280 | 0.717 | 0.213 | 0.249 | 0.547 | 0.932 | 0.009 | 0.115 | - | - | - | - |
| S1 | S20 | 0.359 | 0.103 | 0.434 | 0.539 | 0.646 | 0.993 | 0.437 | 0.221 | 0.685 | 0.790 | 0.502 | 0.559 |
| S1 | S21 | - | - | - | - | - | - | - | 0.151 | - | - | - | - |
| S1 | S22 | 0.274 | 0.717 | 0.217 | 0.249 | 0.539 | 0.932 | 0.009 | 0.115 | - | - | - | - |
| S1 | S24 | 0.364 | 0.086 | 0.438 | 0.543 | 0.571 | 0.985 | 0.430 | 0.221 | 0.937 | 0.776 | 0.895 | 0.324 |
| S1 | S25 | - | - | - | - | - | - | - | - | 0.145 | 0.175 | 0.424 | 0.569 |
| S1 | S26 | - | - | - | - | - | - | - | 0.149 | - | - | - | - |
| S1 | S27 | 0.599 | 0.007 | 0.208 | 0.538 | 0.839 | 0.989 | 0.818 | 0.412 | **0.009** | **0.014** | **0.013** | **0.012** |
| S1 | S28 | 0.959 | 0.333 | 0.493 | 0.390 | 0.828 | 0.911 | 0.551 | 0.312 | 0.985 | 0.740 | 0.874 | 0.692 |
| S1 | S29 | 0.779 | 0.007 | 0.287 | 0.526 | 0.853 | 0.973 | 0.955 | 0.413 | 0.651 | 0.809 | 0.205 | 0.187 |
| S2 | S3 | - | - | - | - | - | - | - | - | 0.689 | 0.893 | 0.314 | 0.037 |
| S2 | S4 | - | - | - | - | - | - | - | - | 1.000 | 0.635 | 1.000 | 0.042 |
| S2 | S5 | - | - | - | - | - | - | - | - | 0.723 | 0.775 | 0.970 | 0.034 |
| S2 | S6 | - | - | - | - | - | - | - | - | 0.917 | 0.634 | 0.927 | 0.076 |
| S2 | S7 | - | - | - | - | - | - | - | - | 0.404 | 0.177 | - | 0.044 |
| S2 | S8 | - | - | - | - | - | - | - | - | 0.399 | 0.177 | - | 0.044 |
| S2 | S9 | - | - | - | - | - | - | - | - | 0.920 | 0.634 | 0.920 | 0.076 |
| S2 | S10 | - | - | - | - | - | - | - | - | 0.916 | 0.634 | 0.924 | 0.076 |
| S2 | S15 | - | - | - | - | - | - | - | - | 0.615 | 0.863 | 0.162 | 0.087 |
| S2 | S20 | - | - | - | - | - | - | - | - | 0.359 | 0.768 | 0.674 | 0.138 |
| S2 | S24 | - | - | - | - | - | - | - | - | 0.541 | 0.637 | 0.486 | 0.078 |
| S2 | S25 | - | - | - | - | - | - | - | - | 0.773 | 0.778 | 0.339 | 0.114 |
| S2 | S27 | - | - | - | - | - | - | - | - | 0.827 | 0.583 | 0.191 | 0.092 |
| S2 | S28 | - | - | - | - | - | - | - | - | 0.936 | 0.804 | 0.823 | 0.098 |
| S2 | S29 | - | - | - | - | - | - | - | - | 0.659 | 0.571 | 0.857 | 0.056 |
| S3 | S4 | - | - | - | - | - | - | - | - | 0.698 | 0.893 | 0.315 | 0.037 |
| S3 | S5 | 0.543 | <0.001 | 0.101 | 0.417 | 0.139 | 0.469 | 0.540 | 0.652 | 0.154 | 0.057 | **0.003** | **<0.001** |
| S3 | S6 | 0.080 | 0.001 | 0.814 | 0.399 | 0.799 | 0.790 | 0.846 | 0.941 | 0.925 | 0.992 | 0.715 | 0.539 |
| S3 | S7 | 0.922 | 0.001 | 0.465 | 0.250 | 0.926 | 0.968 | 0.573 | 0.846 | 0.328 | 0.806 | - | 0.270 |
| S3 | S8 | 0.926 | 0.001 | 0.458 | 0.250 | 0.929 | 0.968 | 0.573 | 0.846 | 0.325 | 0.806 | - | 0.270 |
| S3 | S9 | 0.074 | 0.001 | 0.318 | 0.701 | 0.982 | 0.854 | 0.846 | 0.941 | 0.924 | 0.992 | 0.716 | 0.539 |
| S3 | S10 | 0.069 | 0.001 | 0.669 | 0.744 | 0.794 | 0.790 | 0.847 | 0.941 | 0.929 | 0.992 | 0.721 | 0.539 |
| S3 | S13 | - | - | - | - | - | - | 0.287 | - | - | - | - | - |
| S3 | S14 | 0.401 | 0.001 | 0.549 | 0.540 | 0.316 | 0.919 | 0.710 | 0.345 | - | - | - | - |
| S3 | S15 | - | - | 0.133 | 0.680 | 0.303 | 0.901 | 0.182 | 0.916 | 0.933 | 0.908 | 0.501 | 0.558 |
| S3 | S16 | 0.410 | 0.001 | 0.550 | 0.412 | 0.316 | 0.919 | 0.712 | 0.345 | - | - | - | - |
| S3 | S17 | 0.414 | 0.001 | 0.553 | 0.412 | 0.323 | 0.919 | 0.715 | 0.345 | - | - | - | - |
| S3 | S20 | 0.246 | 0.001 | 0.111 | 0.694 | 0.920 | 0.954 | 0.524 | 0.937 | 0.592 | 0.289 | 0.498 | 0.619 |
| S3 | S22 | 0.409 | 0.001 | 0.551 | 0.412 | 0.319 | 0.919 | 0.708 | 0.345 | - | - | - | - |
| S3 | S24 | 0.239 | 0.002 | 0.114 | 0.677 | 0.933 | 0.962 | 0.516 | 0.937 | 0.609 | 0.288 | 0.293 | 0.559 |
| S3 | S25 | - | - | - | - | - | - | - | - | 0.198 | 0.915 | 0.159 | 0.477 |
| S3 | S27 | 0.836 | <0.001 | 0.472 | 0.528 | 0.561 | 0.825 | 0.368 | 0.835 | **0.001** | **<0.001** | 0.051 | 0.284 |
| S3 | S28 | 0.079 | 0.001 | 0.389 | 0.657 | 0.628 | 0.956 | 0.857 | 0.923 | 0.513 | 0.985 | 0.745 | 0.577 |
| S3 | S29 | 0.484 | 0.001 | 0.314 | 0.410 | 0.679 | 0.825 | 0.752 | 0.861 | 0.335 | 0.431 | 0.369 | 0.605 |
| S4 | S5 | - | - | - | - | - | - | - | - | 0.719 | 0.775 | 0.964 | 0.034 |
| S4 | S6 | - | - | - | - | - | - | - | - | 0.918 | 0.634 | 0.924 | 0.076 |
| S4 | S7 | - | - | - | - | - | - | - | - | 0.407 | 0.177 | - | 0.044 |
| S4 | S8 | - | - | - | - | - | - | - | - | 0.404 | 0.177 | - | 0.044 |
| S4 | S9 | - | - | - | - | - | - | - | - | 0.915 | 0.634 | 0.923 | 0.076 |
| S4 | S10 | - | - | - | - | - | - | - | - | 0.913 | 0.634 | 0.922 | 0.076 |
| S4 | S15 | - | - | - | - | - | - | - | - | 0.610 | 0.863 | 0.169 | 0.087 |
| S4 | S20 | - | - | - | - | - | - | - | - | 0.368 | 0.768 | 0.666 | 0.138 |
| S4 | S24 | - | - | - | - | - | - | - | - | 0.550 | 0.637 | 0.487 | 0.078 |
| S4 | S25 | - | - | - | - | - | - | - | - | 0.770 | 0.778 | 0.343 | 0.114 |
| S4 | S27 | - | - | - | - | - | - | - | - | 0.825 | 0.583 | 0.201 | 0.092 |
| S4 | S28 | - | - | - | - | - | - | - | - | 0.942 | 0.804 | 0.834 | 0.098 |
| S4 | S29 | - | - | - | - | - | - | - | - | 0.657 | 0.571 | 0.846 | 0.056 |
| S5 | S6 | 0.079 | 0.001 | 0.822 | 0.399 | 0.760 | 0.770 | 0.722 | 0.880 | 0.805 | 0.823 | 0.494 | 0.051 |
| S5 | S7 | 0.880 | 0.001 | 0.457 | 0.250 | 0.835 | 0.996 | 0.425 | 0.752 | 0.285 | 0.650 | - | 0.012 |
| S5 | S8 | 0.879 | 0.001 | 0.466 | 0.250 | 0.830 | 0.996 | 0.418 | 0.752 | 0.283 | 0.650 | - | 0.012 |
| S5 | S9 | 0.084 | 0.001 | 0.329 | 0.701 | 0.935 | 0.843 | 0.726 | 0.880 | 0.808 | 0.823 | 0.491 | 0.051 |
| S5 | S10 | 0.081 | 0.001 | 0.663 | 0.744 | 0.752 | 0.770 | 0.723 | 0.880 | 0.808 | 0.823 | 0.490 | 0.051 |
| S5 | S13 | - | - | - | - | - | - | 0.211 | - | - | - | - | - |
| S5 | S14 | 0.425 | 0.001 | 0.549 | 0.540 | 0.335 | 0.935 | 0.780 | 0.336 | - | - | - | - |
| S5 | S15 | - | - | 0.205 | 0.662 | 0.285 | 0.907 | 0.213 | 0.851 | 0.779 | 0.744 | 0.060 | 0.031 |
| S5 | S16 | 0.418 | 0.001 | 0.541 | 0.412 | 0.335 | 0.935 | 0.782 | 0.336 | - | - | - | - |
| S5 | S17 | 0.424 | 0.001 | 0.545 | 0.412 | 0.328 | 0.935 | 0.781 | 0.336 | - | - | - | - |
| S5 | S20 | 0.229 | 0.001 | 0.112 | 0.694 | 0.996 | 0.998 | 0.393 | 0.867 | 0.238 | 0.678 | **0.035** | **0.012** |
| S5 | S22 | 0.422 | 0.001 | 0.550 | 0.412 | 0.329 | 0.935 | 0.780 | 0.336 | - | - | - | - |
| S5 | S24 | 0.233 | 0.002 | 0.114 | 0.677 | 0.974 | 0.999 | 0.382 | 0.867 | 0.243 | 0.666 | 0.051 | 0.001 |
| S5 | S25 | - | - | - | - | - | - | - | - | 0.227 | 0.790 | 0.111 | 0.043 |
| S5 | S27 | 0.983 | 0.001 | 0.468 | 0.528 | 0.940 | 0.999 | 1.000 | 0.876 | **0.009** | **0.008** | **0.013** | **0.008** |
| S5 | S28 | 0.083 | 0.001 | 0.386 | 0.657 | 0.666 | 0.968 | 0.979 | 0.855 | 0.793 | 0.878 | 0.603 | 0.055 |
| S5 | S29 | 0.583 | 0.002 | 0.316 | 0.410 | 0.933 | 0.999 | 0.718 | 0.849 | 0.515 | 0.712 | **0.007** | **0.001** |
| S6 | S7 | **0.001** | **0.013** | 0.039 | 0.529 | 0.474 | 0.780 | 0.228 | 0.986 | 0.388 | 0.799 | - | 0.421 |
| S6 | S8 | **0.001** | **0.013** | 0.040 | 0.529 | 0.475 | 0.780 | 0.234 | 0.986 | 0.392 | 0.799 | - | 0.421 |
| S6 | S9 | 0.994 | 0.243 | 0.062 | 0.027 | 0.860 | 0.571 | 0.595 | 0.938 | 0.901 | 0.905 | 0.488 | 0.414 |
| S6 | S10 | 0.995 | 0.243 | **0.005** | **0.001** | 0.332 | 0.493 | 0.590 | 0.938 | 0.906 | 0.905 | 0.490 | 0.414 |
| S6 | S13 | - | - | - | - | - | - | 0.196 | - | - | - | - | - |
| S6 | S14 | 0.734 | 0.469 | 0.156 | 0.382 | 0.819 | 0.733 | 0.838 | 0.343 | - | - | - | - |
| S6 | S15 | - | - | 0.018 | 0.498 | 0.809 | 0.710 | 0.095 | 0.988 | 0.972 | 0.906 | 0.336 | 0.706 |
| S6 | S16 | 0.735 | 0.469 | 0.102 | 0.278 | 0.823 | 0.733 | 0.842 | 0.343 | - | - | - | - |
| S6 | S17 | 0.733 | 0.469 | 0.094 | 0.278 | 0.819 | 0.733 | 0.841 | 0.343 | - | - | - | - |
| S6 | S20 | 0.312 | 0.057 | 0.237 | 0.466 | 0.074 | 0.790 | 0.383 | 0.992 | 0.721 | 0.668 | 0.427 | 0.574 |
| S6 | S22 | 0.727 | 0.469 | 0.098 | 0.278 | 0.825 | 0.733 | 0.829 | 0.343 | - | - | - | - |
| S6 | S24 | 0.308 | 0.049 | 0.247 | 0.433 | 0.063 | 0.787 | 0.389 | 0.992 | 0.712 | 0.655 | 0.078 | 0.202 |
| S6 | S25 | - | - | - | - | - | - | - | - | 0.614 | 0.855 | 0.574 | 0.625 |
| S6 | S27 | 0.154 | 0.008 | 0.348 | 0.439 | 0.812 | 0.771 | 0.729 | 0.881 | 0.215 | 0.304 | 0.881 | 0.808 |
| S6 | S28 | 0.993 | 0.243 | 0.004 | 0.096 | 0.082 | 0.110 | 0.790 | 0.945 | 0.054 | 0.047 | 0.254 | 0.748 |
| S6 | S29 | 0.074 | 0.008 | 0.426 | 0.430 | 0.842 | 0.771 | 0.684 | 0.896 | 0.592 | 0.721 | 0.986 | 0.624 |
| S7 | S8 | 1.000 | 0.010 | 0.234 | 0.541 | 1.000 | 0.927 | 0.331 | 0.874 | 0.365 | 0.511 | - | 1.000 |
| S7 | S9 | **0.001** | **0.013** | 0.027 | 0.830 | 0.519 | 0.846 | 0.228 | 0.986 | 0.387 | 0.799 | - | 0.421 |
| S7 | S10 | **0.002** | **0.013** | 0.034 | 0.111 | 0.483 | 0.780 | 0.235 | 0.986 | 0.390 | 0.799 | - | 0.421 |
| S7 | S13 | - | - | - | - | - | - | 0.294 | - | - | - | - | - |
| S7 | S14 | 0.158 | 0.033 | 0.695 | 0.575 | 0.018 | 0.930 | 0.896 | 0.345 | - | - | - | - |
| S7 | S15 | - | - | 0.268 | 0.748 | 0.541 | 0.980 | 0.725 | 0.975 | 0.388 | 0.731 | - | 0.922 |
| S7 | S16 | 0.156 | 0.033 | 0.961 | 0.429 | 0.019 | 0.930 | 0.891 | 0.345 | - | - | - | - |
| S7 | S17 | 0.158 | 0.033 | 0.959 | 0.429 | 0.018 | 0.930 | 0.889 | 0.345 | - | - | - | - |
| S7 | S20 | 0.482 | 0.037 | 0.226 | 0.764 | 0.489 | 0.982 | 0.297 | 0.927 | 0.041 | 0.118 | - | 0.460 |
| S7 | S22 | 0.163 | 0.033 | 0.962 | 0.429 | 0.019 | 0.930 | 0.893 | 0.345 | - | - | - | - |
| S7 | S24 | 0.498 | 0.033 | 0.221 | 0.713 | 0.437 | 0.973 | 0.304 | 0.927 | 0.036 | 0.115 | - | 0.213 |
| S7 | S25 | - | - | - | - | - | - | - | - | 0.223 | 0.692 | - | 0.580 |
| S7 | S27 | 0.634 | 0.007 | 0.019 | 0.186 | 0.860 | 0.997 | 0.839 | 0.756 | 0.114 | 0.571 | - | 0.502 |
| S7 | S28 | **0.001** | **0.013** | 0.012 | 0.662 | 0.683 | 0.963 | 0.492 | 0.984 | 0.267 | 0.721 | - | 0.616 |
| S7 | S29 | 0.681 | 0.007 | 0.027 | 0.199 | 0.893 | 0.997 | 0.805 | 0.859 | 0.115 | 0.515 | - | 0.331 |
| S8 | S9 | **0.002** | **0.013** | 0.031 | 0.830 | 0.518 | 0.846 | 0.233 | 0.986 | 0.389 | 0.799 | - | 0.421 |
| S8 | S10 | **0.001** | **0.013** | 0.036 | 0.111 | 0.471 | 0.780 | 0.233 | 0.986 | 0.391 | 0.799 | - | 0.421 |
| S8 | S13 | - | - | - | - | - | - | 0.295 | - | - | - | - | - |
| S8 | S14 | 0.156 | 0.033 | 0.699 | 0.575 | 0.018 | 0.930 | 0.891 | 0.345 | - | - | - | - |
| S8 | S15 | - | - | 0.262 | 0.748 | 0.547 | 0.980 | 0.730 | 0.975 | 0.398 | 0.731 | - | 0.922 |
| S8 | S16 | 0.156 | 0.033 | 0.963 | 0.429 | 0.020 | 0.930 | 0.893 | 0.345 | - | - | - | - |
| S8 | S17 | 0.159 | 0.033 | 0.965 | 0.429 | 0.018 | 0.930 | 0.892 | 0.345 | - | - | - | - |
| S8 | S20 | 0.482 | 0.037 | 0.222 | 0.764 | 0.496 | 0.982 | 0.298 | 0.927 | 0.038 | 0.118 | - | 0.460 |
| S8 | S22 | 0.154 | 0.033 | 0.961 | 0.429 | 0.019 | 0.930 | 0.893 | 0.345 | - | - | - | - |
| S8 | S24 | 0.478 | 0.033 | 0.224 | 0.713 | 0.427 | 0.973 | 0.303 | 0.927 | 0.035 | 0.115 | - | 0.213 |
| S8 | S25 | - | - | - | - | - | - | - | - | 0.218 | 0.692 | - | 0.580 |
| S8 | S27 | 0.644 | 0.007 | 0.018 | 0.186 | 0.855 | 0.997 | 0.844 | 0.756 | 0.119 | 0.571 | - | 0.502 |
| S8 | S28 | **0.001** | **0.013** | 0.010 | 0.662 | 0.685 | 0.963 | 0.499 | 0.984 | 0.281 | 0.721 | - | 0.616 |
| S8 | S29 | 0.681 | 0.007 | 0.029 | 0.199 | 0.898 | 0.997 | 0.805 | 0.859 | 0.109 | 0.515 | - | 0.331 |
| S9 | S10 | 0.993 | 0.243 | 0.384 | 0.556 | 0.867 | 0.571 | 0.591 | 0.938 | 0.903 | 0.905 | 0.481 | 0.414 |
| S9 | S13 | - | - | - | - | - | - | 0.197 | - | - | - | - | - |
| S9 | S14 | 0.725 | 0.469 | 0.240 | 0.658 | 0.807 | 0.795 | 0.839 | 0.343 | - | - | - | - |
| S9 | S15 | - | - | 0.045 | 0.849 | 0.787 | 0.770 | 0.090 | 0.988 | 0.973 | 0.906 | 0.345 | 0.706 |
| S9 | S16 | 0.735 | 0.469 | 0.160 | 0.484 | 0.810 | 0.795 | 0.833 | 0.343 | - | - | - | - |
| S9 | S17 | 0.740 | 0.469 | 0.174 | 0.484 | 0.813 | 0.795 | 0.836 | 0.343 | - | - | - | - |
| S9 | S20 | 0.313 | 0.057 | 0.501 | 0.823 | 0.026 | 0.855 | 0.394 | 0.992 | 0.724 | 0.668 | 0.435 | 0.574 |
| S9 | S22 | 0.736 | 0.469 | 0.161 | 0.484 | 0.804 | 0.795 | 0.838 | 0.343 | - | - | - | - |
| S9 | S24 | 0.314 | 0.049 | 0.494 | 0.770 | 0.027 | 0.853 | 0.380 | 0.992 | 0.713 | 0.655 | 0.088 | 0.202 |
| S9 | S25 | - | - | - | - | - | - | - | - | 0.627 | 0.855 | 0.557 | 0.625 |
| S9 | S27 | 0.158 | 0.008 | 0.800 | 0.873 | 0.990 | 0.844 | 0.731 | 0.881 | 0.226 | 0.304 | 0.879 | 0.808 |
| S9 | S28 | 0.994 | 0.243 | 0.740 | 0.764 | 0.219 | 0.596 | 0.785 | 0.945 | 0.052 | 0.047 | 0.262 | 0.748 |
| S9 | S29 | 0.075 | 0.008 | 0.668 | 0.816 | 0.973 | 0.844 | 0.677 | 0.896 | 0.598 | 0.721 | 0.988 | 0.624 |
| S10 | S13 | - | - | - | - | - | - | 0.197 | - | - | - | - | - |
| S10 | S14 | 0.734 | 0.469 | 0.208 | 0.632 | 0.815 | 0.733 | 0.837 | 0.343 | - | - | - | - |
| S10 | S15 | - | - | 0.043 | 0.810 | 0.803 | 0.710 | 0.088 | 0.988 | 0.971 | 0.906 | 0.336 | 0.706 |
| S10 | S16 | 0.732 | 0.469 | 0.135 | 0.467 | 0.823 | 0.733 | 0.838 | 0.343 | - | - | - | - |
| S10 | S17 | 0.733 | 0.469 | 0.135 | 0.467 | 0.822 | 0.733 | 0.832 | 0.343 | - | - | - | - |
| S10 | S20 | 0.315 | 0.057 | 0.571 | 0.798 | 0.068 | 0.790 | 0.385 | 0.992 | 0.721 | 0.668 | 0.425 | 0.574 |
| S10 | S22 | 0.733 | 0.469 | 0.134 | 0.467 | 0.827 | 0.733 | 0.836 | 0.343 | - | - | - | - |
| S10 | S24 | 0.305 | 0.049 | 0.580 | 0.750 | 0.061 | 0.787 | 0.389 | 0.992 | 0.708 | 0.655 | 0.087 | 0.202 |
| S10 | S25 | - | - | - | - | - | - | - | - | 0.611 | 0.855 | 0.563 | 0.625 |
| S10 | S27 | 0.165 | 0.008 | 0.876 | 0.903 | 0.817 | 0.771 | 0.727 | 0.881 | 0.222 | 0.304 | 0.884 | 0.808 |
| S10 | S28 | 0.995 | 0.243 | 0.196 | 0.015 | 0.084 | 0.110 | 0.794 | 0.945 | **0.048** | **0.047** | 0.248 | 0.748 |
| S10 | S29 | 0.075 | 0.008 | 0.997 | 0.852 | 0.836 | 0.771 | 0.670 | 0.896 | 0.596 | 0.721 | 0.987 | 0.624 |
| S13 | S14 | - | - | - | - | - | - | 0.150 | 0.146 | - | - | - | - |
| S13 | S15 | - | - | - | - | - | - | 0.308 | 0.885 | - | - | - | - |
| S13 | S16 | - | - | - | - | - | - | 0.149 | 0.146 | - | - | - | - |
| S13 | S17 | - | - | - | - | - | - | 0.142 | 0.146 | - | - | - | - |
| S13 | S20 | - | - | - | - | - | - | 0.312 | 0.931 | - | - | - | - |
| S13 | S22 | - | - | - | - | - | - | 0.149 | 0.146 | - | - | - | - |
| S13 | S24 | - | - | - | - | - | - | 0.320 | 0.931 | - | - | - | - |
| S13 | S27 | - | - | - | - | - | - | 0.377 | 0.609 | - | - | - | - |
| S13 | S28 | - | - | - | - | - | - | 0.197 | 0.944 | - | - | - | - |
| S13 | S29 | - | - | - | - | - | - | 0.345 | 0.632 | - | - | - | - |
| S14 | S15 | - | - | 0.187 | 0.675 | 0.247 | 0.848 | 0.330 | 0.344 | - | 0.671 | - | 0.908 |
| S14 | S16 | 0.494 | 0.725 | 0.389 | 0.234 | 0.887 | 0.715 | 0.996 | 0.145 | - | - | - | - |
| S14 | S17 | 0.489 | 0.725 | 0.385 | 0.234 | 0.885 | 0.715 | 0.996 | 0.145 | - | - | - | - |
| S14 | S20 | 0.113 | 0.124 | 0.835 | 0.527 | 0.573 | 0.935 | 0.033 | 0.341 | - | 0.370 | - | 0.556 |
| S14 | S22 | 0.486 | 0.725 | 0.395 | 0.234 | 0.881 | 0.715 | 0.995 | 0.145 | - | - | - | - |
| S14 | S24 | 0.104 | 0.107 | 0.835 | 0.491 | 0.512 | 0.935 | 0.032 | 0.341 | - | 0.359 | - | 0.275 |
| S14 | S25 | - | - | - | - | - | - | - | - | - | 0.586 | - | 0.586 |
| S14 | S27 | 0.539 | 0.008 | 0.953 | 0.563 | 0.320 | 0.979 | 0.588 | 0.338 | - | 0.481 | - | 0.588 |
| S14 | S28 | 0.728 | 0.469 | 0.249 | 0.488 | 0.766 | 0.904 | 0.827 | 0.346 | - | 0.643 | - | 0.607 |
| S14 | S29 | **0.036** | **0.007** | 0.915 | 0.730 | 0.437 | 0.935 | 0.637 | 0.340 | - | 0.411 | - | 0.401 |
| S15 | S16 | - | - | 0.220 | 0.596 | 0.245 | 0.848 | 0.336 | 0.344 | - | - | - | - |
| S15 | S17 | - | - | 0.213 | 0.596 | 0.250 | 0.848 | 0.329 | 0.344 | - | - | - | - |
| S15 | S20 | - | - | 0.302 | 0.689 | 0.609 | 0.910 | 0.060 | 0.987 | 0.746 | 0.577 | 0.027 | 0.184 |
| S15 | S22 | - | - | 0.223 | 0.596 | 0.245 | 0.848 | 0.335 | 0.344 | - | - | - | - |
| S15 | S24 | - | - | 0.292 | 0.643 | 0.614 | 0.909 | 0.051 | 0.987 | 0.577 | 0.626 | 0.133 | 0.535 |
| S15 | S25 | - | - | - | - | - | - | - | - | 0.667 | 0.782 | 0.375 | 0.858 |
| S15 | S27 | - | - | 0.939 | 0.906 | 0.471 | 0.978 | 0.006 | 0.884 | 0.560 | 0.739 | 0.731 | 0.927 |
| S15 | S28 | - | - | 0.026 | 0.636 | 0.732 | 0.878 | 0.088 | 0.987 | 0.545 | 0.813 | 0.088 | 0.866 |
| S15 | S29 | - | - | 0.807 | 0.880 | 0.486 | 0.978 | 0.006 | 0.855 | 0.968 | 0.680 | 0.098 | 0.580 |
| S16 | S17 | 0.496 | 0.725 | 1.000 | 0.231 | 0.884 | 0.715 | 0.996 | 0.145 | - | - | - | - |
| S16 | S20 | 0.108 | 0.124 | 0.632 | 0.384 | 0.571 | 0.935 | 0.032 | 0.341 | - | - | - | - |
| S16 | S22 | 0.489 | 0.725 | 1.000 | 0.231 | 0.883 | 0.715 | 0.995 | 0.145 | - | - | - | - |
| S16 | S24 | 0.106 | 0.107 | 0.630 | 0.356 | 0.509 | 0.935 | 0.034 | 0.341 | - | - | - | - |
| S16 | S27 | 0.532 | 0.008 | 0.890 | 0.401 | 0.323 | 0.979 | 0.594 | 0.338 | - | - | - | - |
| S16 | S28 | 0.729 | 0.469 | 0.167 | 0.357 | 0.779 | 0.904 | 0.824 | 0.346 | - | - | - | - |
| S16 | S29 | **0.035** | **0.007** | 0.963 | 0.617 | 0.433 | 0.935 | 0.645 | 0.340 | - | - | - | - |
| S17 | S20 | 0.107 | 0.124 | 0.628 | 0.384 | 0.563 | 0.935 | 0.034 | 0.341 | - | - | - | - |
| S17 | S22 | 0.492 | 0.725 | 1.000 | 0.231 | 0.879 | 0.715 | 0.995 | 0.145 | - | - | - | - |
| S17 | S24 | 0.113 | 0.107 | 0.631 | 0.356 | 0.510 | 0.935 | 0.037 | 0.341 | - | - | - | - |
| S17 | S27 | 0.536 | 0.008 | 0.890 | 0.401 | 0.319 | 0.979 | 0.596 | 0.338 | - | - | - | - |
| S17 | S28 | 0.736 | 0.469 | 0.162 | 0.357 | 0.777 | 0.904 | 0.832 | 0.346 | - | - | - | - |
| S17 | S29 | **0.035** | **0.007** | 0.961 | 0.617 | 0.429 | 0.935 | 0.638 | 0.340 | - | - | - | - |
| S20 | S22 | 0.107 | 0.124 | 0.630 | 0.384 | 0.575 | 0.935 | 0.032 | 0.341 | - | - | - | - |
| S20 | S24 | 0.115 | 0.045 | 1.000 | 0.533 | 0.708 | 0.985 | 1.000 | 0.926 | 0.841 | 0.360 | **0.045** | **0.018** |
| S20 | S25 | - | - | - | - | - | - | - | - | 0.130 | 0.138 | 0.409 | 0.737 |
| S20 | S27 | 0.826 | 0.007 | 0.477 | 0.827 | 0.916 | 0.996 | 0.266 | 0.878 | **0.002** | **0.004** | 0.098 | 0.122 |
| S20 | S28 | 0.310 | 0.057 | 0.342 | 0.601 | 0.154 | 0.970 | 0.427 | 0.994 | 0.961 | 0.618 | 0.789 | 0.714 |
| S20 | S29 | 0.620 | 0.007 | 0.356 | 0.783 | 0.943 | 0.987 | 0.190 | 0.852 | 0.304 | 0.673 | 0.838 | 0.867 |
| S22 | S24 | 0.105 | 0.107 | 0.627 | 0.356 | 0.516 | 0.935 | 0.031 | 0.341 | - | - | - | - |
| S22 | S27 | 0.529 | 0.008 | 0.891 | 0.401 | 0.316 | 0.979 | 0.596 | 0.338 | - | - | - | - |
| S22 | S28 | 0.733 | 0.469 | 0.170 | 0.357 | 0.787 | 0.904 | 0.829 | 0.346 | - | - | - | - |
| S22 | S29 | **0.035** | **0.007** | 0.964 | 0.617 | 0.438 | 0.935 | 0.629 | 0.340 | - | - | - | - |
| S24 | S25 | - | - | - | - | - | - | - | - | 0.128 | 0.133 | 0.449 | 0.490 |
| S24 | S27 | 0.823 | 0.007 | 0.479 | 0.815 | 0.888 | 0.998 | 0.275 | 0.878 | **0.003** | **0.012** | **0.012** | **0.006** |
| S24 | S28 | 0.312 | 0.049 | 0.335 | 0.558 | 0.140 | 0.969 | 0.426 | 0.994 | 0.947 | 0.607 | 0.575 | 0.615 |
| S24 | S29 | 0.610 | 0.007 | 0.359 | 0.773 | 0.916 | 0.994 | 0.190 | 0.852 | 0.468 | 0.819 | 0.202 | 0.136 |
| S25 | S27 | - | - | - | - | - | - | - | - | 0.398 | 0.687 | 0.740 | 0.758 |
| S25 | S28 | - | - | - | - | - | - | - | - | 0.480 | 0.770 | 0.890 | 0.761 |
| S25 | S29 | - | - | - | - | - | - | - | - | 0.390 | 0.596 | 0.259 | 0.559 |
| S27 | S28 | 0.158 | 0.008 | 0.966 | 0.730 | 0.618 | 0.969 | 0.476 | 0.853 | 0.522 | 0.607 | 0.945 | 0.827 |
| S27 | S29 | 0.596 | 0.002 | 0.223 | 0.540 | 0.563 | 0.963 | 0.705 | 0.654 | **<0.001** | **<0.001** | **0.011** | **0.007** |
| S28 | S29 | 0.075 | 0.008 | 0.696 | 0.755 | 0.599 | 0.969 | 0.425 | 0.870 | 0.396 | 0.331 | 0.849 | 0.665 |

1. Difference in LD were evaluated by the LDcontrast method. [↑](#footnote-ref-2)
2. Haplotype frequencies for each pair of SNPs were compared by UNPHASED. [↑](#footnote-ref-3)
3. SNP pairs showing significant difference in both LD and haplotype frequencies between male and female groups are in bold font. 0.01 < *P* ≤ 0.05 are highlighted in light pink, while *P* ≤ 0.01 are highlighted in deep pink boxes. [↑](#footnote-ref-4)
4. Difference in LD were evaluated by the LDcontrast method. [↑](#footnote-ref-5)
5. Haplotype frequencies for each pair of SNPs were compared by UNPHASED. [↑](#footnote-ref-6)
6. SNP pairs showing significant differences in both LD and haplotype frequencies between control and schizophrenia groups are in bold font. 0.01 < *P* ≤ 0.05 are highlighted in light pink, while *P* ≤ 0.01 are highlighted in deep pink boxes. [↑](#footnote-ref-7)
