# Supplementary material for: A Recombination Hotspot in a Schizophrenia-Associated Region of GABRB2
Source: PLoS One. 2010 Mar 8;5(3):e9547. doi: 10.1371/journal.pone.0009547 (PMC2833194; doi:10.1371/journal.pone.0009547)
Supplement: Table S8 — Primers employed in the present study. (0.06 MB DOC) [file pone.0009547.s010.doc]

**Table S8** Primers empolyed in the present study

1. Primers for SNP genotyping

| DNA fragment | Orientation | Name | Chromosome position (bp)^a^ | | Sequence (5’ – 3’) |
| --- | --- | --- | --- | --- | --- |
|  |  |  | 5’ | 3’ |  |
| For first PCR | Forwards | B2I8-214F | 160,692,860 | 160,692,831 | ATGGAGGAAAGGTCCATATCTAGT |
|  | Reverse | B2I10-219ASR | 160,685,434 | 160,685,455 | ATGATGAGCCTGCCAATACAAT  (ATTGTATTGGCAGGCTCATCAT) |
| For nested PCR | | | | | |
| Fragment A | Forward | B2I8-214F | 160,692,860 | 160,692,831 | ATGGAGGAAAGGTCCATATCTAGT |
|  | Reverse | B2I9-R4N | 160,689,951 | 160,689,970 | CTTAATAGCTGGAAAGGTGAT  (TTACAGACCCACCAAGCAAC) |
| Fragment B | Forward | B2I9-215ASF | 160,691,054 | 160,691,029 | CACATCAGAAAGTTGACCTAGAGTCC |
|  | Reverse | B2I9-218R | 160,688,429 | 160,688,452 | CAGACAATGCCTAATGTCCTCTGG  (CCAGAGGACATTAGGCATTGTCTG) |
| For sequencing | | | | | |
| Fragment A | --- | B2I9-R1N | 160,692,286 | 160,692,309 | AAACACTATCCAATAACGCATCCT |
|  | --- | B2I8-214R | 160,692,100 | 160,692,123 | CCTCTAAGCTGTAATCGGAAGGTA |
|  | --- | B2I8-R2N | 160,691,318 | 160,691,337 | CCTAATGGGGGAGTTTGAAC |
|  | --- | B2I9-R4N | 160,689,970 | 160,689,951 | TTACAGACCCACCAAGCAAC |
| Fragment B | --- | B2I8-215ASF | 160,691,054 | 160,691,029 | CACATCAGAAAGTTGACCTAGAGTCC |
|  | --- | B2E9-B2F | 160,690,514 | 160,690,495 | AGGCTGCCAGTGCCAACAAT |
|  | --- | B2I9-217FR | 160,690,422 | 160,690,397 | GAATGCTAGCAATACAGGTTGATGAC |
|  | --- | B2I9-218F | 160,689,486 | 160,689,470 | AGCACTTGCTGCACTAA |

^a^Based on chromosome 5 contig NT_023133.12.

1. Primers for Allele-specific real-time PCR

| SNP to detect | Allele | Primer Name | Sequence (5’ – 3’) |
| --- | --- | --- | --- |
| S3 | G | AS1816071GF1 | AGGAAAATGTCTCAAAAT*G*A*C*G |
| S5 | C | AS1816072CF1 | TCATTCCAATGGCAACT*C*T*A*C |
|  | T | AS1816072TF1 | GTCATTCCAATGGCAACT*C*T*A*T |
| S15 | G | AS13178374GR1 | CTTCACGAGGTCAGGA*G*T*T*C |
| - | - | B2I8-ASPA-F1 | CAGGATGCGTTATTGGATAGTGTT |
| - | - | B2I8-ASPA-R1 | TTGGAATGAGAATCTTTCTGACTCA |
|  | - | B2I8-ASPA-T1 | 6FAM-ACTTCCCACTGTCAAGAGATACCAACTCAACAGG-MGBNFQ |

The asterisked residues were phosphorothioate-bonded. 6FAM, 6-carboxylfluorescein; MGBNFQ, minor groove binder and non-fluorescent quencher.
